# Palmitoylation-driven upregulation of NKAα2 improves diabetic cardiomyopathy: a role for the fumarate hydratase/fumarate metabolic pathway

**DOI:** 10.1038/s41420-026-03275-9

**Published:** 2026-07-29

**Authors:** Xi-Xi Lu, Yu-Yan Feng, Le Yang, Ming-Tao Yang, Hao-Ting Liu, Si-Ping Xiong, Jian Zhu, Ping-Xi Xiao, Hai-Jian Sun, Qing-Bo Lu

**Affiliations:** 1 Department of Endocrinology, Affiliated Hospital of Jiangnan University, Jiangnan University, Wuxi, China; 2 MOE Medical Basic Research Innovation Center for Gut Microbiota and Chronic Diseases, School of Medicine, Jiangnan University, Wuxi, China; 3 Department of Basic Medicine, Wuxi School of Medicine, Jiangnan University, Wuxi, Jiangsu China; 4 Department of Pathology, The Eighth Affiliated Hospital of Sun Yat-Sen University, Shenzhen, Guangdong China; 5 Department of Cardiology, Shanghai Dongfang Hospital, Shanghai Tongji University School of Medicine, Shanghai, China; 6 State Key Laboratory of Natural Medicines, China Pharmaceutical University, Nanjing, PR China; 7 Present Address: Department of Cardiology, The Fourth Affiliated Hospital of Nanjing Medical University, Nanjing, PR China

**Keywords:** Cardiac hypertrophy, Stress signalling

## Abstract

The sodium pump Na⁺/K⁺-ATPase (NKA) plays an essential role in maintaining cellular ion homeostasis. Dysregulation of its α2 subunit (NKAα2) has been implicated in cardiovascular and metabolic diseases, yet its contribution to diabetes-induced cardiac dysfunction remains poorly defined. Therefore, this study aimed to elucidate the role of NKAα2 in diabetic cardiomyopathy (DCM). We performed gain-of-function experiments to investigate the effects of NKAα2 on DCM-associated pathological processes. Both in vivo mouse models and in vitro cardiomyocyte systems were employed to ensure mechanistic consistency. We found that both NKA activity and NKAα2 protein, rather than its transcriptional level, were significantly downregulated in the hearts of diabetic mice and high glucose/palmitic acid (HG/PA)-incubated NRCMs. Cardiac-specific overexpression of NKAα2 markedly alleviated myocardial injury and improved cardiac function in diabetic mice. Consistently, NKAα2 overexpression attenuated HG/PA–induced hypertrophy and apoptosis in primary neonatal rat cardiomyocytes (NRCMs). Mechanistically, ATP-1 mediated de-palmitoylation increased NKAα2 in the mitochondrial-enriched fraction, where it interacted with E3 ubiquitin protein ligase 1 (MUL1), which was responsible for NKAα2 ubiquitination and degradation. Metabolomic analyses revealed that NKAα2 downregulation dissociated Mul1 from fumarate hydratase (FH), leading to increased FH expression and decreased intracellular fumarate levels. Exogenous fumarate administration mimicked the cardioprotective effects of NKAα2 overexpression and mitigated the damage caused by NKAα2 deficiency in HG/PA-treated cardiomyocytes. Overexpression of FH blocked the effects of NKAα2 on diabetic cardiac dysfunction. Collectively, these findings identify NKAα2 as a critical regulator in DCM. These results also underscore the requirement of tightly controlled FH activity and fumarate metabolism for NKAα2-dependent cardioprotection.

**Figure:**
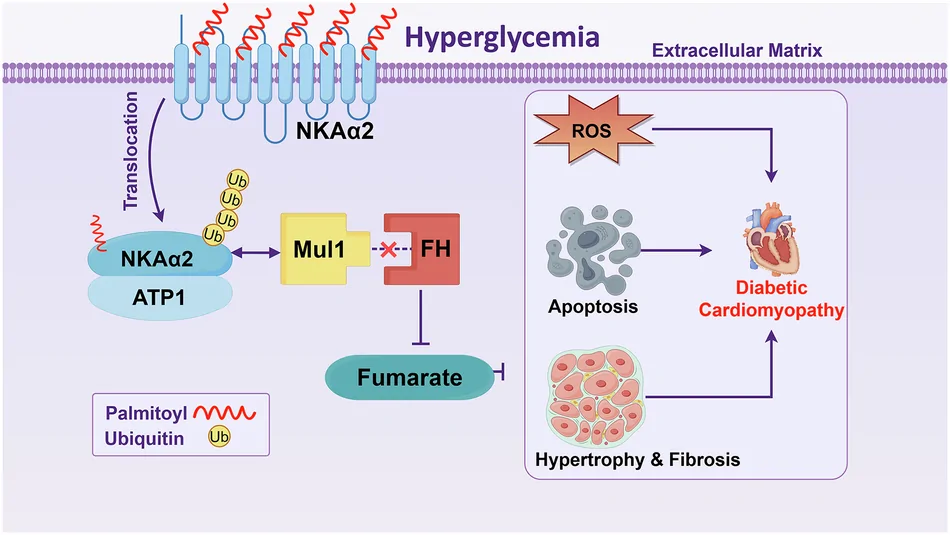
A diagram illustrating the molecular mechanism of the NKAα2/FH/fumarate metabolic axis in the development of DCM.

A diagram illustrating the molecular mechanism of the NKAα2/FH/fumarate metabolic axis in the development of DCM.

## Introduction

Diabetic cardiomyopathy (DCM), a common diabetes-related complication and a major contributor to heart failure, is characterized by left ventricular dysfunction that develops independently of atherosclerosis, coronary artery disease, or hypertension [1, 2]. This distinction defines DCM as a unique clinical entity rather than a secondary consequence of vascular disease [3, 4]. Importantly, cardiac dysfunction in patients with diabetes is often clinically silent and remains undetected until advanced stages. Epidemiological studies estimate that nearly half of asymptomatic, normotensive individuals with well-controlled diabetes already exhibit measurable cardiac dysfunction [5]. These findings underscore the substantial burden of subclinical myocardial injury in diabetes. Despite its high prevalence, effective therapeutic strategies for DCM remain limited [6]. Whether structural remodeling precedes functional impairment, or vice versa, remains unresolved [7, 8]. Multiple pathogenic mechanisms have been implicated in the development of DCM. These include excessive production of reactive oxygen species, aberrant activation of poly(ADP-ribose) polymerase and protein kinase C, accumulation of advanced glycation end products, dysregulated fatty acid metabolism, and sustainable activation of the renin–angiotensin system [9, 10**]**. Dysregulation of the innate immune system and persistent low-grade inflammation are now recognized as central drivers of DCM onset and progression [11–14]. These insights link metabolic stress to sustained inflammatory signaling in the hearts of diabetic models. Nevertheless, elucidating the underlying pathophysiological mechanisms of diabetic heart disease is essential. Such insight is critical for identifying novel targets and developing effective interventions to prevent progression to heart failure.

Na⁺/K⁺-ATPase (NKA) is a multifunctional membrane enzyme that maintains cellular sodium and potassium gradients [15, 16]. Beyond ion transport, NKA also participates in intracellular signal transduction [17]. Owing to its broad tissue distribution, NKA is essential for physiological function in the heart, brain, liver, and kidneys [18–23]. Structurally, NKA is composed of α, β, and γ subunits [24]. Four α-subunit isoforms have been identified to date, each with distinct tissue expression patterns, including the α1, α2, α3, and α4 isoforms [25, 26]. Phospholemman (PLM) serves as an essential molecular scaffold that integrates PKC and PKA phosphorylation signals to synergistically upregulate NKA ion transport activity, thereby establishing the central regulatory role of NKA in cardiac excitation-contraction coupling and cellular ion homeostasis [27]. Activation of NKA induces both positive inotropic and cardioprotective effects via the Src/PI3K/Akt/ERK1/2 pathways [28]. The activity of NKA is lower in the hearts of diabetic rats [29] and doxorubicin-induced rats [30, 31]. NKA/c-Src-dependent NADPH oxidase/ROS pathway is involved in high glucose (HG)-induced myocardial cell injury [32], and activation of NKA is sufficient to reverse HG-induced oxidative injury in cardiomyocytes [33]. Advanced glycation end-products (AGE) impair NKA stability by regulating the AMPK/SIRT1 pathway during progression of DCM [34]. In the context of diabetes, calpain-1 activation induces cardiomyocyte apoptosis by downregulating NKA activity, contributing to DCM and highlighting its potential as a therapeutic target [35]. Downregulation of NKAα2 is observed in rodents with chronic heart failure [36]. Diabetes impairs the insulin-stimulated translocation of NKA, especially the α2 isoform, to the plasma membrane in cardiac myocytes via a PI3K-dependent pathway [37]. In cardiac myocytes, NKAα2 plays a more prominent role than NKA-α1 in regulating sarcoplasmic reticulum Ca²⁺ release, a function mediated by its specific localization in junctional membrane domains rather than through global cytosolic [Na⁺] changes [38]. The NKAα2 isoform is predominantly localized to cardiac t-tubules where it functionally couples with the Na⁺/Ca²⁺ exchanger (NCX) to regulate local Ca²⁺ handling and contractility without affecting global intracellular Na⁺ levels [39]. Interestingly, cardiac-specific overexpression of NKAα2, but not NKAα1, confers significant protection against pressure overload-induced hypertrophy [40]. Together, it is highly probable that NKA functions as a crucial player in the pathologies of DCM. Thus, this study aimed to clarify whether the different isoforms of NKA protected against DCM, and if so, to investigate the underlying molecular mechanisms involved.

In this study, we found that NKA activity and the translational level of NKAα2 were obviously downregulated in the hearts from STZ-treated mice and cultured NRCMs exposed to high glucose/palmitic acid (HG/PA). Cardiac-specific overexpression of NKAα2 ameliorated pathological cardiac performance in diabetic mice. Similar results were observed in in vitro NRCMs. Mechanistically, ATP1–mediated depalmitoylation promoted MUL1-dependent ubiquitination of NKAα2 in NRCMs. Metabolomic analyses revealed that NKAα2 downregulation disrupted the interaction between MUL1 and fumarate hydratase (FH), leading to increased FH expression and reduced intracellular fumarate levels. Functionally, exogenous fumarate administration alleviated DCM and mitigated HG/PA-induced NRCM injury caused by NKAα2 deficiency. In contrast, FH overexpression abolished the cardioprotective effects mediated by NKAα2 in DCM. Together, these findings identify an NKAα2/MUL1/FH/fumarate axis that links mitochondrial protein regulation to metabolic remodeling in DCM, highlighting cardiomyocyte NKAα2 as a potential therapeutic target for diabetic cardiac dysfunction.

## Results

### NKAα2 is downregulated in the hearts from STZ-treated mice and HG/PA-exposed NRCMs

**Fig. 1:**
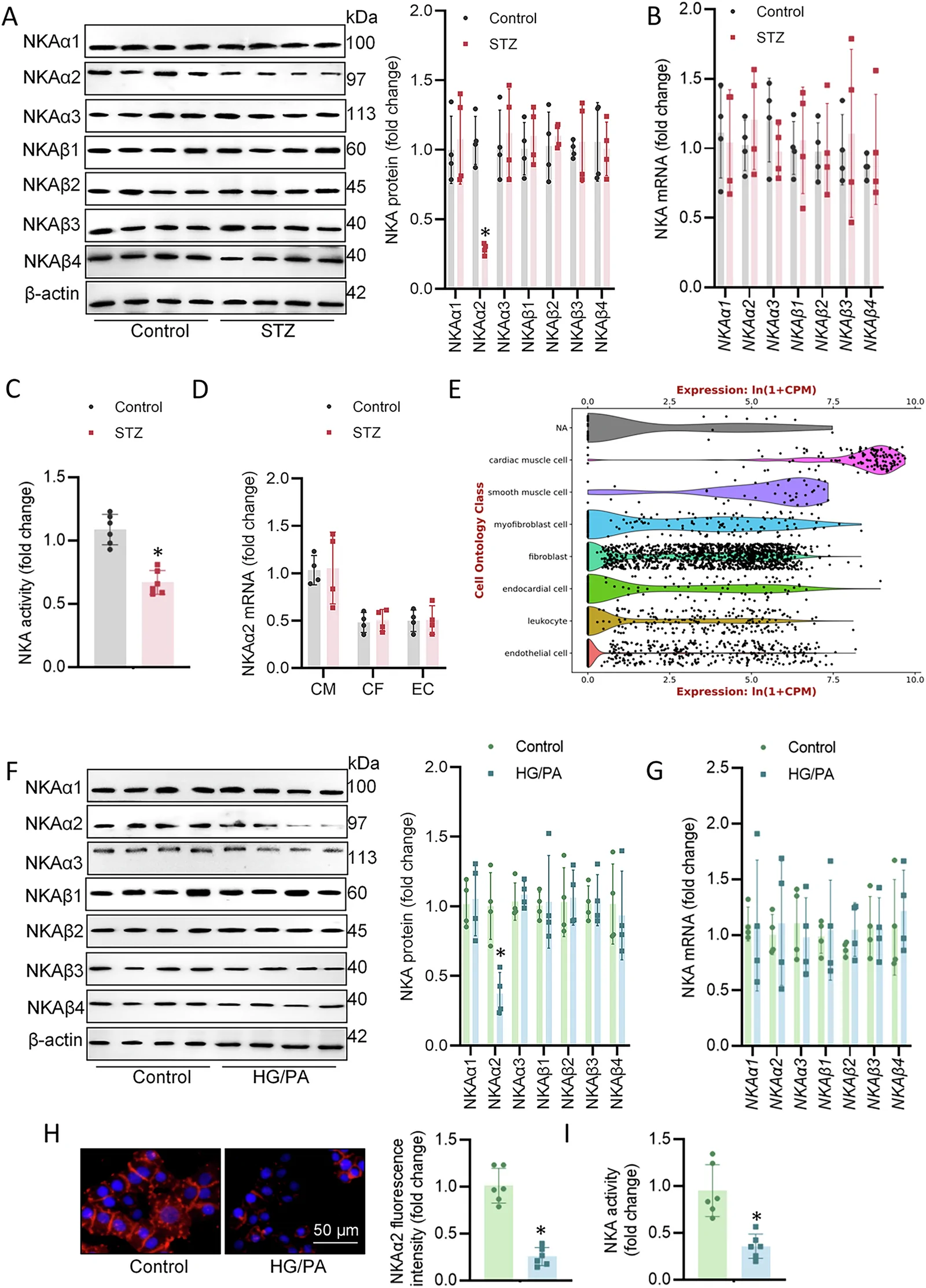
NKAα2 is downregulated in the hearts from STZ-treated mice and HG/PA-exposed NRCMs. **A** Representative blots and quantitative analysis of NKAα1, NKAα2, NKAα3, NKAβ1, NKAβ2, NKAβ3, and NKAβ4 in mouse hearts. **B** Relative mRNA levels of NKAα1, NKAα2, NKAα3, NKAβ1, NKAβ2, NKAβ3, and NKAβ4 in mouse hearts. **C** NKA activity in normal hearts and the hearts from STZ-treated mice. **D** Relative mRNA levels of NKAα2 in isolated cardiomyocytes, cardiac fibroblasts, and endothelial cells from normal hearts and the hearts from STZ-treated mice. **E** Single-cell sequencing of mouse hearts revealed that NKAα2 was expressed mainly in cardiomyocytes from the Tabula Muris database. **F** Representative blots and quantitative analysis of NKAα1, NKAα2, NKAα3, NKAβ1, NKAβ2, NKAβ3, and NKAβ4 in HG/PA-exposed NRCMs. **G** Relative mRNA levels of NKAα1, NKAα2, NKAα3, NKAβ1, NKAβ2, NKAβ3, and NKAβ4 in HG/PA-exposed NRCMs. **H** Representative immunofluorescence staining for NKAα2 in cultured NRCMs. **I** NKA activity in normal and HG/PA-exposed NRCMs. Values were expressed as mean ± SD. The difference between two groups was statistically analyzed by Student’s *t* test. *P < 0.05 *vs*. Control.

To investigate the potential role of NKA in DCM, we assessed the activity and expression of NKA in cardiac tissues in STZ-induced type 1 diabetic mice and HG/PA-exposed NRCMs. Western blots revealed that the protein expression of NKAα2 was significantly downregulated in cardiac tissue from STZ-induced diabetic mice, rather than NKAα1, NKAα3, and NKAβ1-4 (Fig. 1A). Interestingly, the mRNA levels of NKAα1-3 and NKAβ1-4 remain unchanged in the hearts from diabetic mice (Fig. 1B). The reduction of NKAα2 protein was accompanied by decreased NKA activity in STZ-induced hearts (Fig. 1C). Consistently, the mRNA level of NKAα2 remained unaltered in isolated cardiomyocytes, cardiac fibroblasts, and endothelial cells from the myocardium under STZ-induced diabetic conditions (Fig. 1D). Single-cell sequencing of mouse hearts revealed that NKAα2 was expressed mainly in cardiomyocytes from the Tabula Muris database (Fig. 1E). We then used the online database HUMAN PROTEIN ATLAS (https://www.proteinatlas.org/) to compare the differences in NKAα2 expression levels in different human cells, and the results showed that NKAα2 is mainly expressed in cardiomyocytes (Supplementary Fig. S1A). In the human hearts, NKAα2 is primarily detected in cardiomyocytes (Supplementary Fig. S1B, C). Thus, we focused on studying the role of cardiomyocytes-derived NKAα2 in diabetes-elicited cardiac dysfunction. In support, the downregulated protein expression of NKAα2 was ascertained by double immunofluorescence staining in diabetic mouse hearts (Supplementary Fig. S2A). Immunofluorescence using IgG as a control confirmed the specificity of the observed immunofluorescence signals (Supplementary Fig. S2B). Notably, similar alterations in NKAα2 were observed in HG/PA-exposed NRCMs (Fig. 1F–I). These results collectively indicated that NKAα2 was selectively and post-transcriptionally downregulated in cardiomyocytes under diabetic conditions, leading to impaired NKA function.

### Overexpression of NKAα2 ameliorates cardiac injury and dysfunction in diabetic mice

**Fig. 2:**
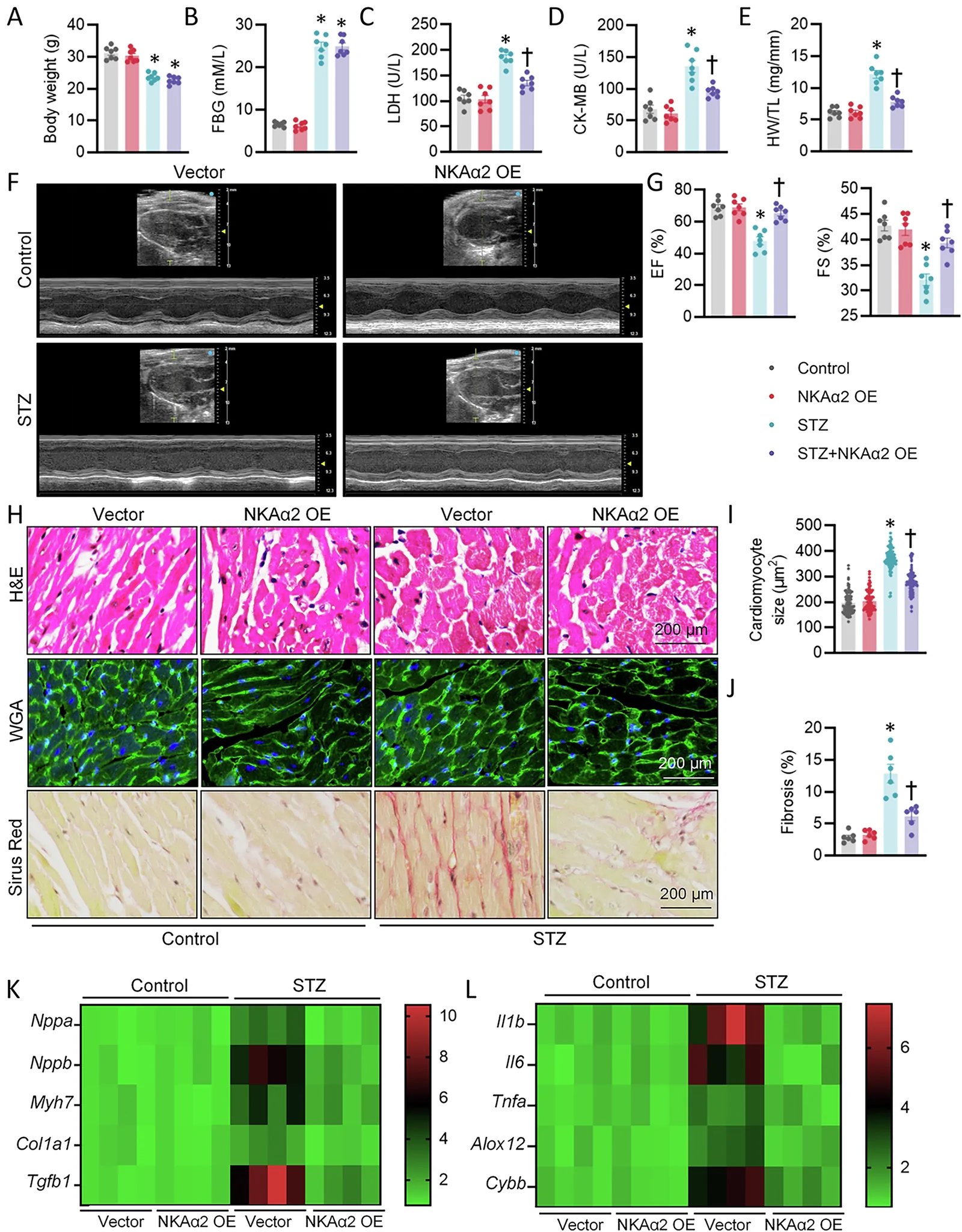
Overexpression of NKAα2 ameliorates cardiac injury and dysfunction in diabetic mice. **A** Body weight. **B** Fasting blood glucose (FBG). **C** Serum LDH levels. **D** Serum CK-MB levels. **E** The heart weight–to–tibia length ratio (HW/TL). **F** Representative images of echocardiography in different groups. **G** EF and FS. **H** Representative images of H&E staining, WGA staining, and Sirius Red staining in cardiac sections. **I** Quantitative analysis of cardiomyocyte size. **J** Quantitative analysis of cardiac fibrosis. **K** Relative mRNA levels of hypertrophic and fibrotic markers in the hearts, including *Nppa*, *Nppb*, *Myh7*, *Tgfb1*, and *Col1a1*. **L** Relative mRNA levels of *Il1b*, *Il6*, *Tnfa*, *Alox12*, and *Cybb*. Values were expressed as mean ± SD. The differences among the four groups were statistically analyzed by two-way ANOVA with Tukey’s multiple comparisons test. *P < 0.05 *vs*. Control. †*P* < 0.05 *vs*. STZ.

To examine the role of cardiomyocyte NKAα2 in the etiologies of DCM, we overexpressed NKAα2 using cardiotropic adeno-associated virus 9 (AAV9) driven by the cTnT promoter. Six weeks after the first injection of STZ, mice received AAV9- NKAα2 via tail-vein injection. Isolation of cardiac cells from mice indicated that AAV9 induced robust and persistent upregulations of NKAα2 and NKA activity in the mouse hearts (Supplementary Fig. S3A, B). NKAα2 overexpression did not alter body weight or serum glucose/lipid levels, indicating no systemic metabolic effects (Fig. 2A, B and Supplementary Table S3). Serum levels of cardiac injury markers, including lactate dehydrogenase (LDH) and creatine kinase-cardiac (CK-MB), did not show the same increase as seen in untreated diabetic mice, indicating that NKAα2 restoration attenuated diabetic cardiac damage (Fig. 2C, D). Moreover, NKAα2 overexpression prevented cardiac hypertrophy, as evidenced by a decreased heart weight–to–tibia length ratio (HW/TL) (Fig. 2E). Consistently, echocardiographic measurements showed marked improvement in cardiac function in mice with cardiac-specific overexpression of NKAα2, reflecting protection against diabetes-induced dysfunction (Fig. 2F, G and Supplementary Table S3). Histological analyses further demonstrated decreased myocardial hypertrophy and fibrosis following AAV9-NKAα2 treatment (Fig. 2H–J). These structural improvements were supported by reduced expression of hypertrophic and fibrotic markers in the hearts, including *Nppa*, *Nppb*, *Myh7*, *Tgfb1*, and *Col1a1* (Fig. 2K). RT-qPCR assay for *Il1b*, *Il6*, *Tnfa*, *Alox12*, and *Cybb* mRNAs in mouse heart tissues revealed that AAV9-NKAα2 injection prevented inflammation and oxidative stress in the hearts from STZ-treated mice (Fig. 2L). Also, immunofluorescence staining for F4/80 and TUNEL staining showed that NKAα2 overexpression protected hearts against STZ-induced inflammation and death in the hearts (Supplementary Fig. S3C–E). In keeping with RT-qPCR results, overexpression of NKAα2 lowered serum levels of IL-1β, IL-6, and TNF-α in diabetic mice (Supplementary Fig. S3F). Collectively, these findings establish cardiomyocyte NKAα2 as a key protective factor against DCM.

### NKAα2 mitigates cardiomyocyte injury induced by HG/PA in cardiomyocytes

**Fig. 3:**
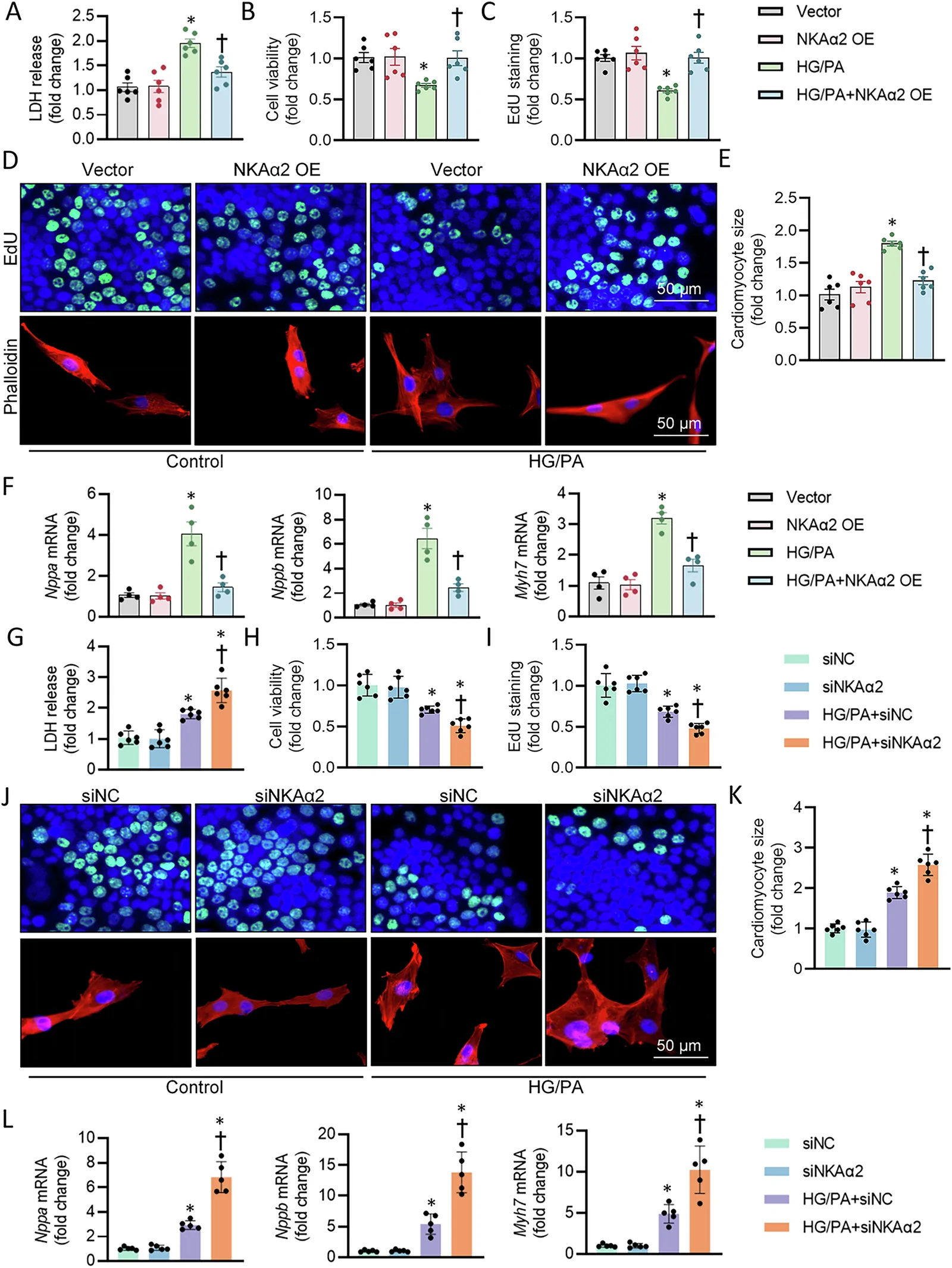
NKAα2 mitigates injury induced by HG/PA in NRCMs. **A** Effects of NKAα2 knockdown on LDH release. **B** Effects of NKAα2 knockdown on cell viability assessed by CCK-8 assay. **C** Effects of NKAα2 knockdown on cell viability assessed by EdU staining. **D** Representative images of EdU staining and Phalloidin staining after knockdown of NKAα2. **E** Quantitative analysis of cardiomyocyte size after knockdown of NKAα2. **F** Relative mRNA levels of *Nppa*, *Nppb*, and *Myh7* after knockdown of NKAα2. **G** Effects of NKAα2 overexpression on LDH release. **H** Effects of NKAα2 overexpression on cell viability assessed by CCK-8 assay. **I** Effects of NKAα2 overexpression on cell viability assessed by EdU staining. **J** Representative images of EdU staining and Phalloidin staining after overexpression of NKAα2. **K** Quantitative analysis of cardiomyocyte size after overexpression of NKAα2. **L** Relative mRNA levels of *Nppa*, *Nppb*, and *Myh7* after overexpression of NKAα2. Values were expressed as mean ± SD. The differences among the four groups were statistically analyzed by two-way ANOVA with Tukey’s multiple comparisons test. *P < 0.05 *vs*. Vector or siNC. †*P* < 0.05 *vs*. HG/PA or HG/PA+siNC.

To further define the cardioprotective role of NKAα2, we examined its effects on HG/PA–induced injury in NRCMs. NRCMs were identified by immunofluorescence staining of α-actinin (Supplementary Fig. S4A, B). Compared with the control group, HG/PA treatment significantly reduced NKA activity, NKAα2 transcription, and cell viability in NRCMs. In contrast, the osmotic control, BSA alone, or their combination had no significant effects on these parameters (Supplementary Fig. S4C–E). These findings suggest that HG/PA-induced impairment of NKA function and cell viability in NRCMs is driven by glucolipotoxicity rather than by nonspecific osmotic or vehicle-related effects. NKAα2 was overexpressed in NRCMs, as confirmed by Western blot, RT-qPCR, and NKA activity in Supplementary Fig. S4F–H. NKAα2 overexpression reduced NRCM death, as evidenced by decreased LDH release (Fig. 3A), restored cellular viability (Fig. 3B), and more EdU-positive cells (Fig. 3C, D). Morphological analysis by phalloidin staining demonstrated that NKAα2 overexpression alleviated HG /PA–induced NRCM hypertrophy (Fig. 3D, E). NKAα2 overexpression markedly suppressed HG/PA–induced increases in the mRNA levels of hypertrophic markers *Nppa*, *Nppb*, and *Myh7* (Fig. 3F). Conversely, siRNA-mediated knockdown of NKAα2 (Supplementary Fig. S4I–K) exacerbated HG/PA–induced injury and hypertrophy in cultured NRCMs (Fig. 3G–L). Together, these gain- and loss-of-function studies demonstrate that NKAα2 protects NRCMs from HG/PA–induced hypertrophy and cell death.

### Depalmitoylation increased NKAα2 in the mitochondrial-enriched fraction and ubiquitination of NKAα2

**Fig. 4:**
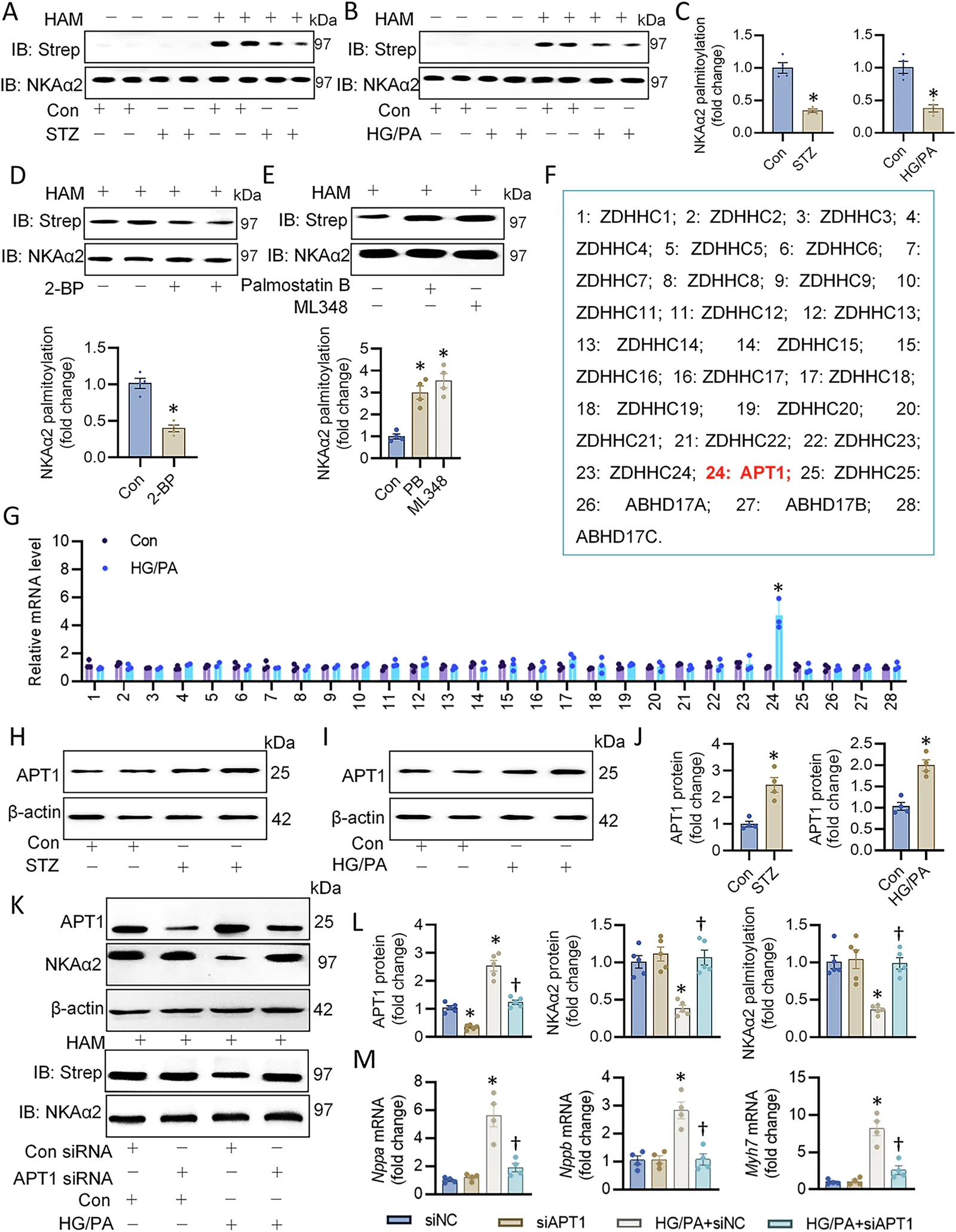
Depalmitoylation of NKAα2 is observed in the hearts from STZ-treated mice and HG/PA-induced NRCMs. **A**, **C** The palmitoylated NKAα2 protein was significantly downregulated in the hearts of mice with diabetes. **B**, **C** The palmitoylated NKAα2 protein was significantly downregulated in HG/PA-challenged NRCMs. **D** 2-bromopalmitate (2-BP), a general palmitoylation inhibitor, reduced the palmitoylation of NKAα2. **E** The palmitoylation of NKAα2 was enhanced using the selective inhibitors of APT1, palmostatin B and ML348. **F**, **G** Relative mRNA levels of DHHC family members. **H**–**J** The protein expression of APT1 was strikingly upregulated in the hearts from STZ-treated mice and HG/PA-induced NRCMs. **K**, **L** Knockdown of APT1 restored NKAα2 protein levels, in conjunction with higher palmitoylation level of NKAα2. **M** Silencing APT1 lowered the mRNA levels of hypertrophic genes, including *Nppa*, *Nppb*, and *Myh7*. Values were expressed as mean ± SD. The difference between the two groups (**A**–**J**) was statistically analyzed by Student’s *t* test. The differences among the four groups (**L**, **M**) were statistically analyzed by two-way ANOVA with Tukey’s multiple comparisons test. *P < 0.05 *vs*. Con or siNC. †*P* < 0.05 *vs*. HG/PA+siNC.

Protein palmitoylation has emerged as a key post-translational modification (PTM) in cardiovascular biology [41]. This modification dynamically controls protein membrane localization, stability, trafficking, and signaling, and dysregulated palmitoylation is increasingly linked to cardiovascular diseases, including DCM [42]. To identify the palmitoylation site of NKAα2, we employed the motif-based predictors CSS-palm 4.0 [43, 44]. This model predicted a single palmitoylation site of NKAα2 within its domain (Cys462) based on a defined palmitoylation motif (Supplementary Fig. S5A). The palmitoylation level of cardiac NKAα2 in diabetic mice was then investigated by immunoprecipitation and acyl-biotin exchange (IP-ABE) method. The results of IP-ABE indicated that palmitoylated NKAα2 protein was significantly downregulated in the hearts of mice with diabetes when compared to normal mice (Fig. 4A, C). In addition, HG/PA inhibited the palmitoylation of NKAα2 in cultured NRCMs (Fig. 4B, C). When primary NRCMs were treated with 2-bromopalmitate (2-BP), a general palmitoylation inhibitor, the palmitoylation of NKAα2 was markedly diminished (Fig. 4D). Oppositely, enhanced palmitoylation of NKAα2 was detected using the selective inhibitors of acyl protein thioesterase 1 (APT1), palmostatin B and ML348 (Fig. 4E). To identify the principal palmitoyltransferases responsible for NKAα2 palmitoylation in DCM, we examined the expression patterns of all DHHC family members in the hearts from STZ-treated mice. Results showed that the transcriptional level of APT1 was remarkably upregulated in the hearts of diabetic mice (Fig. 4F, G). In line with this, the protein expression of APT1 was strikingly upregulated in the hearts from STZ-treated mice and HG/PA-induced NRCMs (Fig. 4H–J). APT1, a key depalmitoylating enzyme, dynamically regulates the membrane localization and signaling duration of palmitoylated proteins, and its dysregulation contributes to metabolic stress-induced cardiovascular dysfunction [45]. Thus, we hypothesized that APT1 might lead to decreased NKAα2 palmitoylation in the context of DCM. As anticipated, knockdown of APT1 restored NKAα2 protein level, in conjunction with higher palmitoylation levels of NKAα2 (Fig. 4K, L). Interestingly, inhibition of NKAα2 palmitoylation by mutations of palmitoylation sites (C462A) inhibited HG/PA-elicited downregulation of NKAα2 protein and palmitoylation (Supplementary Fig. S5B, C). Importantly, silencing APT1 lowered the mRNA levels of hypertrophic genes, including *Nppa*, *Nppb*, and *Myh7* (Fig. 4M). These results indicate that APT1-mediated depalmitoylation is a critical mechanism driving the loss of NKAα2 palmitoylation and protein stability in DCM, thereby contributing to cardiomyocyte hypertrophy under metabolic stress.

**Fig. 5:**
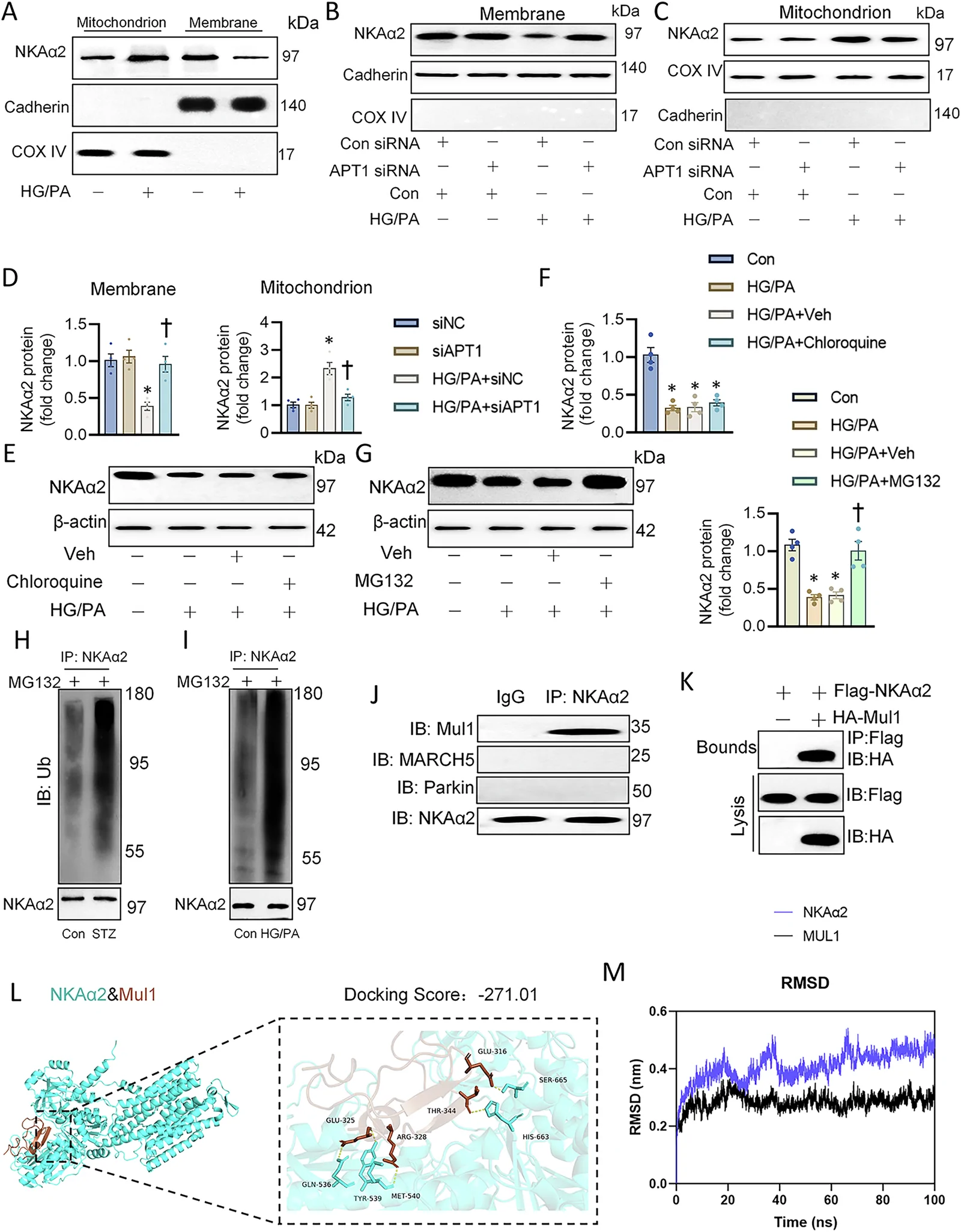
NKAα2 undergoes ubiquitination in DCM. **A** The mitochondrial and membrane abundance of NKAα2 in normal and HP/PA-exposed NRCMs. **B**–**D** The expression of NKAα2 in the plasma membrane and mitochondria in APT1-deficient NRCMs. **E**–**G** The lysosomal inhibitor chloroquine showed no detectable effect on this degradation. In contrast, pretreatment with the proteasome inhibitor MG-132 significantly attenuated HG/PA-induced NKAα2 degradation. **H**, **I** The ubiquitination level of NKAα2 in the hearts from STZ-treated mice and in HG/PA-treated NRCMs. **J**, **K** An interaction between Mul1 and NKAα2. **L** The molecular docking indicated that NKAα2 and Mul1 were capable of forming a stable complex, with a docking score of -271.01. **M** The root-mean square deviation (RMSD) showing the interaction of Mul1 with NKAα2. Values were expressed as mean ± SD. The differences among the four groups (**D**) were statistically analyzed by two-way ANOVA with Tukey’s multiple comparisons test. The differences among the four groups (**F**, **G**) were statistically analyzed by one-way ANOVA with Bonferroni’s post-hoc test. *P < 0.05 *vs*. Con or siNC. †*P* < 0.05 *vs*. HG/PA+siNC or HG/PA+Veh.

APT1 is predominantly localized at mitochondria and serves as a putative mitochondrial *S*-deacylase regulating mitochondrial S-depalmitoylation [46]. Given that palmitoylation modification is closely associated with the subcellular localization of proteins [47]. We hypothesized that diabetic stress might alter the subcellular distribution of NKAα2 and that this process might be associated with APT1-mediated depalmitoylation. To examine this possibility, we isolated plasma membrane and mitochondrial-enriched fractions from NRCMs. The results showed that HG/PA challenge decreased the abundance of NKAα2 in the plasma membrane fraction, while increasing its level in the mitochondrial-enriched fraction (Fig. 5A, B and Supplementary Fig. S6A). Notably, this HG/PA-associated redistribution of NKAα2 was attenuated in APT1-deficient NRCMs (Fig. 5C, D). Similarly, transfection with the C462A mutant stabilized the localization of NKAα2 at the plasma membrane (Supplementary Fig. S5D, E). Considering that the aforementioned results showed no change in the transcriptional level of NKAα2 under DCM conditions, while its protein level was significantly reduced, this implicates that its expression may be regulated by post-translational modifications. Intracellular protein degradation primarily occurs via two pathways: the ubiquitin–proteasome system and the lysosomal pathway [48]. To determine which pathway mediates diabetes-induced NKAα2 degradation, NRCMs were treated with specific inhibitors of each system. As shown in Fig. 5E, F, the lysosomal inhibitor chloroquine showed no detectable effect on this degradation. In contrast, pretreatment with the proteasome inhibitor MG-132 significantly attenuated HG/PA-induced NKAα2 degradation (Fig. 5G). Together, these findings indicate that diabetes promotes NKAα2 degradation predominantly through the ubiquitin–proteasome pathway. As expected, the ubiquitination level of NKAα2 was significantly increased in the hearts from STZ-treated mice and in HG/PA-treated NRCMs (Fig. 5H, I). This increase was blocked by the C462A mutation (Supplementary Fig. S5F), suggesting that palmitoylation mediates the ubiquitination-dependent degradation of NKAα2. Meanwhile, the C462A mutation suppressed HG/PA-induced NRCM injury (Supplementary Fig. S5G) and the increase in cell surface area (Supplementary Fig. S5H–J). These results collectively indicate that APT1-dependent depalmitoylation governs the subcellular relocalization, ubiquitination, and proteasomal degradation of NKAα2 under diabetic conditions, thereby contributing to cardiomyocyte injury. Elucidating the molecular mechanism underlying the ubiquitination of NKAα2 within mitochondria is of significant interest. We thus screened a panel of E3 ubiquitin ligases that potentially interact with NKAα2 and identified a significant interaction between Mul1 and NKAα2 (Fig. 5J, K). The molecular docking indicated that NKAα2 and Mul1 were capable of forming a stable complex, with a docking score of -271.01 (Fig. 5L). To investigate the interaction between NKAα2 and Mul1 during the simulation and to evaluate the stability of the binding site, we performed a 100 ns molecular dynamics (MD) simulation on this protein complex. The dynamic stability of the system was then verified. The root-mean square deviation (RMSD) is a key metric for assessing whether a complex system has reached equilibrium. As shown in Fig. 5M, the RMSD of the complex system began to stabilize after approximately 30 ns of MD simulation. The root-mean square fluctuation (RMSF) is used to evaluate the stability of amino acid residues by measuring their positional variability throughout the simulation. The RMSF values for each residue in the complex systems are presented in Supplementary Fig. S7A. Both systems exhibited nearly identical fluctuation trends. Notably, slight differences in RMSF values were observed in certain residues, primarily attributable to variations in binding affinity. All RMSF values remained below 0.8 nm, which is relatively low in comparison with most related studies, indicating high rigidity and dynamic stability of the protein complex of NKAα2/Mul1. Therefore, we conclude that the protein maintains structural stability during functional dynamics, demonstrating considerable adaptability and robustness. The radius of gyration (Rg) reflects the compactness of a protein. As shown in Supplementary Fig. S7B, the Rg values of both systems gradually decreased over the simulation time. The Rg of the NKAα2 system stabilized within 3.99–4.01 nm, while that of the Mul1 system stabilized within 1.02–1.05 nm. This observation suggests that the protein exhibited favorable flexibility and extensibility during the simulation. Hydrogen bonds play a critical role in stabilizing protein–ligand interactions. MD simulations revealed a progressive reduction in solvent-accessible surface area (SASA) accompanied by a sustained increase in the number of hydrogen bonds (Hnum) during the simulation, indicating stable complex formation between NKAα2 and Mul1 (Supplementary Fig. S7C, D). These findings suggest that both interface burial and persistent hydrogen bonding contribute to the stabilization of the NKAα2–Mul1 interaction. Notably, the interaction of NKAα2 with Mul1 tended to be higher in the hearts from STZ-treated mice and in HG/PA-treated NRCMs (Supplementary Fig. S7E, F). These results indicate that Mul1 functions as a mitochondrial E3 ubiquitin ligase that directly associates with NKAα2 and mediates its ubiquitination under diabetic conditions, and that NKAα2 is an integral component of a Mul1-containing mitochondrial protein complex.

### NKAα2 affects the fumarate hydratase (FH)/fumarate metabolic pathway in DCM

**Fig. 6:**
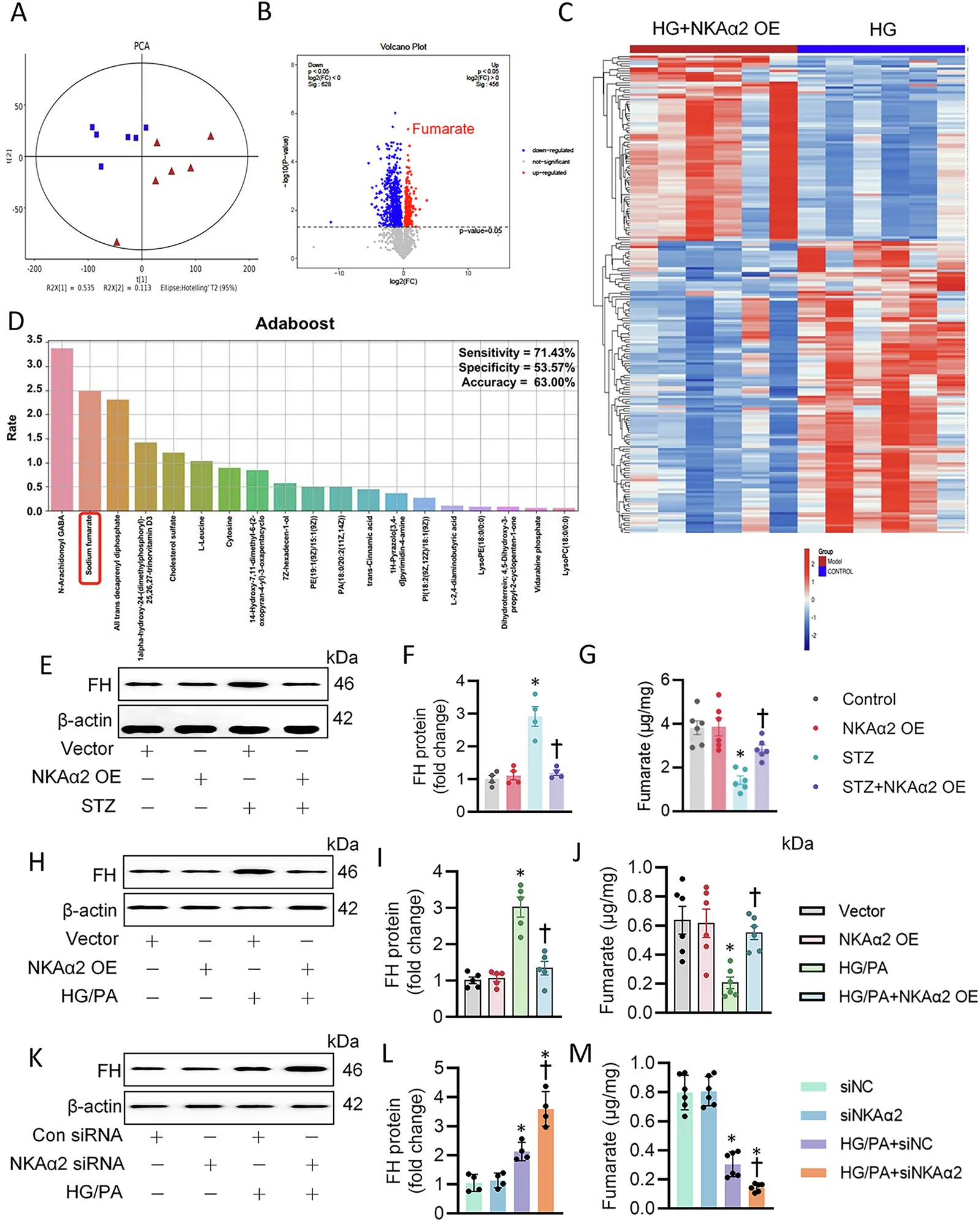
NKAα2 affects the fumarate hydratase (FH)/fumarate metabolic pathway in DCM. **A** Principal component analysis (PCA) revealed distinct clustering of differential metabolites between NKAα2-overexpressing cells and HG/PA-treated cells. **B**, **C** Volcano plot and heatmap showing differential metabolites. **D** Ranking the importance of differential metabolites based on Random Forest and AdaBoost Feature Selection Algorithms. **E**, **F** Representative blots and quantitative analysis of FH in the mouse hearts. **G** Fumarate contents in the mouse hearts. **H**, **I** Representative blots and quantitative analysis of FH in NRCMs after overexpression of NKAα2. **J** Fumarate contents in NRCMs after overexpression of NKAα2. **K**, **L** Representative blots and quantitative analysis of FH in NRCMs after knockdown of NKAα2. **M** Fumarate contents in NRCMs after knockdown of NKAα2. Values were expressed as mean ± SD. The differences among the four groups **E**–**M** were statistically analyzed by two-way ANOVA with Tukey’s multiple comparisons test. *P < 0.05 *vs*. Con, Vector or siNC. †*P* < 0.05 *vs*. STZ, HG/PA or HG/PA+siNC.

As mitochondria are highly metabolically active organelles, we sought to investigate whether the observed reduction in NKAα2 affects mitochondrial metabolism in cardiomyocytes. We performed untargeted metabolomics to profile alterations in metabolites. Principal component analysis (PCA) revealed distinct clustering of differential metabolites between NKAα2-overexpressing cells and HG/PA-treated cells (Fig. 6A). Upon NKAα2 overexpression, 202 metabolites were differentially expressed, of which 124 metabolites were downregulated and 78 metabolites were upregulated (Fig. 6B, C). Among these differentially expressed metabolites, fumarate exhibited the most significant alteration. AdaBoost-based feature selection was applied for metabolomic data mining. After outlier filtering, 19 metabolites showed influence weights exceeding the expected distribution and contributed to sample classification (Fig. 6D). The AdaBoost classifier achieved a sensitivity of 71.43%, a specificity of 53.57%, and an overall accuracy of 63%. Within this model, fumarate ranked second in feature importance, indicating a strong contribution to classification performance (Fig. 6D). Machine learning analysis suggested that the change in fumarate may play an important role in NKAα2-mediated metabolic regulation (Fig. 6D). Fumarate hydratase (FH), also known as fumarase, is a key enzyme of the tricarboxylic acid (TCA) cycle that catalyzes the reversible hydration of fumarate to malate (Supplementary Fig. S8A). By generating cardiac-specific FH knockout mice, Houman et al. have demonstrated that elevated cardiac fumarate protects against ischemia-reperfusion injury by activating the Nrf2 antioxidant pathway [49]. Beyond its canonical metabolic role, the FH/fumarate axis has emerged as an important regulator of mitochondrial function, redox signaling, and protein post-translational modifications, particularly in cardiovascular and metabolic diseases [50–52]. The protein expression of FH was significantly increased in the hearts from STZ-treated mice, accompanied by a decrease in fumarate levels (Fig. 6E–G). This alteration was reversed by NKAα2 overexpression (Fig. 6E–G). At the cellular level, NKAα2 overexpression markedly suppressed FH expression and restored fumarate levels (Fig. 6H–J), whereas NKAα2 knockdown exerted the opposite effect (Fig. 6K–M). Also, downregulation of NKAα2 further suppressed the interaction between FH and Mul1, whereas overexpression of NKAα2 restored their binding in NRCMs exposed to HG/PA (Supplementary Fig. S9A, B). These results suggest that the redistribution of NKAα2 to the mitochondrial-enriched fraction may be involved in regulating the interaction between FH and Mul1, thereby contributing to the observed changes in FH protein levels and fumarate content.

### Fumarate ameliorates DCM in vitro and in vivo

To define the role of fumarate in DCM, we treated primary NRCMs or diabetic mice with fumarate. Fumarate treatment prevented HG/PA-induced decline in cellular viability, and suppressed LDH release (Supplementary Fig. S10A, B). We observed increased cell size with the exposure of HG/PA, while this increase was reversed in cells pretreated with fumarate (Supplementary Fig. S10C, D). Consistent with these results, mRNA levels of hypertrophic genes were induced by HG/PA but not upon fumarate treatment (Supplementary Fig. S10E). Interestingly, fumarate treatment had no effect on NKA activity in HG/PA-exposed NRCMs (Supplementary Fig. S10F). These results indicate that fumarate exerts a protective effect against diabetic cardiomyocyte injury and hypertrophy, acting as a downstream effector rather than an upstream regulator of NKAα2/NKA activity. In compliance with this, supplementation of fumarate counteracted the effects of NKAα2 deficiency on HG/PA-triggered injury, hypertrophy, and apoptosis in NRCMs (Supplementary Fig. S10H–N). These results showed that fumarate protects cardiac cells against HG/PA-induced hypertrophy.

**Fig. 7:**
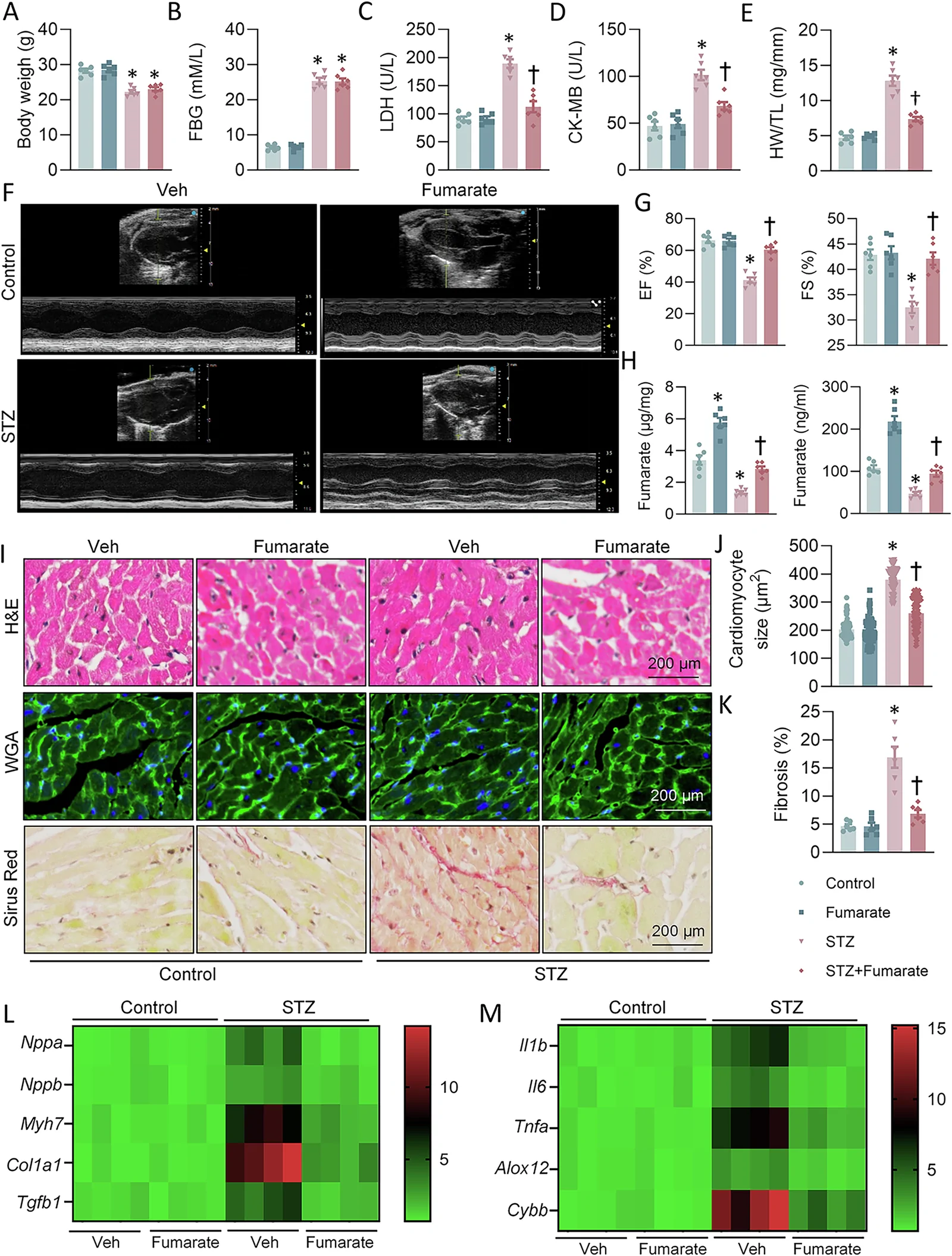
Fumarate ameliorates DCM. **A** Body weight. **B** FBG. **C** Serum LDH levels. **D** Serum CK-MB levels. **E** The heart weight–to–tibia length ratio (HW/TL). **F** Representative images of echocardiography in different groups. **G** EF and FS. **H** Fumarate levels in the mouse hearts (right) and serum (left). **I** Representative images of H&E staining, WGA staining, and Sirius Red staining in cardiac sections. **J** Quantitative analysis of cardiomyocyte size. **K** Quantitative analysis of cardiac fibrosis. **L** Relative mRNA levels of hypertrophic and fibrotic markers in the hearts, including *Nppa*, *Nppb*, *Myh7*, *Tgfb1*, and *Col1a1*. **M** Relative mRNA levels of *Il1b*, *Il6*, *Tnfa*, *Alox12*, and *Cybb*. Values were expressed as mean ± SD. The differences among the four groups were statistically analyzed by two-way ANOVA with Tukey’s multiple comparisons test. *P < 0.05 *vs*. Control. †*P* < 0.05 *vs*. STZ.

The body weight, fasting blood glucose (FBG), HbA1c, insulin levels, total cholesterol and triacylglycerols were comparable between diabetic mice and fumarate-treated diabetic mice (Fig. 7A, B). Fumarate treatment had no effect on NKA activity in the hearts from STZ-induced mice (Supplementary Fig. S10G). Treatment with fumarate significantly prevented STZ-increased circulating LDH and CKMB levels, and HW/TL ratio (Fig. 7C-E). Echocardiography revealed improved left ventricular systolic dysfunction in diabetic mice subjected to fumarate treatment (Supplementary Table S4). This improvement was evidenced by increases in ejection fraction and fractional shortening (Fig. 7F, G). Administration of fumarate led to a significant elevation of its levels in the serum and hearts of normal and STZ-induced diabetic mice (Fig. 7H). H&E and wheat germ agglutinin (WGA) staining showed enlarged cardiomyocyte cross-sectional areas in diabetic mice, but this increase was largely abrogated by fumarate treatment (Fig. 7I, J). Histological staining revealed significantly less myocardial fibrosis after treatment with fumarate, including extensive collagen deposition detected by Sirius red staining (Figs. 7I, 7K). Consistently, RT-qPCR analysis showed downregulations of hypertrophic and fibrotic markers, including *Nppa*, *Nppb*, *Myh7*, *Col1a1*, and *Tgfb1* (Fig. 7L). Also, the mRNA levels of pro-inflammatory factors, including *Il1b*, *Il6*, *Tnfa*, *Alox12*, and *Cybb,* were higher in the hearts from STZ-treated mice, but downregulated by administration of fumarate (Fig. 7M).

### Overexpression of FH reverses the benefits of NKAα2 in DCM

**Fig. 8:**
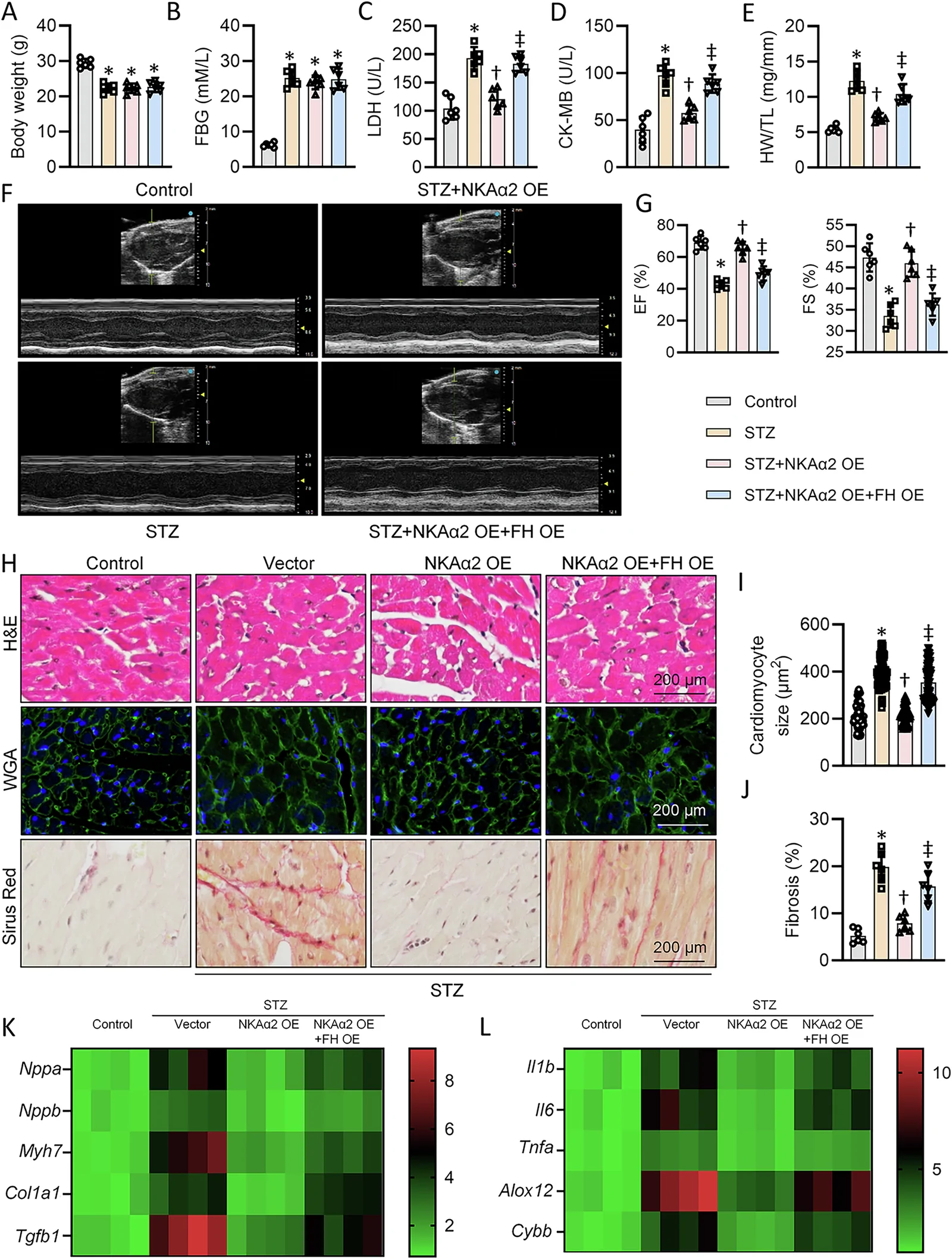
Overexpression of FH reverses the benefits of NKAα2 in DCM. **A** Body weight. **B** FBG. **C** Serum LDH levels. **D** Serum CK-MB levels. **E** The heart weight–to–tibia length ratio (HW/TL). **F** Representative images of echocardiography in different groups. **G** EF and FS. **H** Representative images of H&E staining, WGA staining, and Sirius Red staining in cardiac sections. **I** Quantitative analysis of cardiomyocyte size. **J** Quantitative analysis of cardiac fibrosis. **K** Relative mRNA levels of hypertrophic and fibrotic markers in the hearts, including *Nppa*, *Nppb*, *Myh7*, *Tgfb1*, and *Col1a1*. **L** Relative mRNA levels of *Il1b*, *Il6*, *Tnfa*, *Alox12*, and *Cybb*. Values were expressed as mean ± SD. The differences among the four groups were statistically analyzed by one-way ANOVA with Bonferroni’s post-hoc test. *P < 0.05 *vs*. Control. †*P* < 0.05 *vs*. STZ. ‡ *P* < 0.05 *vs*. STZ + NKAα2 overexpression (OE).

Eventually, we examined whether overexpression of FH reversed the cardioprotective effects of NKAα2 overexpression in the setting of DCM. Although FH overexpression did not affect body weight, FBG, heart rate, HbA1c, insulin levels, total cholesterol and triacylglycerols in DCM mice (Fig. 8A, B and Supplementary Table S5), it was sufficient to prevent the suppressive effects of NKAα2 overexpression on serum levels of LDH and CK-MB, as well as HW/TL (Fig. 8C–E). Cardiac-specific overexpression of NKAα2 improved STZ-induced cardiac dysfunction, which was attenuated by overexpression of FH (Fig. 8F, G and Supplementary Table S5). Diabetes led to cardiomyocyte enlargement in the mouse hearts, as observed in H&E and WGA staining, but this was notably reversed by NKAα2 overexpression. Likewise, overexpression of FH erased this phenomenon (Fig. 8H-I). Assessment of cardiac fibrosis in mice using Sirius red staining showed that FH overexpression blocked the favorable effects of NKAα2 on cardiac fibrosis (Fig. 8H, J). In accordance with this, overexpression of FH eliminated the anti-hypertrophic, anti-fibrotic, anti-inflammatory, and anti-oxidative effects of NKAα2 in the hearts from STZ-treated mice, as evidenced by measurement of mRNA levels of *Nppa*, *Nppb*, *Myh7*, *Col1a1*, *Tgfb1*, *Tnfa*, *Alox12*, and *Cybb* (Fig. 8K, L). These results indicate that suppression of FH activity is required for NKAα2 to exert its protective effects in the hearts from STZ-treated mice, thereby identifying the NKAα2/FH axis as a key regulatory pathway in diabetic cardiac remodeling.

## Discussion

This study unveils a pathogenic axis in the development of DCM, centered on the post-translational depletion of the NKAα2 in cardiomyocytes. We demonstrated that the upregulation of the depalmitoylating enzyme APT1 drove the removal of a palmitoyl moiety from NKAα2 at Cys462 under diabetic conditions. This depalmitoylation served as a molecular switch, redirecting NKAα2 from the plasma membrane to mitochondria, where it is ubiquitinated by the E3 ligase Mul1 and targeted for proteasomal degradation. The consequent loss of NKAα2 disrupted mitochondrial metabolic signaling, specifically by destabilizing fumarate hydratase (FH) and depleting the cardioprotective metabolite fumarate. Collectively, these findings identify the APT1/NKAα2/Mul1/FH pathway as a crucial mechanism linking metabolic stress to impaired cardiac function in diabetes, highlighting NKAα2 palmitoylation and mitochondrial fumarate metabolism as potential therapeutic targets for DCM.

DCM is a major complication of diabetes, characterized by structural and functional changes in the heart independent of coronary artery disease or hypertension [13, 53, 54]. While hyperglycemia and lipotoxicity are central drivers, the precise molecular mechanisms linking metabolic stress to cardiomyocyte dysfunction remain incompletely understood [6]. Dysregulation of NKA function has been implicated in various pathologies [55, 56], but its isoform-specific role in the context of diabetic metabolic stress is not well-defined. In this study, we found that NKAα2 was selectively downregulated at the protein level in the hearts from STZ-treated mice and HG/PA-exposed NRCMs, whereas other NKA α- and β-isoforms remained largely unaffected. This isoform-specific reduction in NKAα2 was accompanied by a significant decline in NKA enzymatic activity, indicating that loss of NKAα2 has functional consequences in the diabetic myocardium. Importantly, the unchanged mRNA levels of NKAα2 across whole hearts, isolated cardiac cell populations, and in vitro models consistently suggested a post-transcriptional regulatory mechanism, rather than transcriptional repression, underlying NKAα2 deficiency in diabetes. It is essential to validate these findings in human heart tissue from patients with diabetes and DCM.

Adeno-associated virus serotype 9 (AAV9) has emerged as a powerful tool for cardiac gene therapy due to its high tropism for cardiomyocytes and stable, long-term transgene expression [57–59], making it ideal for proof-of-concept studies in chronic models like DCM. We found that cardiac-specific overexpression of NKAα2 via AAV9 ameliorated multiple hallmarks of DCM. This demonstrates that the downregulation of NKAα2 is a causative event in the progression of diabetic heart injury. Moreover, NKAα2 restoration markedly suppressed inflammatory and oxidative stress–related gene expression, and reduced myocardial macrophage infiltration and cardiomyocyte apoptosis. These data suggest that loss of NKAα2 contributes to a pro-inflammatory and pro-oxidative myocardial milieu under diabetic conditions, while its restoration exerts broad anti-inflammatory and cytoprotective effects. The observed benefits of NKAα2 occurred without altering systemic metabolic parameters. This proves that correcting a primary cardiomyocyte defect can powerfully protect the heart, even in the ongoing presence of systemic diabetic stress. This decoupled cardiac pathology from global metabolic control and identified a direct cardiac target. Consistent with the in vivo findings, NKAα2 overexpression reduced cell death and hypertrophy. Conversely, siRNA-mediated knockdown of NKAα2 sensitized NRCMs to HG/PA-induced injury. This symmetrical gain-and-loss-of-function approach solidifies the conclusion that NKAα2 levels are a key determinant of cardiomyocyte resilience under metabolic stress. To definitively establish the essential role of endogenous cardiomyocyte NKAα2 in maintaining cardiac homeostasis under diabetic stress, the generation of cardiomyocyte-specific *Atp1a2* knockout (NKAα2-cKO) mice by crossing *Atp1a2*fl/fl mice with αMHC-Cre transgenic mice will be explored in future studies.

The molecular link between the diabetic metabolic milieu and the specific loss of a key membrane protein like NKAα2 has remained elusive. A critical missing piece is identifying the precise post-translational modification (PTM) that acts as the initial sensor and trigger for its downregulation. S-palmitoylation is a reversible lipid modification where a 16-carbon palmitate is attached to specific cysteine residues via a thioester bond [42]. This “greasy tag” critically anchors proteins to membranes (like the plasma membrane), regulates their trafficking, stability, and protein-protein interactions [60]. The dynamic balance of palmitoylation is maintained by palmitoyltransferases (DHHC family) and depalmitoylases like APT1 [61]. Dysregulation of this cycle is implicated in metabolic and cardiovascular diseases [62]. In the current study, we identify palmitoylation as a previously unrecognized regulatory mechanism governing NKAα2 stability and function under diabetic conditions. Using motif-based prediction and biochemical validation, we identified Cys462 as a critical palmitoylation site on NKAα2. Both in diabetic mouse hearts and in HG/PA-treated NRCMs, palmitoylated NKAα2 was markedly reduced, despite unchanged transcriptional levels. These findings position loss of NKAα2 palmitoylation as an early post-translational event that precedes and likely drives protein destabilization in DCM. APT1 is increasingly recognized as a mitochondria-associated S-deacylase that dynamically regulates mitochondrial protein palmitoylation [63]. The upregulation of APT1 in DCM suggests that metabolic stress activates a depalmitoylation program that selectively targets palmitoylated membrane proteins. Our data indicate that APT1-dependent depalmitoylation represents a critical switch that converts NKAα2 from a stable plasma membrane protein into a degradation-prone species, thereby linking metabolic stress to ion pump dysfunction. By predicting and validating Cys462 as the sole palmitoylation site on NKAα2, the study pinpoints the molecular “switch.” The finding that palmitoylation is decreased in the hearts from STZ-treated mice and HG/PA-induced cells, providing the direct link between metabolic stress and NKAα2 dysregulation. This depalmitoylation is the initiating event that explains the post-transcriptional protein loss. This finding provides direct causal evidence that palmitoylation at Cys462 is essential for maintaining NKAα2 stability and cardioprotective function.

Palmitoylation is tightly coupled to subcellular localization, particularly for membrane proteins [64]. Under physiological conditions, NKAα2 is predominantly localized at the plasma membrane, where it maintains Na⁺/K⁺ homeostasis and regulates excitation–contraction coupling. In line with this principle, our data suggest that HG/PA-induced depalmitoylation was associated with redistribution of NKAα2 from the plasma membrane to the mitochondrial-enriched fraction. This relocalization was abolished by APT1 knockdown or by the C462A mutation, establishing a mechanistic link between depalmitoylation and aberrant mitochondrial targeting of NKAα2. Its mislocalization to mitochondria under diabetic stress represents a pathological redistribution, exposing NKAα2 to mitochondrial quality-control machinery and predisposing it to degradation. This finding expands the emerging concept that mitochondria act not only as metabolic hubs but also as regulatory platforms for protein turnover in stressed cardiomyocytes.

Mul1 is a mitochondrial outer membrane E3 ligase implicated in mitochondrial dynamics, mitophagy, and metabolic stress responses [65]. In its aberrant mitochondrial location, NKAα2 encounters a different proteostatic environment. The identification of the mitochondrial E3 ligase Mul1 as its interaction partner is a masterstroke. This explains the compartment-specific degradation via the ubiquitin–proteasome system. The C462A mutation blocking ubiquitination proves that depalmitoylation is a prerequisite for this recognition and tagging. The interaction of Mul1 with NKAα2 provides a mechanistic link between mitochondrial stress signaling and membrane ion pump degradation. Collectively, these data suggest that APT1-mediated depalmitoylation may promote redistribution of NKAα2 to the mitochondrial-enriched fraction, facilitating its association with Mul1 and contributing to its ubiquitination and proteasomal degradation. The APT1–NKAα2–Mul1 axis uncovered here provides a unifying mechanism linking metabolic stress, post-translational modification, mitochondrial signaling, and ion pump dysfunction in DCM. Given Mul1’s role in mitochondrial dynamics, future studies should explore how NKAα2 degradation intersects with mitophagy and mitochondrial quality control in DCM.

Mitochondrial dysfunction is a cornerstone of DCM, characterized by impaired bioenergetics, excessive ROS production, and altered metabolite signaling [66]. Untargeted metabolomic profiling revealed that NKAα2 overexpression induces a distinct mitochondrial metabolic signature under glucolipotoxic conditions, with fumarate emerging as the most significantly altered metabolite. Machine learning–based prioritization further highlighted fumarate as a key node in NKAα2-mediated metabolic regulation, underscoring the biological relevance of this pathway. Fumarate, an intermediate of the TCA cycle, has transcended its classical metabolic role to become a recognized oncometabolite and signaling molecule [67]. In cardiovascular contexts, fumarate accumulation has been shown to exert protective effects since it can stabilize the transcription factor Nrf2 and bolster antioxidant defenses [49, 68]. Fumarate hydratase (FH), the enzyme that converts fumarate to malate [52], therefore acts as a critical gatekeeper of this signaling pool. In the hearts of diabetic mice, however, the role of fumarate signaling appears to be context-dependent. Our data demonstrate that diabetes increases FH protein abundance while concomitantly reducing fumarate levels, suggesting a maladaptive acceleration of fumarate consumption. This imbalance may deprive cardiomyocytes of fumarate-mediated cytoprotective signaling, thereby exacerbating oxidative stress, inflammation, and remodeling. Mechanistically, our data suggest that mitochondrially translocated NKAα2 competes with FH for binding to the mitochondrial E3 ligase Mul1, leading to dissociation of FH from Mul1, increased FH stability, and accelerated fumarate depletion when NKAα2 is deficient. Restoration of NKAα2 rebalances this interaction, restraining FH abundance and preserving fumarate signaling. This model provides a conceptual advance by identifying NKAα2 as an upstream regulator of mitochondrial enzyme turnover, rather than merely a passive metabolic consequence of diabetes. Functional studies in NRCMs and diabetic mice revealed that exogenous fumarate phenocopies the protective effects of NKAα2, suppressing hypertrophy, inflammation, oxidative stress, fibrosis, and contractile dysfunction, without altering systemic metabolic parameters. Importantly, fumarate supplementation rescued NRCM injury induced by NKAα2 deficiency, placing fumarate downstream of NKAα2 in the cardioprotective cascade. These findings reinforce the concept that fumarate functions as a bioactive metabolite, rather than a passive TCA intermediate, capable of modulating gene expression, redox homeostasis, and cellular stress responses in the diabetic heart. The fact that overexpressing FH completely abolished the benefits of NKAα2 overexpression proves that the protective effect of NKAα2 is mechanistically dependent on its ability to suppress FH activity/levels and thereby elevate fumarate. This solidifies the NKAα2/FH/fumarate axis as the core protective signaling cascade. Future studies should explore how fumarate modulates downstream signaling pathways (such as Nrf2 activation, protein succination, epigenetic regulation) and determine whether this axis operates in other forms of metabolic heart disease.

## Conclusion

Collectively, our findings identify NKAα2 as an important regulator of metabolic remodeling in DCM. Under diabetic stress, APT1-mediated depalmitoylation is associated with altered subcellular distribution and reduced stability of NKAα2. These changes are accompanied by enhanced Mul1-dependent regulation of FH, decreased fumarate levels, and aggravated cardiac injury and remodeling. Thus, the NKAα2/FH/fumarate axis may represent a mechanistic link between membrane-associated signaling and mitochondrial metabolism in STZ-induced DCM. However, the precise subcellular trafficking of NKAα2 and the compartment-specific role of APT1 require further investigation.

## Limitations

This study has several limitations. First, although our fractionation data showed increased NKAα2 in the mitochondrial-enriched fraction under diabetic stress, these findings do not definitively demonstrate true mitochondrial translocation. Contamination from mitochondria-associated membranes or other membrane compartments cannot be completely excluded. Therefore, our data should be interpreted more cautiously as reflecting a redistribution of NKAα2 into the mitochondrial-enriched fraction rather than unequivocal mitochondrial translocation. In addition, APT1 is not a canonical mitochondrial protein, and the present data do not support a definitive intramitochondrial localization of either APT1 or NKAα2, particularly within specific mitochondrial subcompartments. Therefore, the compartment-specific relationship between APT1-mediated depalmitoylation and NKAα2 redistribution under diabetic stress requires further investigation. In addition, our group has previously reported a protective role of another NKA isoform, NKAα1, in diabetes-induced endothelial dysfunction [22]. The present work focuses on NKAα2 in cardiomyocytes and identifies a distinct mechanism involving MUL1/FH-dependent fumarate metabolism in DCM. Future studies are needed to determine whether different NKA isoforms exert coordinated or complementary functions across multiple cardiac cell types during diabetes. It will also be important to clarify whether NKAα1 and NKAα2 engage shared upstream regulatory signals or distinct downstream pathways, and whether simultaneous targeting of these isoforms may provide broader protection against diabetic cardiovascular complications.

## Materials and methods

### Animals

All animal experiments were approved by the Laboratory Animal Welfare & Ethics Committee of Jiangnan University (Approve number: JN.No20221215m04831[559]) and were performed in accordance with the NIH Guide for the Care and Use of Laboratory Animals (8th edition, 2011). A total of 72 mice were used in this study, and six mice were included in each group. Six-week-old male C57BL/6J mice (Gempharmatech Co., Ltd.) were housed under a 12-h light/dark cycle with controlled temperature and humidity. In the animal experiments, each cohort contained 6 mice, and the assignment of animals to different cohorts was fully randomized. Furthermore, the investigators were aware of the treatment allocations throughout the experiment or when evaluating the outcomes. Following a one-week acclimatization period, a type 1 diabetes model was established. Mice received intraperitoneal injections of streptozotocin (STZ, 60 mg/kg in 0.1 M citrate buffer, pH 4.5) for five consecutive days. One week after the final injection, diabetes was confirmed by measuring fasting blood glucose (FBG) from the tail vein. Mice with FBG levels consistently exceeding 16.7 mM in three independent measurements were considered diabetic [4]. This cohort was then maintained for a further 12 weeks to develop DCM. To investigate the cardiac role of NKAα2, we performed gene manipulation using adeno-associated virus serotype 9 (AAV9) vectors. All vectors were driven by the cardiomyocyte-specific cTnT promoter for cardiac specificity. Six weeks after the initial STZ injection, mice received a single tail-vein injection. For gain-of-function studies, mice were administered AAV9 vectors carrying the full-length *Apt1a2* gene or a negative control vector (1 × 10¹¹ viral genomes per mouse). In addition, we examined whether overexpression of FH reversed the cardioprotective effects of NKAα2 overexpression in the setting of DCM. After STZ injection for 5 weeks, the mice received a single tail-vein injection of AAV9 vectors carrying the full-length *Fh* gene or a negative control vector (1 × 10¹¹ viral genomes per mouse). One week later, the mice received AAV9 vectors carrying the full-length *Apt1a2* gene (1 × 10¹¹ viral genomes per mouse). Six weeks later, the mice were subjected to echocardiography. To investigate the effects of fumarate on DCM, mice were administered intraperitoneal injections of fumarate (50 mg/kg, CAS No.: 624-49-7, Merck) every other day for the last 6 weeks during induction of diabetes [49, 69, 70]. FBG levels were measured and recorded at the end of the experiment. Body weight was measured at the endpoint. All glucose measurements in this study were based on fasting blood glucose (FBG). Mice were fasted overnight from 19:00 to 7:00. Blood was then collected from the tail vein, and glucose levels were measured using a glucose meter (Bbraun, Germany). At the conclusion of the study, mice were anesthetized with 1–2% isoflurane and euthanized by cervical dislocation after echocardiography.

### Serum biochemical analysis

Serum was isolated by centrifugation at 3000 rpm for 15 min and stored at −20 °C for subsequent analysis. We assessed a panel of metabolic and cardiac injury markers. General metabolic parameters, including fasting blood glucose (FBG), total cholesterol (TC), triglycerides (TG), and low-density lipoprotein (LDL), were measured. To evaluate cardiac damage, serum levels of creatine kinase-MB (CK-MB) and lactate dehydrogenase (LDH) were quantified using commercial kits (ERBA Diagnostics, Germany) according to the manufacturer’s protocols. All measurements were performed using a microplate reader.

### Western blotting and co-immunoprecipitation (Co-IP) analysis

For standard western blotting, protein lysates from heart tissue or cardiomyocytes were quantified after extraction with RIPA buffer. Equal amounts of protein were separated by 8–15% SDS-PAGE and transferred to PVDF membranes. Membranes were blocked for 2 h at room temperature with 2.5% skim milk. They were then probed with primary antibodies overnight at 4 °C. Primary antibodies were purchased, including NKAα1 (Cat No. 14418-1-AP, Proteintech), NKAα2 (orb385453, Biorbyt), NKAα3 (sc-71640, Santa Cruz), NKAβ1 (Cat No. 15192-1-AP, Proteintech), NKAβ2 (Cat No. 22338-1-AP, Proteintech), NKAβ3 (TA427011, Origene), NKAβ4 (orb579897, Biorbyt), β-actin (Cat No. 66009-1-Ig, Proteintech), APT1 (Cat No. 16055-1-AP, Proteintech), Pan-cadherin (Cat No. 31313-1-AP, Proteintech), COXIV (Cat No. 11242-1-AP, Proteintech), Ubiquitin (Cat No. 10201-2-AP, Proteintech), Flag (Cat No. 80801-2-RR, Proteintech), HA tag Polyclonal antibody (Cat No. 51064-2-AP, Proteintech), MUL1 (Cat No. 16133-1-AP, Proteintech), MARCH5 (Cat No. 12213-1-AP, Proteintech), Parkin (Cat No. 14060-1-AP, Proteintech), and FH (Cat No. 82508-1-RR, Proteintech). The following day, membranes were incubated with HRP-conjugated secondary antibodies (Anti-mouse HRP secondary antibody, RS0001, Immunoway, or Anti-rabbit HRP secondary antibody, RS0002, Immunoway) for 2 h at room temperature. Protein bands were visualized using enhanced chemiluminescence (ECL) reagents and imaged with an ECL detection system. This combined approach enabled the assessment of both protein-protein interactions and total protein expression levels. For co-IP experiments, proteins were extracted from cardiac tissue or cultured cells using RIPA buffer (Beyotime Biotechnology). A small aliquot of each lysate was saved as input. Protein G-Sepharose beads were pre-cleared with RIPA buffer prior to use. The remaining lysates were incubated overnight with the appropriate primary antibodies against NKAα2 (Cat No. 55179-1-AP, Proteintech) or FH (Cat No. 82508-1-RR, Proteintech) at 4 °C. Immune complexes were then precipitated by incubation with the pre-cleared beads for 6 h. Following precipitation, beads were washed extensively with PBS. Bound proteins were eluted by resuspension in loading buffer and heat denaturation for subsequent immunoblotting.

### Plasma membrane protein isolation and immunoblotting

To specifically analyze plasma membrane proteins, cells were surface-labeled using a membrane-impermeable biotinylation reagent. Intact cells were incubated with 1 mg/mL EZ-link Sulfo-NHS-SS-Biotin (Pierce) for one hour at 4 °C. Biotinylated proteins were subsequently isolated by streptavidin pull-down, as previously described [20]. Specifically, the abundance of the plasma membrane-localized NKAα1 subunit was normalized to pan-cadherin, a widely used membrane protein reference.

### Mitochondrial protein isolation

Mitochondrial proteins were extracted using a Mitochondria Isolation Kit (C3606, Beyotime) according to the manufacturer’s instructions. Following isolation, mitochondrial pellets were lysed in a buffer containing 1% (v/v) Triton X-100, 150 mM NaCl, 50 mM Tris-HCl (pH 7.4), and a cocktail of protease inhibitors. The lysates were clarified by centrifugation at 12,000 × *g* for 15 min at 4 °C, and the supernatant containing the soluble mitochondrial protein fraction was collected for subsequent analysis.

### Quantitative real-time polymerase chain reaction (qRT-PCR)

Total RNA was isolated from samples using TRIzol reagent according to the standard protocol. This involved phase separation with chloroform, RNA precipitation with isopropanol, and a final wash with ethanol. The purified RNA pellet was dissolved in RNase-free water. Complementary DNA (cDNA) was synthesized from 1 µg of total RNA using the SuperScript™ III First-Strand Synthesis SuperMix kit. Gene expression was then quantified by qPCR on a Real-Time System (Roche) with SYBR Green detection chemistry. Primer sequences are listed in Supplementary Tables S1 and 2. All target gene expression levels were normalized to the endogenous control GAPDH.

### Measurement of Na+/K+-ATPase (NKA) activity

NKA activity was measured using a modified spectrophotometric method [20, 22, 48]. Each sample homogenate was divided into two 50 µL aliquots for parallel reactions. One aliquot was incubated in a standard reaction buffer (50 µL) containing 200 mM Tris (pH 7.5), 30 mM MgCl₂, 200 mM NaCl, 60 mM KCl, and 10 mM EGTA. The second aliquot was incubated in an identical buffer supplemented with 2 mM ouabain to specifically inhibit NKA. Reactions were initiated by adding ATP to a final concentration of 1 mM. After a defined incubation period, reactions were terminated with 10 µL of 100% (w/v) trichloroacetic acid. Samples were kept on ice for one hour to precipitate proteins. The inorganic phosphate released in the supernatant was then quantified at 650 nm using a commercial spectrophotometric assay kit. The ouabain-sensitive NKA activity was calculated by subtracting the activity measured in the presence of ouabain from the total ATPase activity. This differential measurement isolates the specific contribution of NKA to total ATP hydrolysis.

### Immunofluorescence staining

To determine the subcellular localization of NKAα2, we performed dual immunofluorescence staining on cardiac tissue. Heart sections were permeabilized with 0.1% Triton X-100 for 15 min. They were then incubated overnight at 4 °C with primary antibodies against NKAα2 (rabbit, 55179-1-AP, Proteintech) and α-actinin (mouse, ab90421, Abcam). The following day, sections were incubated for one hour with Alexa Fluor® 594-conjugated goat anti-rabbit IgG and Alexa Fluor® 488-conjugated goat anti-mouse IgG. Nuclei were counterstained with DAPI. Cardiac sections were incubated with primary antibodies against F4/80 (Proteintech, 1:100) overnight, followed by fluorophore-conjugated secondary antibodies (1:200) for one hour at 37 °C. Nuclei were counterstained with DAPI. These sections were imaged using a Nikon microscope. For cells, the treated NRCMs were fixed in 4% paraformaldehyde for 30 min and blocked with 5% BSA for 1 h. The cells were stained overnight at 4 °C with an anti-NKAα2 antibody, followed by incubation with Alexa Fluor® 594-conjugated secondary antibody for one hour. All fluorescent images were captured using a Nikon 80i fluorescence microscope.

### Immunohistochemical analysis of NKAα2 in cardiac tissues

Formalin-fixed, paraffin-embedded heart tissue sections (4–5 µm) were deparaffinized in xylene and rehydrated through a graded ethanol series. Antigen retrieval was performed by heating sections in citrate buffer (pH 6.0) or Tris-EDTA buffer (pH 9.0) for 20 min in a pressure cooker. Endogenous peroxidase activity was quenched by incubation in 3% hydrogen peroxide for 15 minutes at room temperature. After blocking with 5% normal goat serum for 1 h, sections were incubated overnight at 4 °C with a primary antibody against NKAα2 (1:200). Following washing, sections were incubated with a horseradish peroxidase (HRP)-conjugated polymer secondary antibody for 1 h at room temperature. Antigen-antibody complexes were visualized using 3,3’-diaminobenzidine (DAB) as the chromogen, followed by counterstaining with Mayer’s hematoxylin. Stained sections were dehydrated, cleared in xylene, and mounted with a permanent medium. All sections were examined and imaged under a light microscope.

### Echocardiography

Cardiac function was assessed using transthoracic echocardiography. Prior to imaging, mice were shaved to remove chest hair and anesthetized with inhaled isoflurane. They were then positioned on a thermostatically controlled heating plate in a supine position with limbs gently extended. A 30 MHz ultrasound probe (Visualsonics, Toronto, Canada) was used to obtain two-dimensional short-axis views of the left ventricle. Ultrasound gel was applied to ensure optimal acoustic coupling. Left ventricular ejection fraction (LVEF), fractional shortening (LVFS), and end-diastolic volume (LVEDV) were derived from M-mode recordings. For each mouse, at least six measurements were performed. The final values represent the average of three consecutive, stable cardiac cycles.

### Histological analysis of cardiac tissues

Heart tissues were fixed in paraformaldehyde for 48 h, dehydrated, and paraffin-embedded. Sections (5.0 µm) were cut and deparaffinized in xylene, followed by rehydration through a graded ethanol series. We performed several standard histological stains to assess structural pathology. Myocardial disorganization was evaluated using Hematoxylin and Eosin (H&E; Solarbio G1120). Cardiac fibrosis was quantified using Sirius Red staining kit (Solarbio G1472). To measure cardiomyocyte cross-sectional area, paraffin sections were stained with fluorescein-conjugated Wheat Germ Agglutinin (WGA; Solarbio CA1610). Brightfield and fluorescence images were captured using a Nikon 80i microscope. Apoptotic cell death was detected in situ using a One-Step TUNEL assay kit (Beyotime), following the manufacturer’s protocol.

### Enzyme-linked immunosorbent assay (ELISA)

Systemic cytokine levels were profiled by ELISA. Serum was collected from mice. The concentrations of pro-inflammatory cytokines, interleukin-1β (IL-1β), interleukin-6 (IL-6), and tumor necrosis factor-α (TNF-α) were quantified using commercially available ELISA kits (Boster Bio) [71]. Serum levels of insulin were also measured by ELISA (EZRMI, Merck Millipore, Danvers, MA). All assays were performed strictly in accordance with the manufacturer’s instructions and established protocols.

### Cell culture

Primary cardiac cells were isolated and cultured using established protocols [4, 11, 12]. Primary NRCMs were obtained from 1–3-day-old Sprague-Dawley rats. Mouse cardiac endothelial cells and fibroblasts were prepared as previously described [4, 11]. We first quantified *NKAα2* mRNA levels in cardiac fibroblasts and endothelial cells from control and diabetic mice. For in vitro injury modeling, NRCMs were plated in six-well plates (2.5 × 10⁴ cells/well). Cells were maintained in low-glucose DMEM (1 g/L) supplemented with 10% FBS, 50 μg/ml gentamicin, and 10 μM Ara-C to suppress fibroblast proliferation. Following a 12-h synchronization period, a diabetic milieu was simulated by treating cells with high glucose (HG, 33.3 mM) and palmitic acid (PA, 100 μM) for 72 h. HG medium was prepared by supplementing the basal culture medium with D-glucose to a final concentration of 33.3 mM. PA was first conjugated to free fatty acids (FFAs)-free BSA to improve solubility and cellular delivery, and then added to the culture medium to a final concentration of 100 μM. Mannitol at the same concentration was used as osmolarity control (OC; 5.5 mmol/L glucose and 27.8 mmol/L mannitol). PA was conjugated to free fatty acid (FFA)-free BSA before use [72]. Briefly, PA was dissolved in 0.1 M sodium hydroxide solution (NaOH) by heating at 70 °C until fully solubilized, and then mixed with prewarmed 10% FFA-free BSA solution to generate a PA–BSA stock solution, which was further diluted with culture medium to a final working concentration of 100 μM before cell treatment. Genetic manipulations were performed to alter NKAα2 expression. For knockdown, NRCMs were transfected with 10 nM of either NKAα2-specific siRNA (SR510710, OriGene), APT1-specific siRNA (SR507025, OriGene) or scramble control siRNA using Lipo3000™ reagent. For overexpression, cells were transduced with NKAα2 lentivirus vectors or control lentivirus vector (1 × 10^6^ TU/mL) for 24 h. All transfected or transduced cells subsequently underwent the 72-h HG/PA challenge. NRCMs were pretreated with fumarate (10 μM, CAS No.: 624-49-7, Merk) [73] for 6 h, and then underwent the 72-h HG/PA challenge. Following these treatments, we assessed key pathological endpoints, including cardiomyocyte hypertrophy and injury.

*Cell viability* Primary NRCMs were seeded at a density of 5000 cells per well in 96-well plates and allowed to adhere overnight in complete growth medium. Cells were then synchronized in serum-free medium for 12 h prior to the application of HG/PA treatment. Following the HG/PA treatment for 72 h, cell viability was quantified using a Cell Counting Kit-8 (CCK-8; Dojindo, Japan). Briefly, 10 µL of the CCK-8 reagent was added directly to each well containing 100 µL of culture medium. The plate was incubated at 37 °C for 2 h in a humidified incubator. The absorbance was measured at 450 nm using a microplate reader. Relative cell viability was calculated as: Relative viability = (OD_sample − OD_blank)/(mean OD_control − signal_blank). Data from independent experiments were then averaged for statistical analysis. This assay reflects overall cellular metabolic activity rather than direct cell number or percentage cell death.

### Plasmid construction and transfection

Expression plasmids were generated for protein-protein interaction studies and domain mapping. The full-length cDNA of human *Mul1* and a fragment of *ATP1A2* (NKAα2) encoding its soluble, non-transmembrane region were used as templates. The full-length *Mul1* coding sequence was cloned into pcDNA3.1-HA vectors, resulting in N-terminally tagged HA-USP22. Similarly, the *NKAα2* fragment was inserted into pcDNA3.1-Flag vectors to generate Flag-NKAα2. The constructed expression plasmids were introduced into HEK-293 cells via transient transfection for subsequent protein-protein interaction assays. Cells were seeded in appropriate culture dishes and grown to 60-70% confluence. Transfection was performed using the Lipofectamine 3000 reagent (Thermo Fisher Scientific) according to the manufacturer’s protocol. For each transfection, a total of 1 µg of plasmid DNA was mixed with the recommended volumes of P3000™ Enhancer and lipofectamine reagent in Opti-MEM reduced serum medium. The DNA-lipid complexes were incubated for 15 min at room temperature and then added dropwise to the cells. Following an incubation period of 48 h, cells were harvested for further analysis, such as co-immunoprecipitation or immunoblotting.

### Molecular docking

The protein models of NKAα2 and Mul1 used for docking were retrieved from the AlphaFold Protein Structure Database (https://alphafold.ebi.ac.uk/). Protein–protein molecular docking was performed using the HDOCK server (http://hdock.phys.hust.edu.cn/) [74]. Prior to docking, protein structures were preprocessed in PyMOL 2.4, which included removal of water molecules and extraneous ligands, as well as the addition of hydrogen atoms. The docking score served as the primary criterion for evaluating the docking results, and the top 10 docking poses were generated. The model with the highest docking score was selected as the optimal docking conformation. Finally, the docking results were visualized using PyMOL 2.4, allowing for an intuitive assessment of the predicted protein–protein interaction interface.

### Molecular dynamics (MD) simulations

In this study, we performed all-atom MD simulations on the protein-protein complex (NKAα2 and Mul1) obtained from molecular docking using GROMACS 2022.4 [75, 76]. The system was parameterized with the Amber14SB force field. A cubic solvation box was applied, and the minimum distance from the system boundary to the complex was set to 1 nm. The TIP3P water model was used, and an appropriate number of sodium and chloride ions were added to neutralize the system charge. The system was energy-minimized using the steepest descent method. Temperature and pressure were equilibrated under the NVT and NPT ensembles, respectively, maintaining the system at 300 K and 101.325 kPa. Following equilibration, a 100 ns production MD simulation was performed at 300 K for each system, generating a total of 10,000 trajectory frames. Using the resulting trajectories, we analyzed key structural and dynamic parameters, including the root-mean-square deviation (RMSD), root-mean-square fluctuation (RMSF), radius of gyration (Rg), and the total number of intermolecular hydrogen bonds between the proteins.

### EdU staining

Primary NRCMs were seeded in culture plates and allowed to adhere. Following serum starvation for synchronization, cells were treated according to the experimental design. To assess cell proliferation, a 5-ethynyl-2’-deoxyuridine (EdU) assay kit (Click-iT™ Plus EdU Cell Proliferation Kit, Alexa Fluor™ 488 dye, C10637, Thermo Fisher Scientific) was employed. Cells were incubated with 10 µM EdU in complete medium for 6 h at 37 °C, allowing for incorporation into newly synthesized DNA. After labeling, cells were fixed with 4% paraformaldehyde for 15 min and permeabilized with 0.5% Triton X-100 in PBS for 20 minutes. The Click-iT reaction cocktail, containing fluorescent azide, was then applied for 30 min in the dark to label EdU-positive nuclei. Cell nuclei were counterstained with Hoechst 33342 for 10 min. Fluorescent images were captured using a fluorescence microscope or confocal system. The EdU-positive (proliferating) cells and total nuclei (Hoechst-positive) were counted in six random fields per replicate. The proliferation rate was expressed as the percentage of EdU-positive nuclei relative to the total number of nuclei.

### Lactate dehydrogenase (LDH) release in cells

Cytotoxicity was assessed by measuring LDH release in cell culture supernatants. Following experimental treatments, lysis buffer was added to the cells. The supernatant was then collected after centrifugation. LDH activity was quantified according to the manufacturer’s instructions for the commercial assay kit (P0392S, Biyuntian, China).

### Caspase 3 activity

Caspase3 enzymatic activity in NRCMs was detected using commercial activity kits (Applygen, Beijing, China), as previously described [77]. Cells were lysed in lysis buffer at 4 °C for 30 min. The lysate was centrifuged at 12,000 × *g* for 5 min to collect the supernatant. Protein concentration in the supernatant was determined by the Bradford method. A 96-well plate was used for subsequent activity assays. Each well received 50 μg of cell lysate mixed with the corresponding reaction reagent. The plate was incubated at 37 °C for 2 h in the dark. Caspase3 activity was calculated based on absorbance measured at 405 nm.

### Phalloidin fluorescence analysis

To visualize cellular size, cells were grown to 50% confluence. They were washed three times with PBS and fixed with 4% paraformaldehyde for 10 min at room temperature. Following fixation, cells were permeabilized with 0.5% Triton X-100 in PBS for 5 min and washed again. Cells were then incubated with TRITC-conjugated phalloidin (100 nM, diluted in PBS optionally containing 1% BSA) in the dark for 30 min. After thorough washing, nuclei were counterstained with DAPI for 30 s. Fluorescence images were captured using a fluorescence microscope to assess cytoskeletal morphology.

### Detection of NKAα2 protein palmitoylation

NKAα2 palmitoylation was analyzed using an immunoprecipitation-coupled acyl-biotin exchange (IP-ABE) assay, adapted from a published protocol [78]. Briefly, proteins were extracted in lysis buffer containing protease inhibitors and 50 mM N-ethylmaleimide (NEM) to block free thiols. NKAα2 was immunoprecipitated from lysates using an anti-NKAα2 antibody and protein A/G magnetic beads (Millipore). The bead-bound complexes were then divided into two equal aliquots. One aliquot was incubated in 0.5 mL of hydroxylamine (HAM) buffer (0.7 M HAM, 50 mM HEPES, pH 7.0) to cleave thioester-linked palmitate. The control aliquot was incubated in 0.5 mL of HAM-free buffer (50 mM HEPES, pH 7.0). Both sets of samples were rotated at room temperature for 50 min. Newly exposed thiol groups were labeled by incubating samples with 0.5 mM Biotin-BMCC in HEPES buffer for 50 minutes at 4 °C with rotation. Beads were washed thoroughly, and proteins were eluted in SDS sample buffer by boiling. Eluted proteins were separated by SDS-PAGE and transferred to PVDF membranes. For detection, membranes were probed with HRP-conjugated streptavidin to visualize palmitoylated NKAα2 proteins. To assess total NKAα2 levels, parallel membranes were probed with an anti-NKAα2 primary antibody, followed by an HRP-conjugated secondary antibody.

### Untargeted metabolomics

Samples were thawed at room temperature [79–81]. For metabolite extraction, 50 µL of each sample ((50 mg)) was combined with 200 µL of cold methanol:acetonitrile (2:1 v/v) containing L-2-chlorophenylalanine (2 µg/mL) as an internal standard. The mixture was vortexed for one minute and sonicated for 10 minutes in an ice-water bath. Following a 30-minute incubation at −40 °C, samples were centrifuged at 13,000 rpm for 10 minutes at 4 °C. A 150 µL aliquot of the supernatant was filtered through a 0.22 µm membrane and transferred to an LC vial for storage at −80 °C until analysis. A pooled quality control (QC) sample was generated by combining equal volumes of all extracted supernatants. Untargeted metabolomic profiling was performed using an ACQUITY UPLC I-Class system coupled to a Q Exactive HF mass spectrometer (Thermo Scientific). Chromatographic separation was achieved on an ACQUITY UPLC HSS T3 column (100 mm × 2.1 mm, 1.8 µm) maintained at 45 °C. The mobile phase consisted of (A) water with 0.1% formic acid and (B) acetonitrile, delivered at a flow rate of 0.35 mL/min. The injection volume was 5 µL. Mass spectrometry data were acquired in both positive and negative ionization modes. Raw data were processed using Progenesis QI software (version 2.3) for peak alignment, picking, and normalization. Processing parameters included a 5 ppm mass tolerance for precursor ions and a 10 ppm tolerance for fragments. Metabolites were identified by matching accurate mass, MS/MS spectra, and isotopic patterns against the HMDB, LipidMaps, METLIN, and in-house databases. For statistical analysis, features with >50% missing values within any group were removed. Missing values were imputed with half of the minimum positive value. Differential metabolites (*p* < 0.05) were selected and annotated using the KEGG database. Multivariate statistical analysis was performed using MetaboAnalyst 4.0. Univariate statistical differences of the metabolites between the two groups were analyzed using Student’s *t* test. The mass spectrometry metabolomics data generated in this study have been deposited in the National Genomics Data Center under accession number OMIX014226. Two ensemble machine learning algorithms, Random Forest and AdaBoost, were employed for classification and feature selection based on metabolomics data from the HG/PA group and HG/PA + NKAα2OE group. Random Forest (RF), introduced by Breiman, operates by constructing multiple decision trees during training. It utilizes bootstrap aggregation (bagging) and random feature selection to enhance model generalization and mitigate overfitting. Each tree provides a classification vote, and the final output is determined by majority voting. The algorithm also estimates variable importance, where a higher importance score indicates a greater contribution of the corresponding variable (metabolite) to classification accuracy. *AdaBoost* is proposed by Freund and Schapire. AdaBoost is an adaptive boosting algorithm. It works by iteratively training a series of weak classifiers (e.g., decision stumps) on the training data. In each iteration, increasing the weight of misclassified samples and decreases the weight of correctly classified ones. This forces subsequent classifiers to focus more on difficult cases. The final prediction is a weighted sum of the weak classifiers’ outputs. This approach confers strong generalization ability and feature selectivity.

### Measurement of fumarate

The levels of fumarate were assessed in cardiac tissue or cell lysates. Samples were prepared according to the specifications of a commercial assay kit (MAK060, Sigma-Aldrich) [82]. All assays were performed strictly following the manufacturer’s instructions.

### Statistical analysis

Each group had a minimum sample size of four, ensuring sufficient power to detect the prespecified effect sizes. Cellular and molecular experiments were independently repeated at least four times. Animal experiments included a minimum of six mice per group for independent biological replication. Subjects were randomly assigned to either the control or experimental group. The investigators were not blinded to group allocation during the experiment or outcome assessment. Furthermore, for all figures, the statistical tests employed were appropriate, and the data satisfied the required assumptions. The variances estimated for the groups being compared were similar. All data are presented as the mean ± standard deviation (SD). We generated a graphical abstract using AI tools independently, rather than outsourcing to a third-party design platform. Statistical analyses were performed using GraphPad Prism 9.0. Differences between two groups were assessed using an unpaired Student’s *t* test. For comparisons across multiple groups, one-way analysis of variance (ANOVA) was applied, followed by Bonferroni’s post-hoc test; two-way ANOVA was applied, followed by Tukey’s multiple comparisons test. A *p* value of less than 0.05 was considered statistically significant.

## Supplementary information

Supplmentary data

Original Blots

## Data Availability

All data supporting the findings of this study are included in the article and its supplementary information files
