# Supplementary material for: Palmitoylation-driven upregulation of NKAα2 improves diabetic cardiomyopathy: a role for the fumarate hydratase/fumarate metabolic pathway
Source: Cell Death Discov. 2026 Jul 29;12:425. doi: 10.1038/s41420-026-03275-9 (PMC13696732; doi:10.1038/s41420-026-03275-9)
Supplement: Supplementary file 1 [file 41420_2026_3275_MOESM2_ESM.pdf]

Full unedited gel for Figure 1A

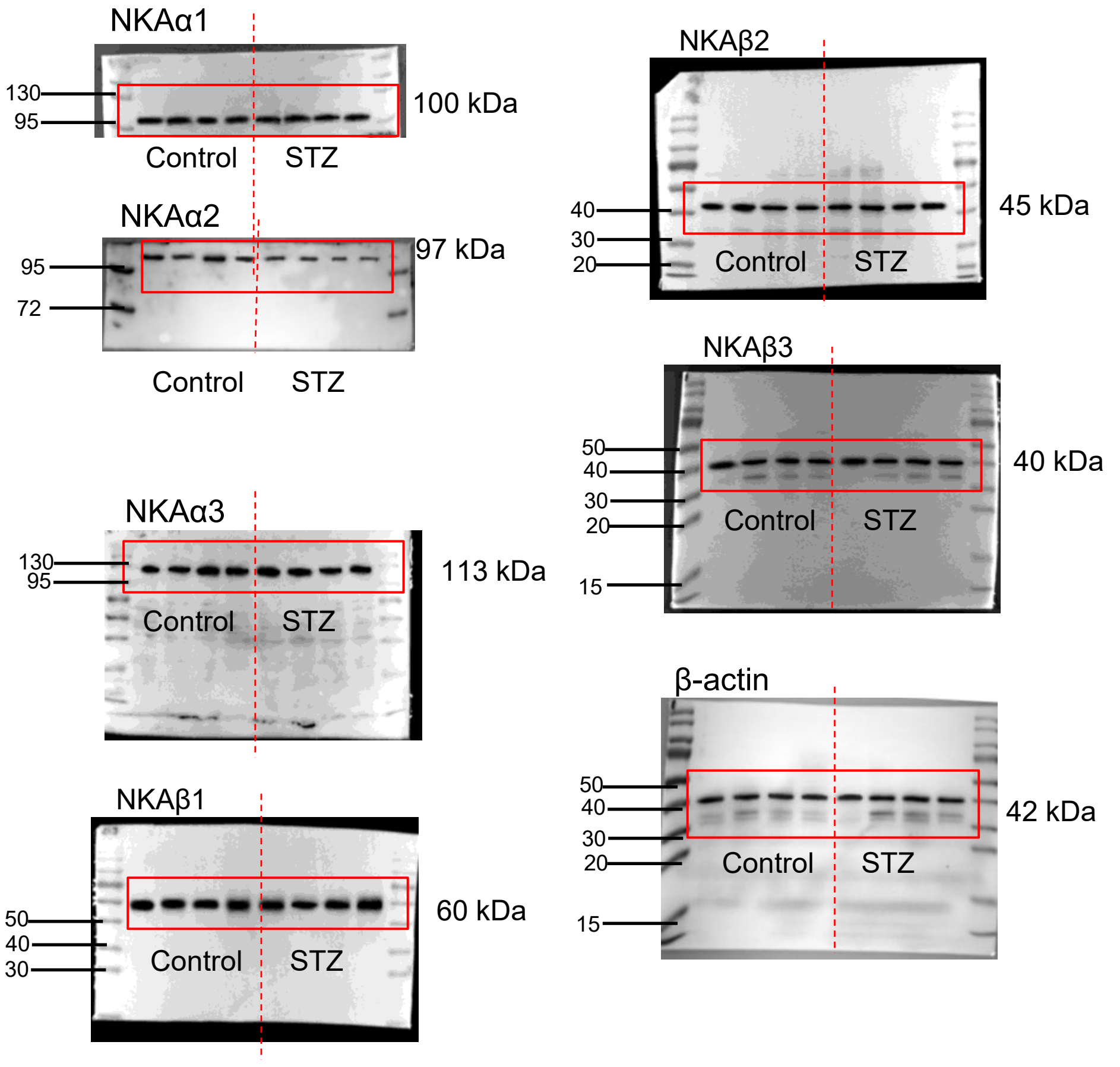

Full uncropped immunoblots in **Figure 1A**. The red rectangle indicates the portion of the blot displayed in the manuscript. n=4.

Full unedited gel for Figure 1F

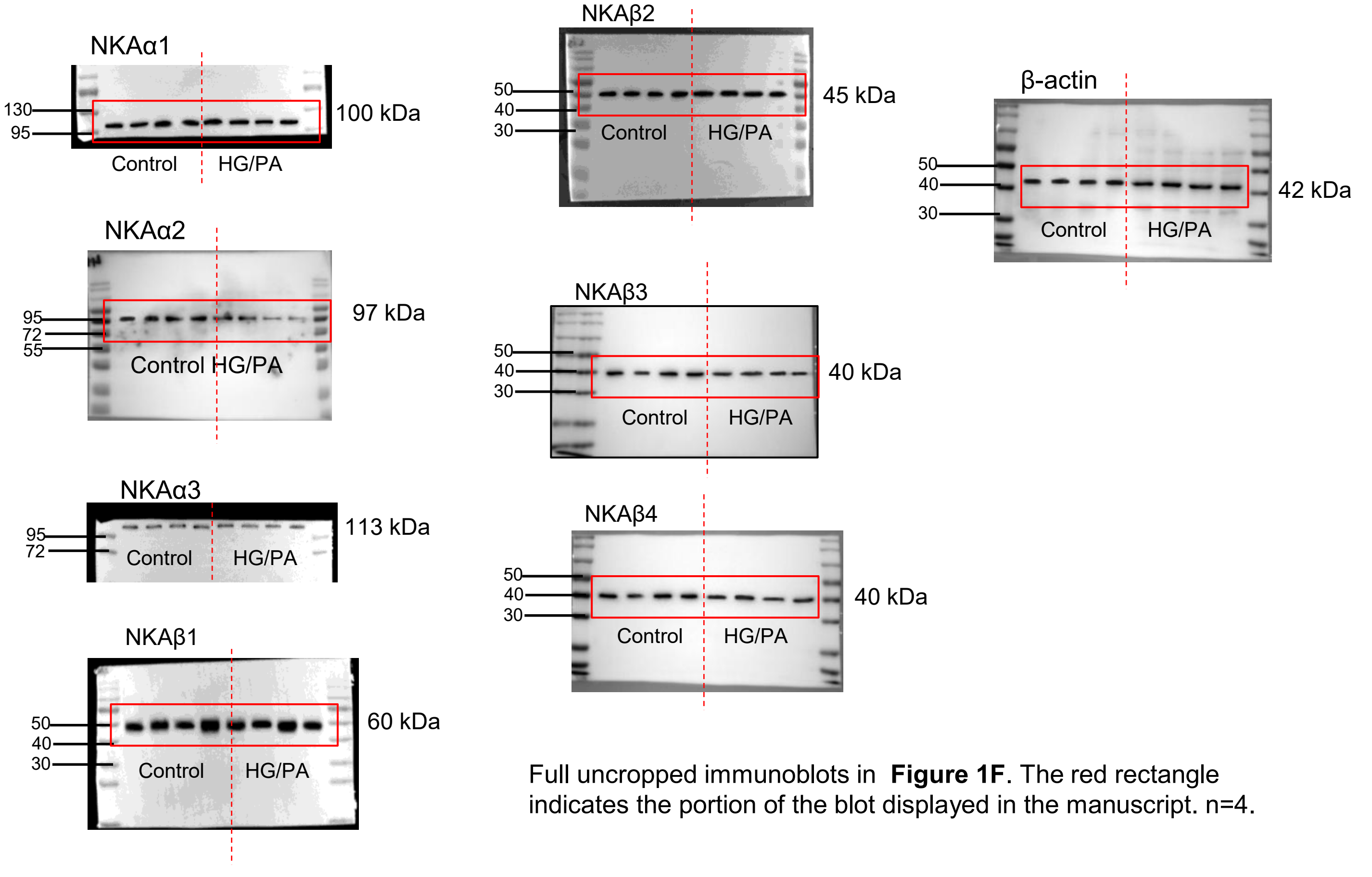

Full uncropped immunoblots in **Figure 1F**. The red rectangle indicates the portion of the blot displayed in the manuscript. n=4.

Full unedited gel for Figure 4A

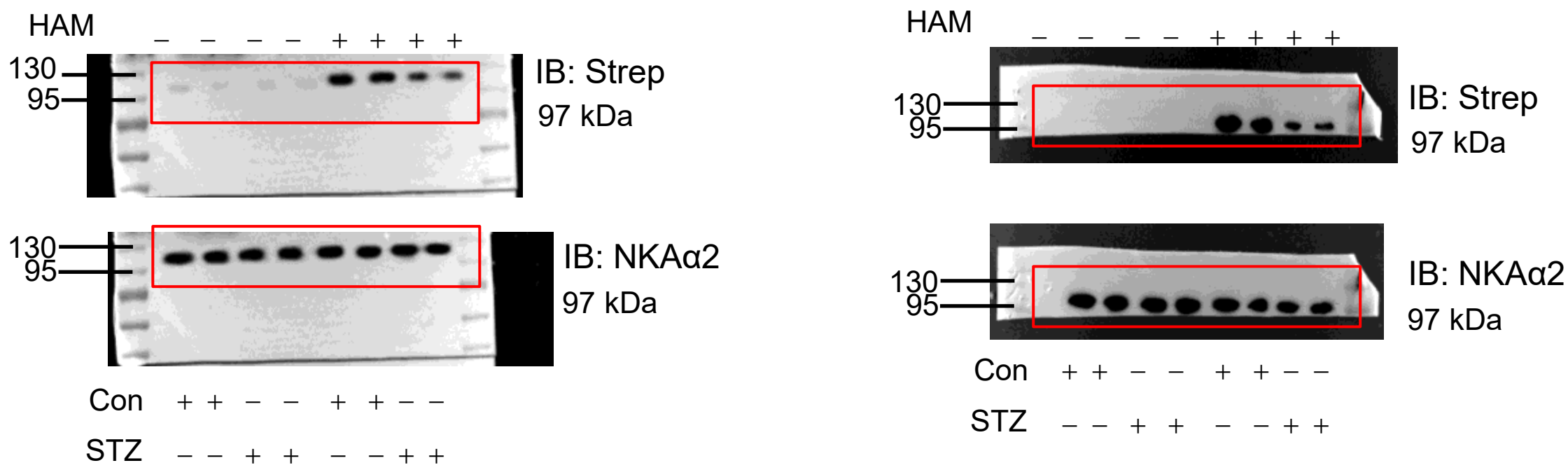

Full uncropped immunoblots in **Figure 4A**. The red rectangle indicates the portion of the blot displayed in the manuscript. n=4.

Full unedited gel for Figure 4B

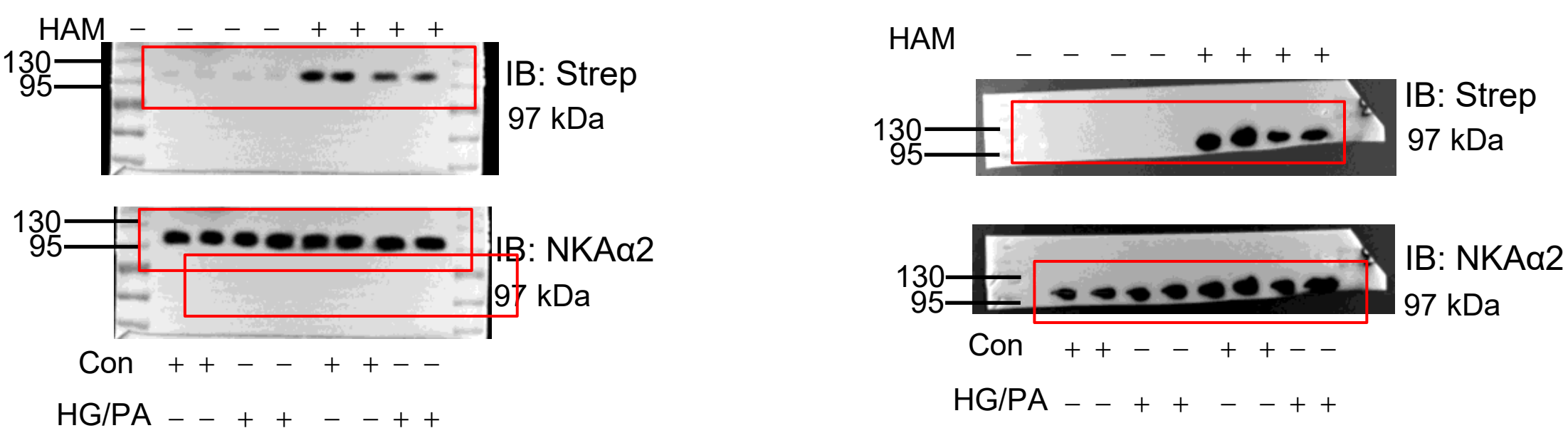

Full uncropped immunoblots in **Figure 4B**. The red rectangle indicates the portion of the blot displayed in the manuscript. n=4.

Full unedited gel for Figure 4D

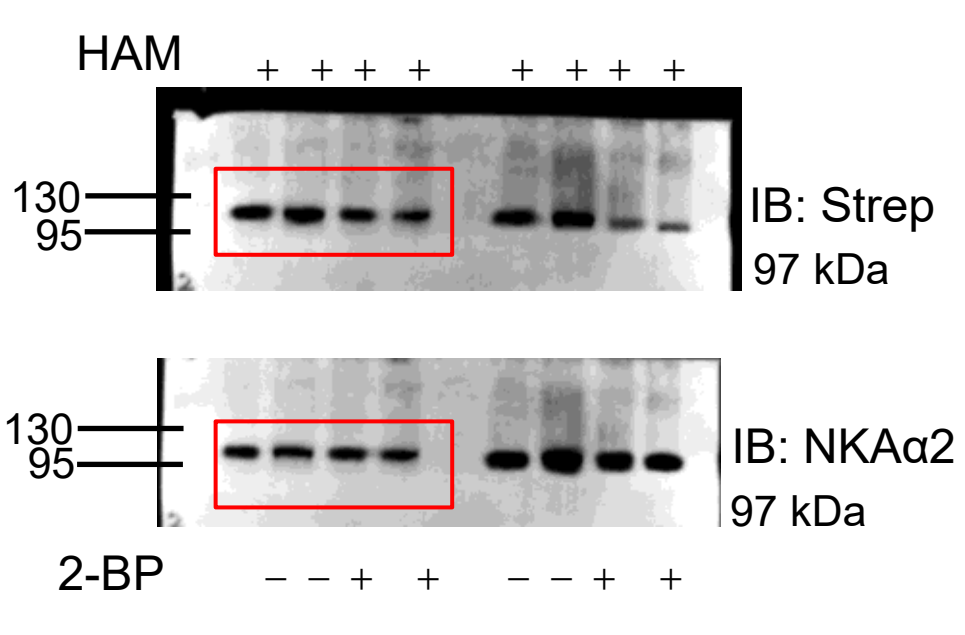

Full uncropped immunoblots in **Figure 4D**. The red rectangle indicates the portion of the blot displayed in the manuscript. n=4.

Full unedited gel for Figure 4E

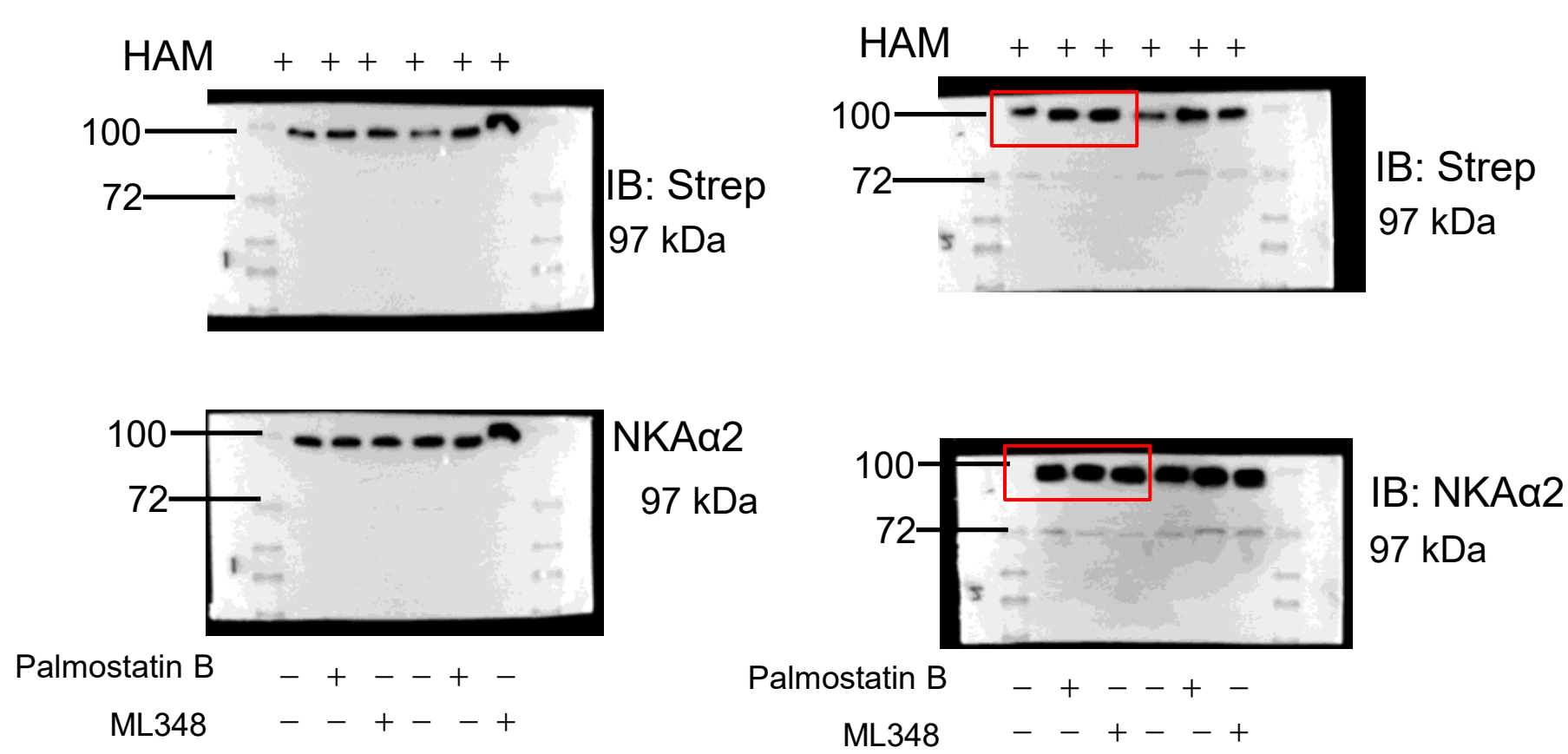

Full uncropped immunoblots in **Figure 4E**. The red rectangle indicates the portion of the blot displayed in the manuscript. n=4.

Full unedited gel for Figure 4H

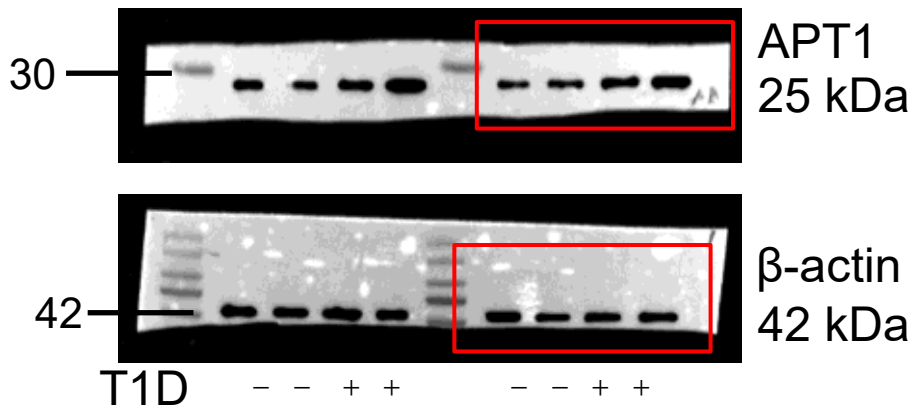

Full unedited gel for Figure 4I

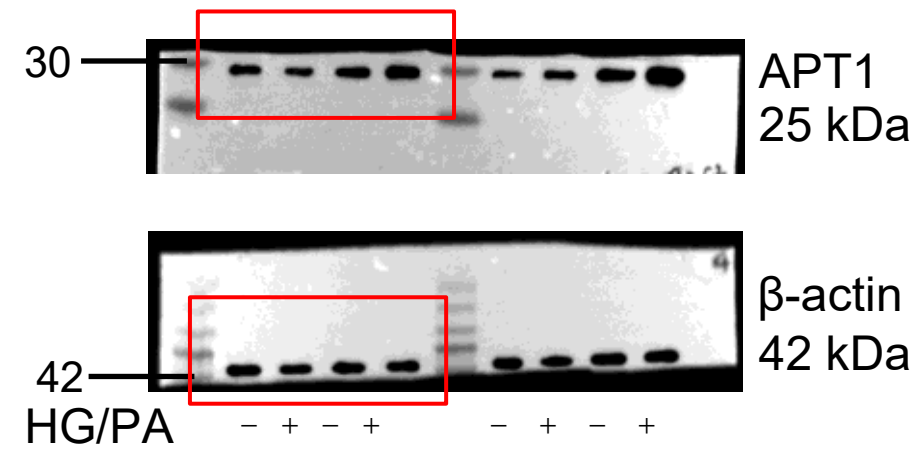

Full uncropped immunoblots in **Figure 4H-I**. The red rectangle indicates the portion of the blot displayed in the manuscript. n=4.

Full unedited gel for Figure 4K

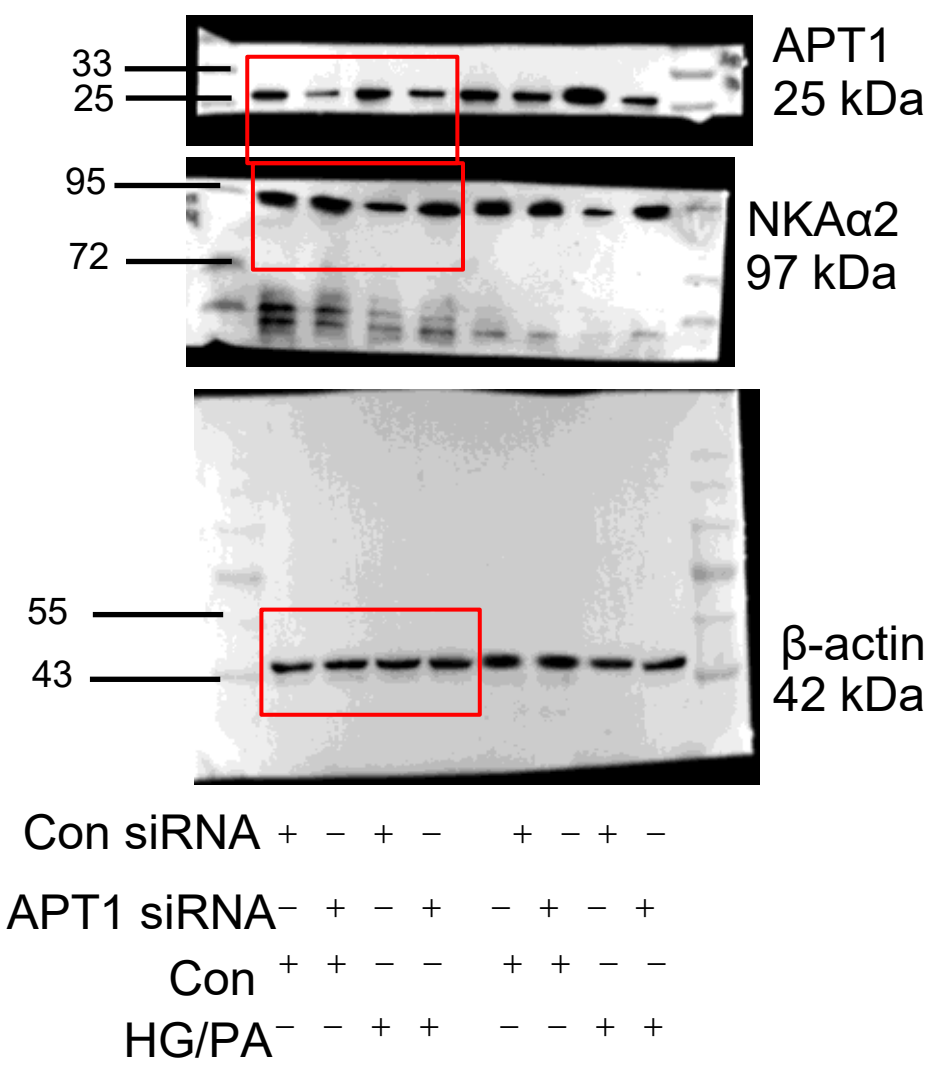

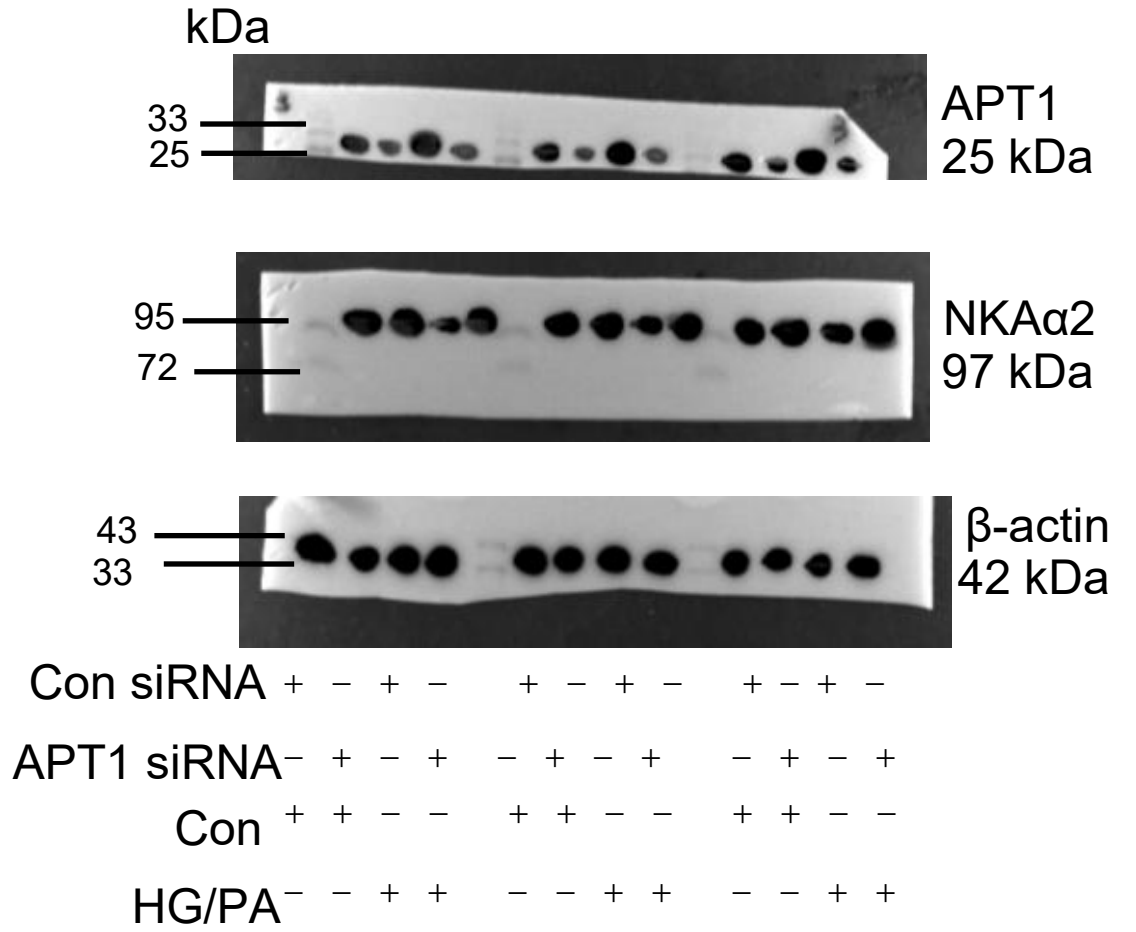

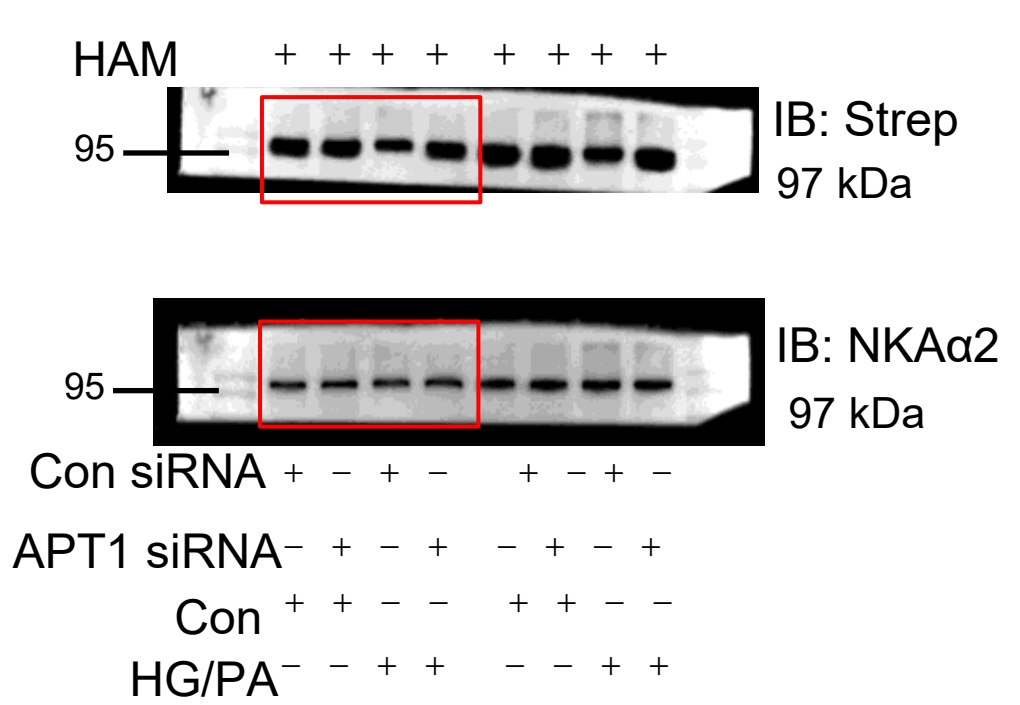

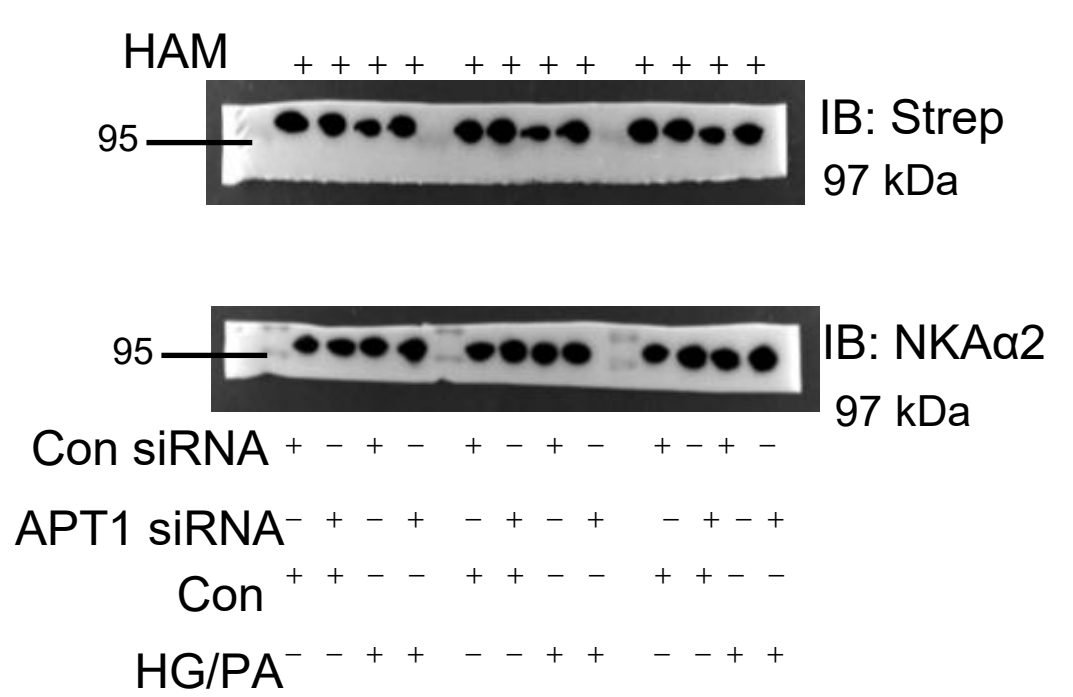

Full uncropped immunoblots in **Figure 4K**. The red rectangle indicates the portion of the blot displayed in the manuscript. n=5.

Full unedited gel for Figure 5A

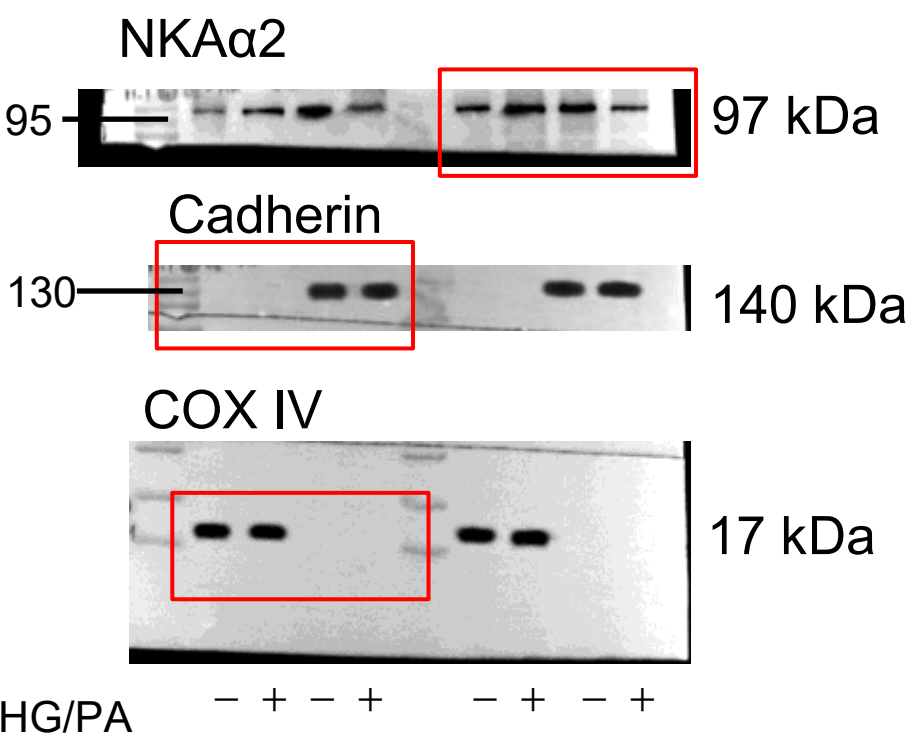

Full uncropped immunoblots in **Figure 5A**. The red rectangle indicates the portion of the blot displayed in the manuscript. n=4.

Full unedited gel for Figure 5B

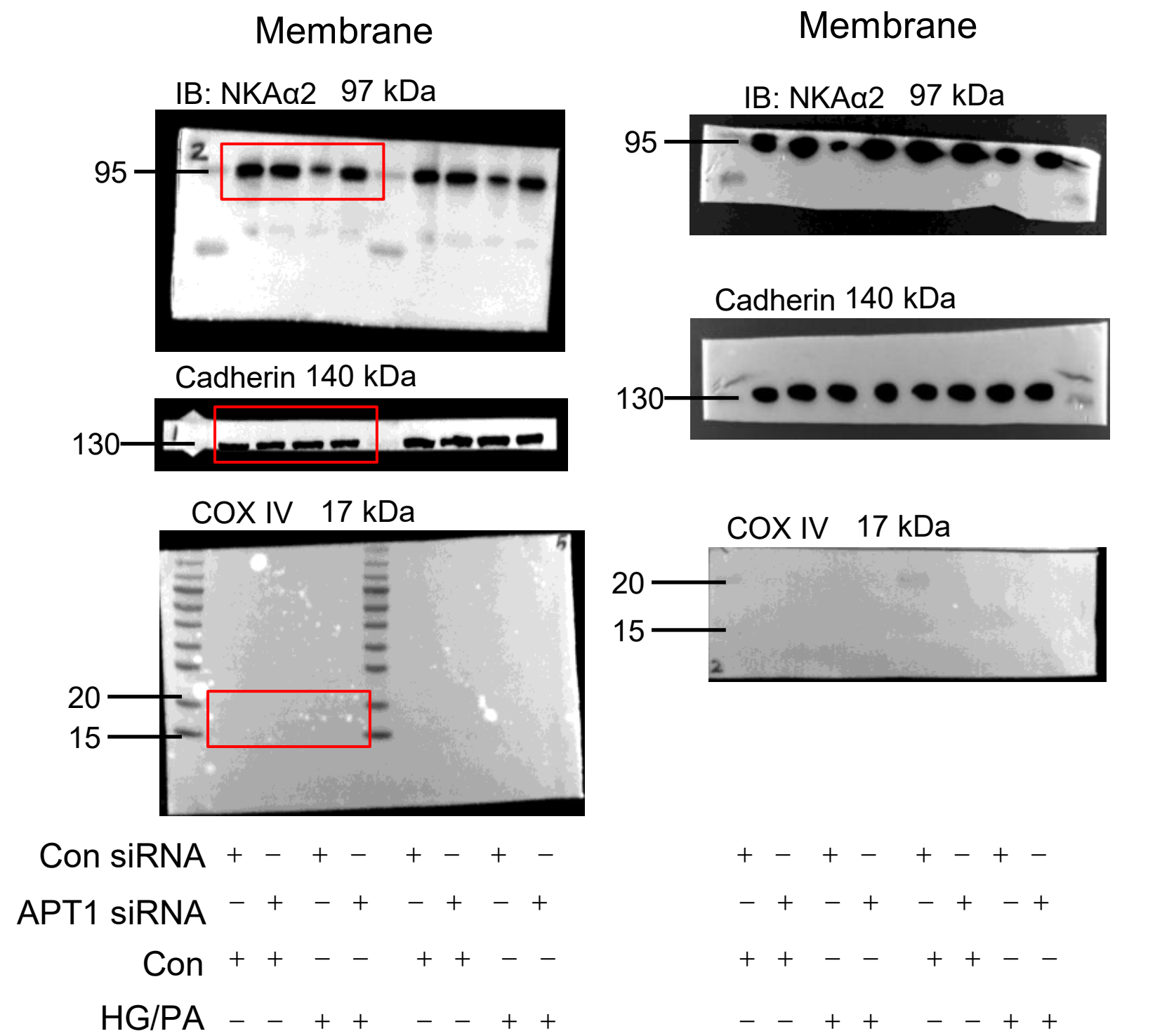

Full uncropped immunoblots in **Figure 5B**. The red rectangle indicates the portion of the blot displayed in the manuscript. n=4.

Full unedited gel for Figure 5C

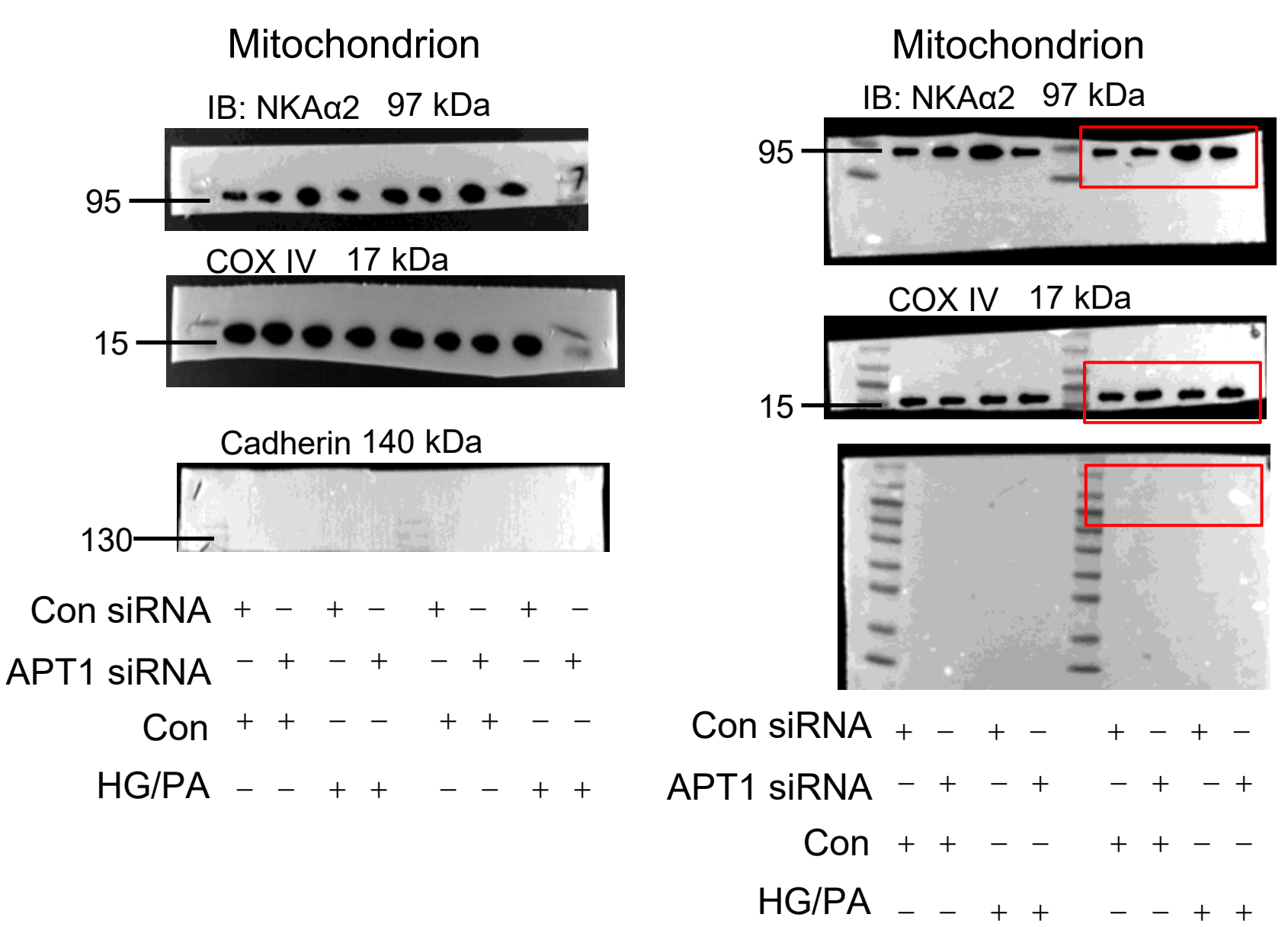

Full uncropped immunoblots in **Figure 5C**. The red rectangle indicates the portion of the blot displayed in the manuscript. n=4.

Full unedited gel for Figure 5E-G

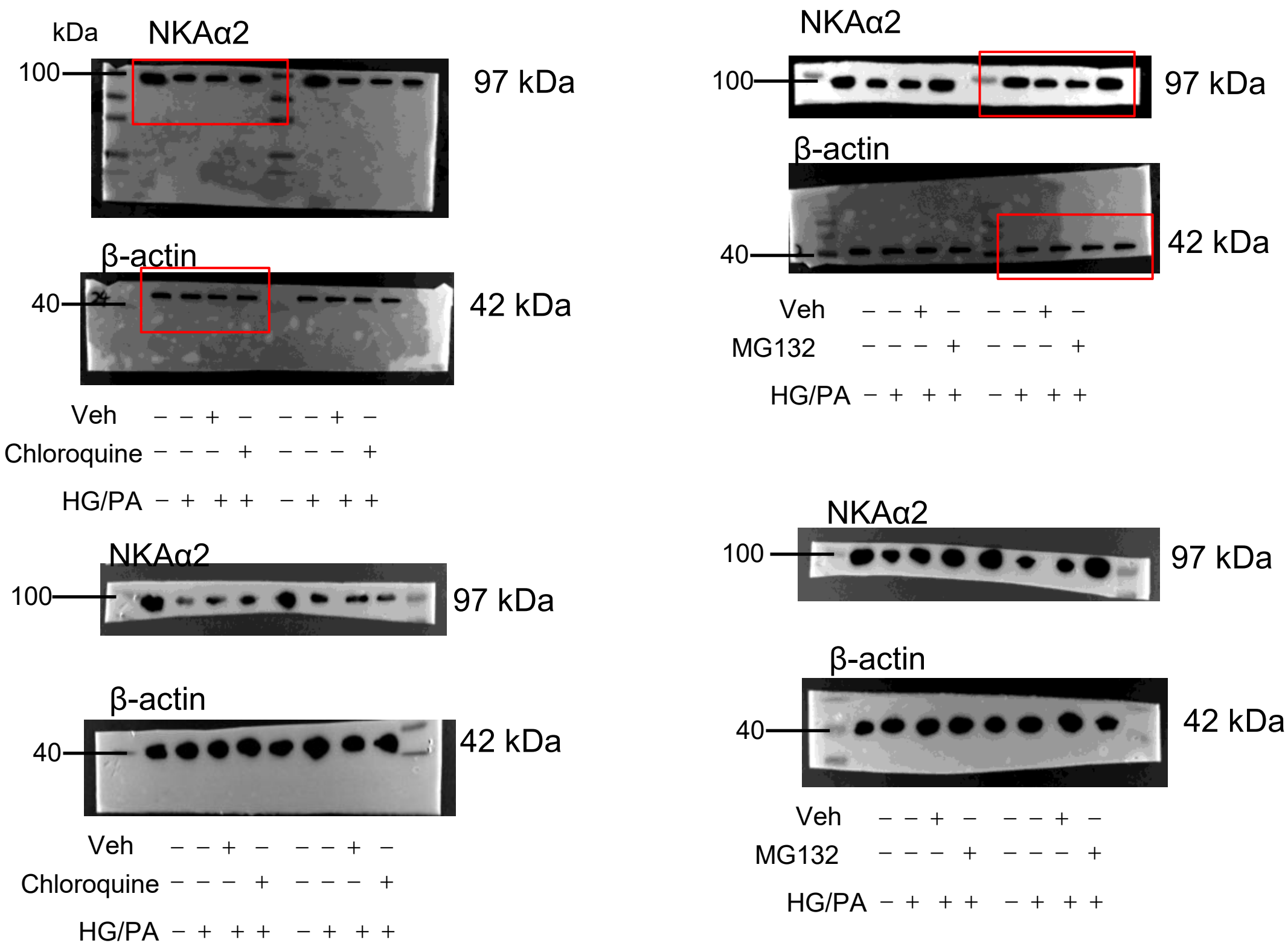

Full uncropped immunoblots in **Figure 5E-G**. The red rectangle indicates the portion of the blot displayed in the manuscript. n=4.

Full unedited gel for Figure 5H-I

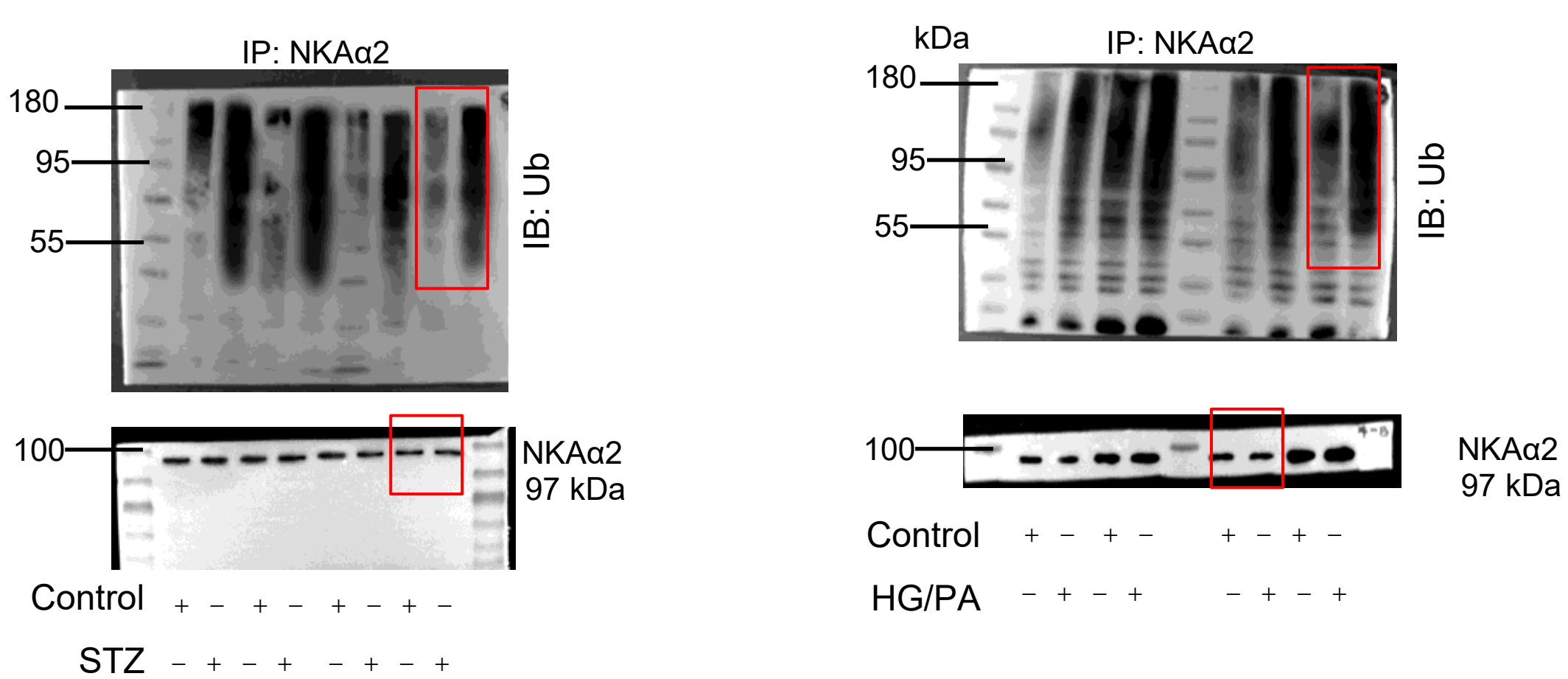

Full uncropped immunoblots in **Figure 5H-I**. The red rectangle indicates the portion of the blot displayed in the manuscript. n=4.

Full unedited gel for Figure 5J

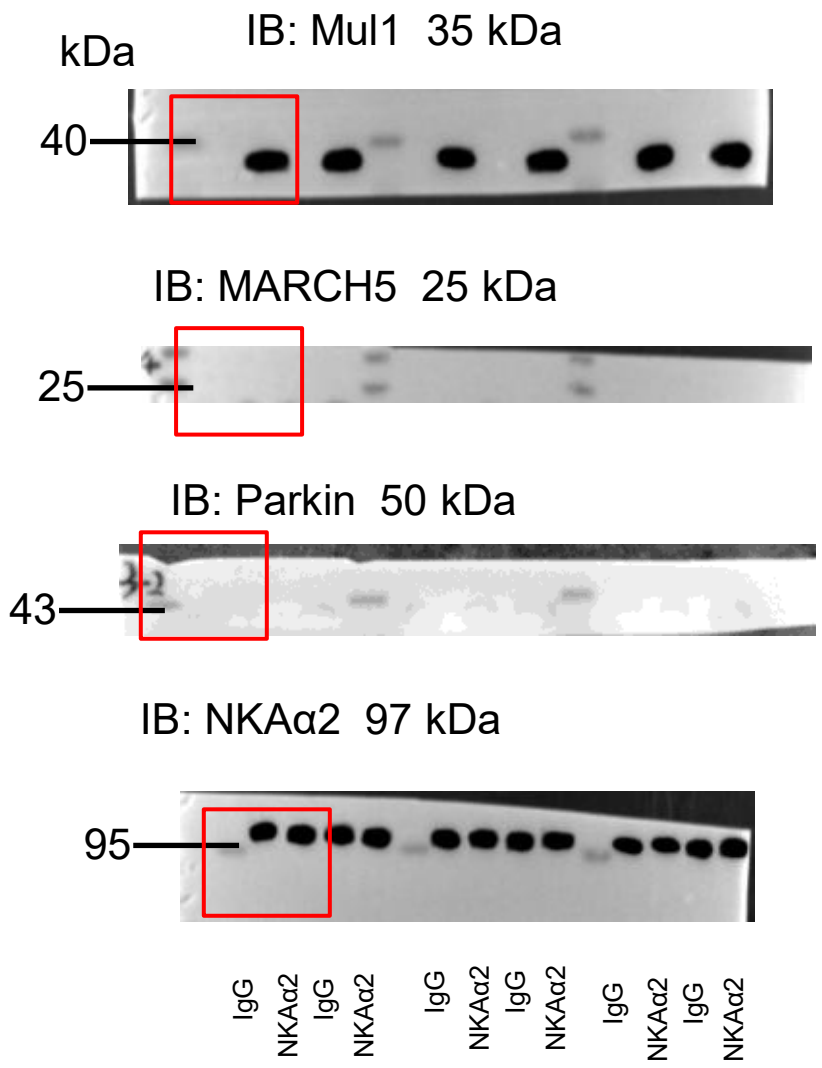

Full uncropped immunoblots in **Figure 5J**. The red rectangle indicates the portion of the blot displayed in the manuscript. n=6.

Full unedited gel for Figure 5K

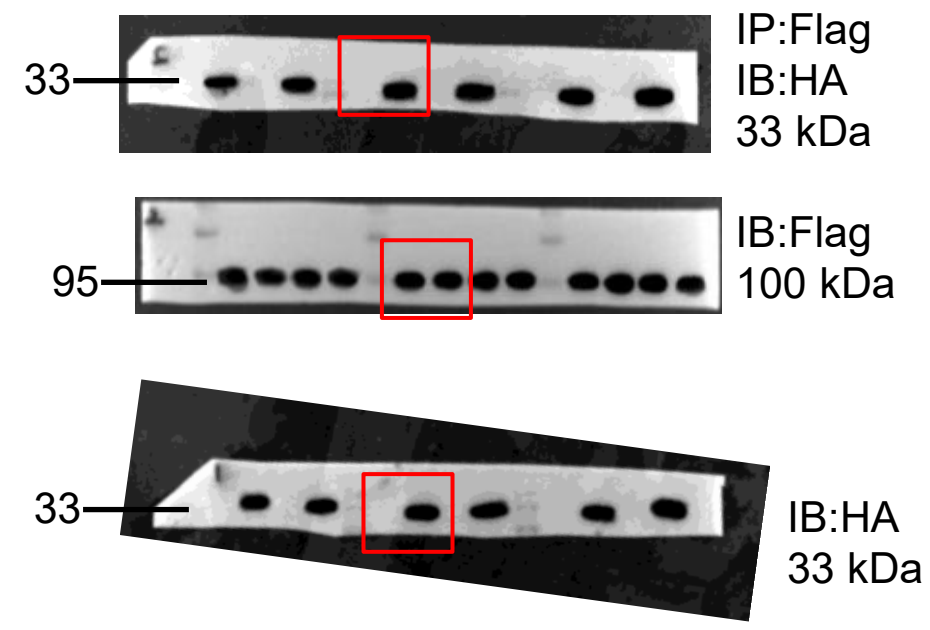

Full uncropped immunoblots in **Figure 5K**. The red rectangle indicates the portion of the blot displayed in the manuscript. n=6.

Full unedited gel for Figure 6E

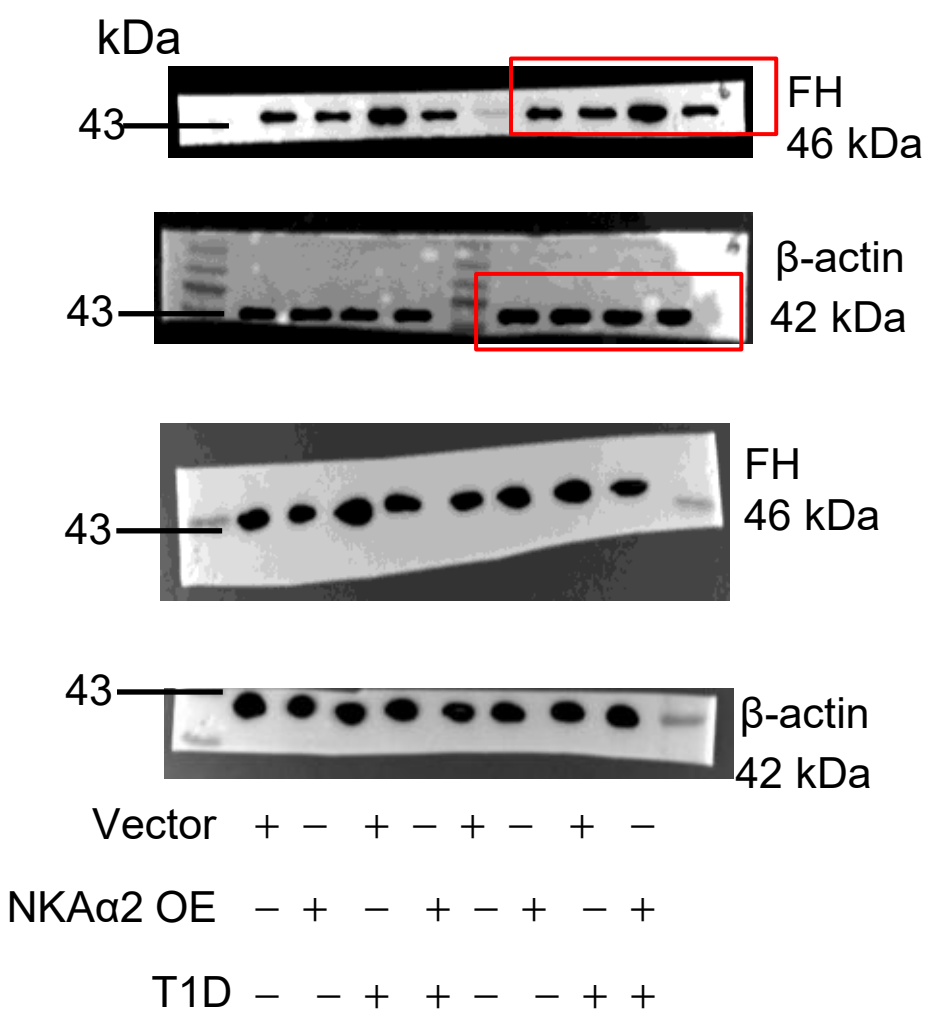

Full uncropped immunoblots in **Figure 6E**. The red rectangle indicates the portion of the blot displayed in the manuscript. n=4.

Full unedited gel for Figure 6H

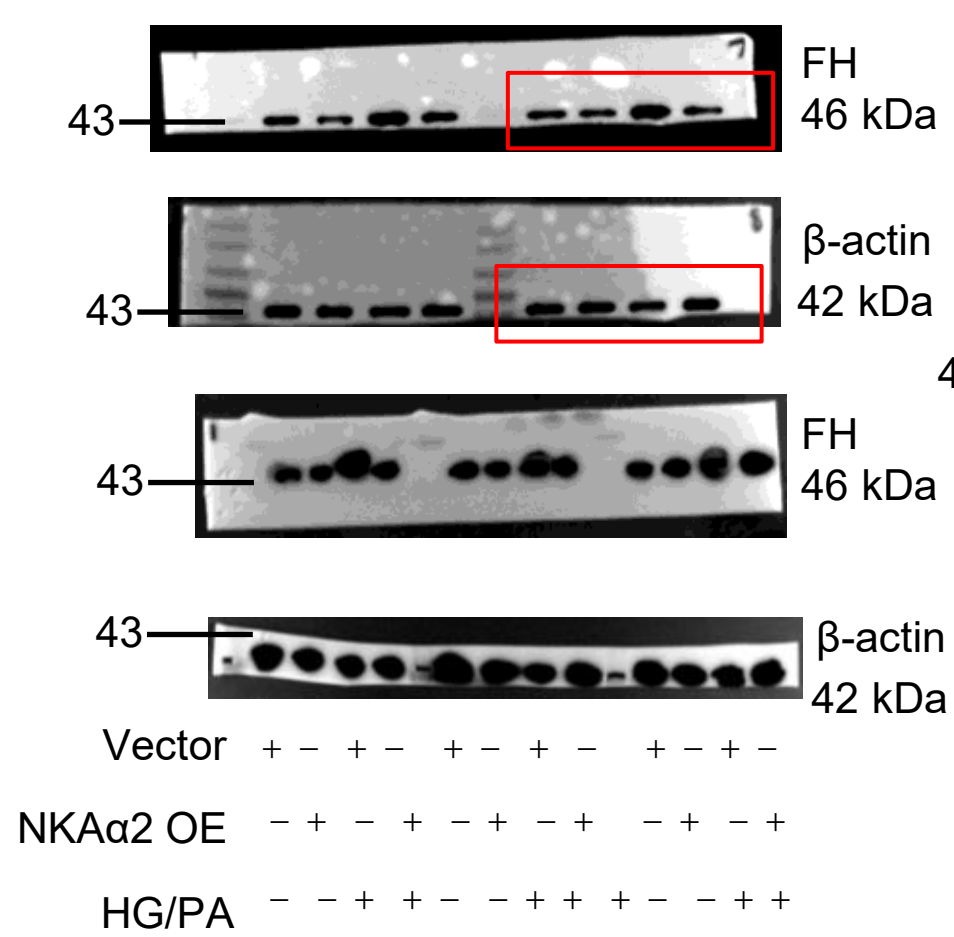

Full uncropped immunoblots in **Figure 6H**. The red rectangle indicates the portion of the blot displayed in the manuscript. n=5.

Full unedited gel for Figure 6K

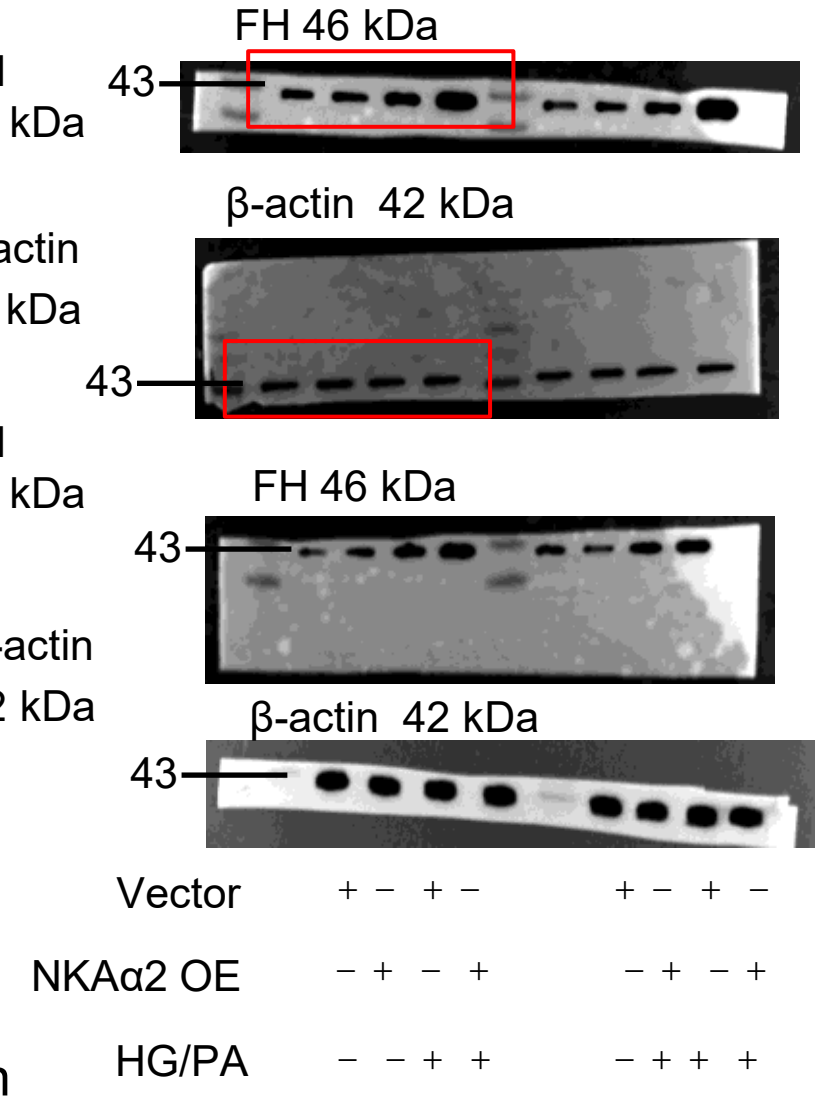

Full uncropped immunoblots in **Figure 6K**. The red rectangle indicates the portion of the blot displayed in the manuscript. n=4.

Full unedited gel for Figure S3A

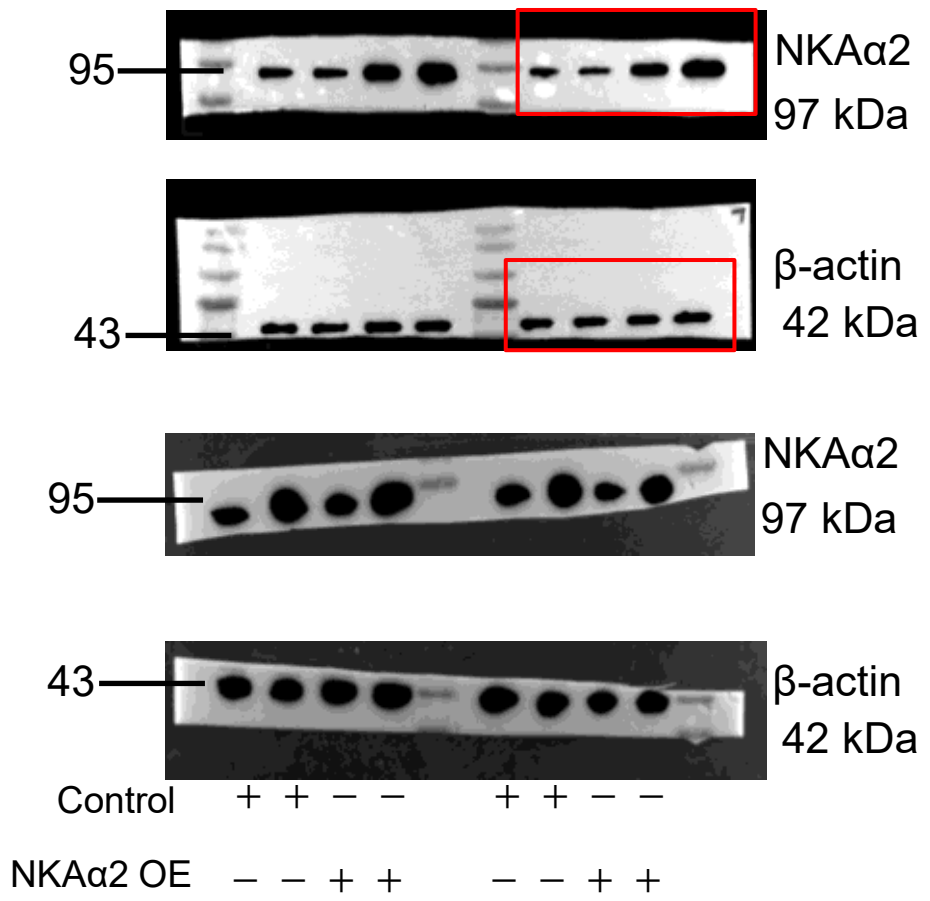

Full uncropped immunoblots in **Figure S3A**. The red rectangle indicates the portion of the blot displayed in the manuscript. n=8.

Full unedited gel for Figure S4I

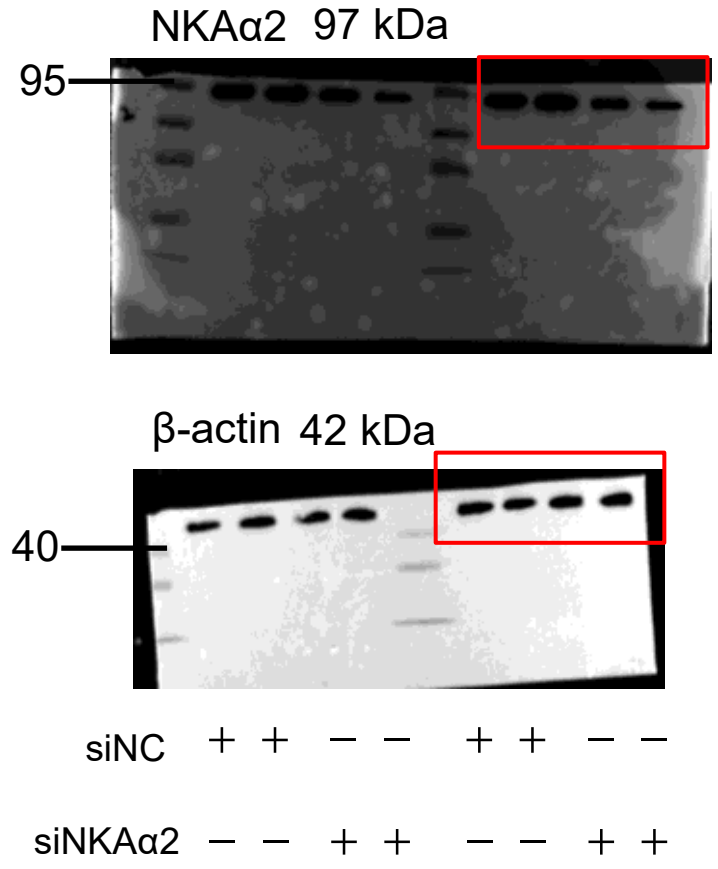

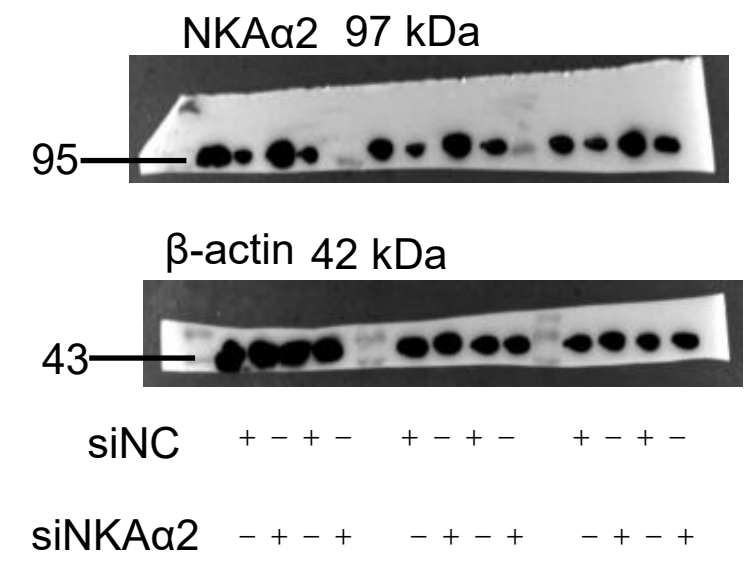

Full uncropped immunoblots in **Figure S4C**. The red rectangle indicates the portion of the blot displayed in the manuscript. n=10.

Full unedited gel for Figure S4F

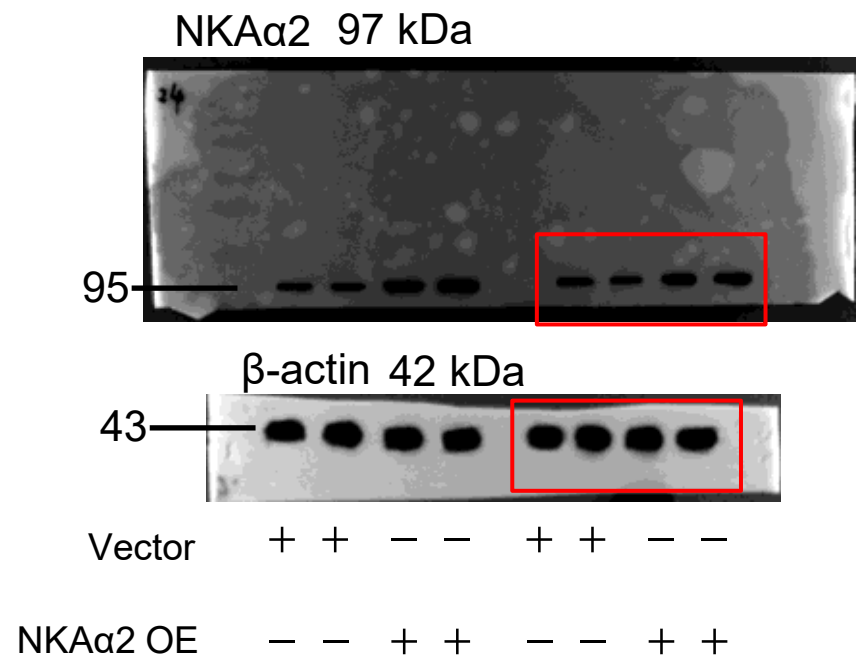

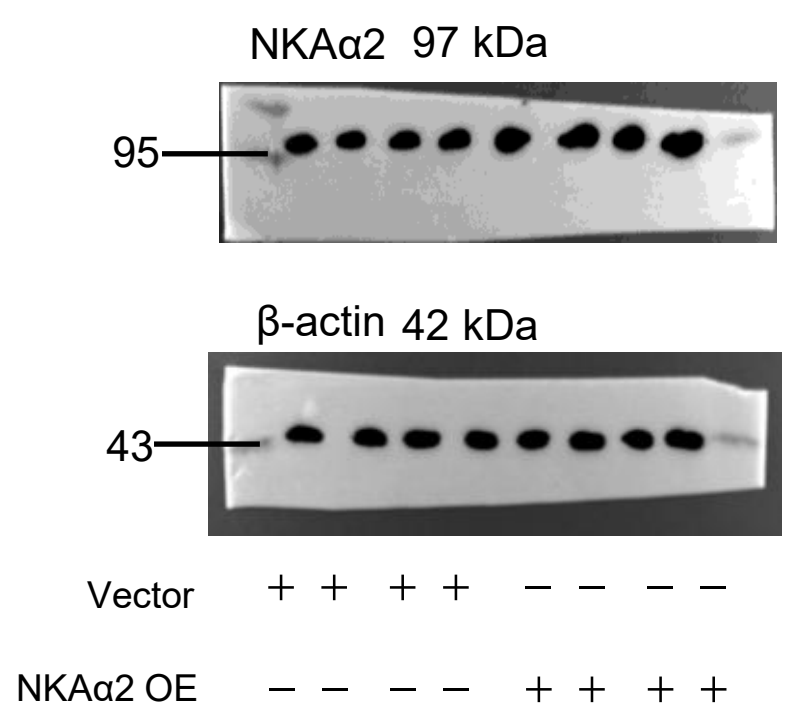

Full uncropped immunoblots in **Figure S4A**. The red rectangle indicates the portion of the blot displayed in the manuscript. n=8.

Full unedited gel for Figure S5B

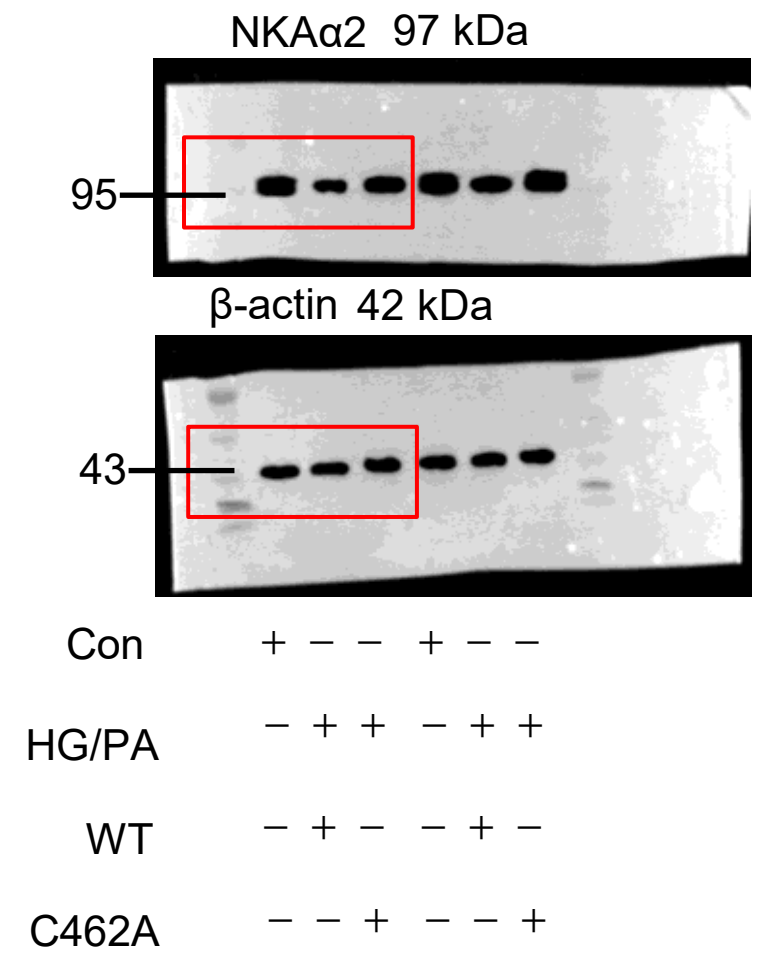

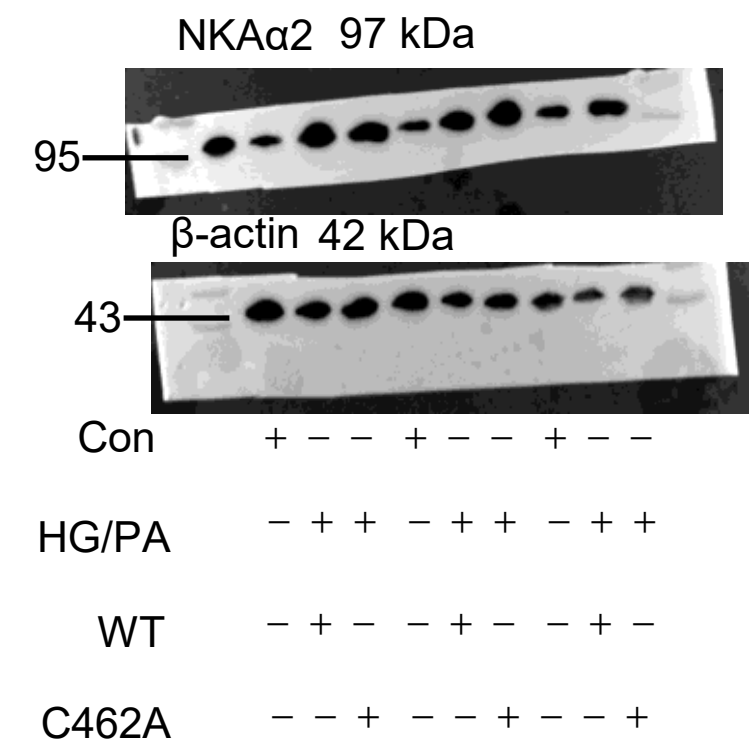

Full uncropped immunoblots in **Figure S5B**. The red rectangle indicates the portion of the blot displayed in the manuscript. n=5.

Full unedited gel for Figure S5C

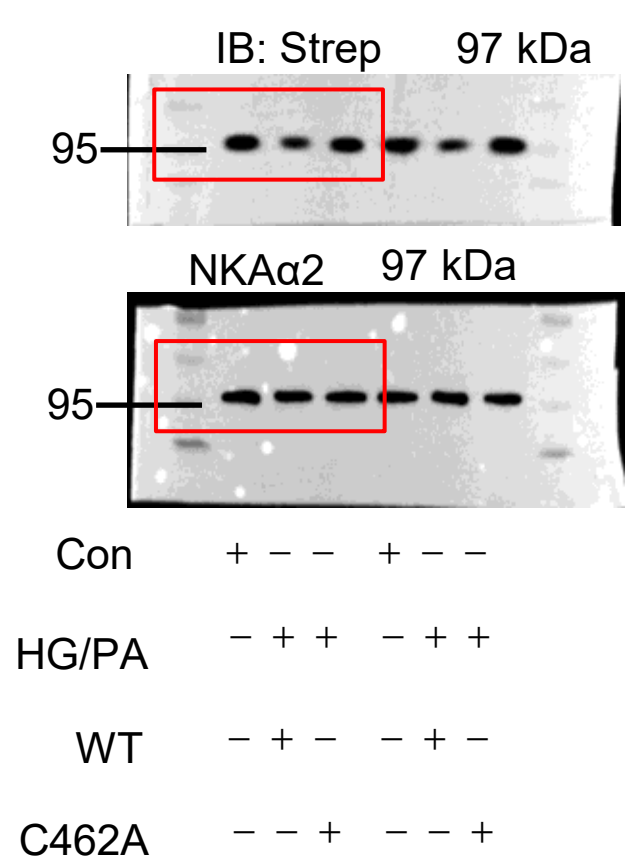

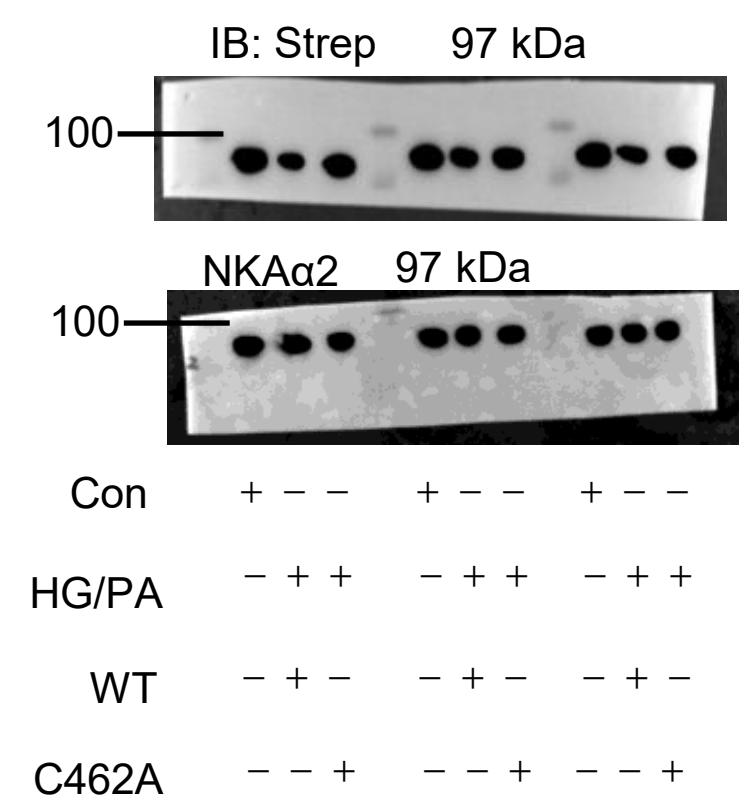

Full uncropped immunoblots in **Figure S5C**. The red rectangle indicates the portion of the blot displayed in the manuscript. n=5.

Full unedited gel for Figure S5D

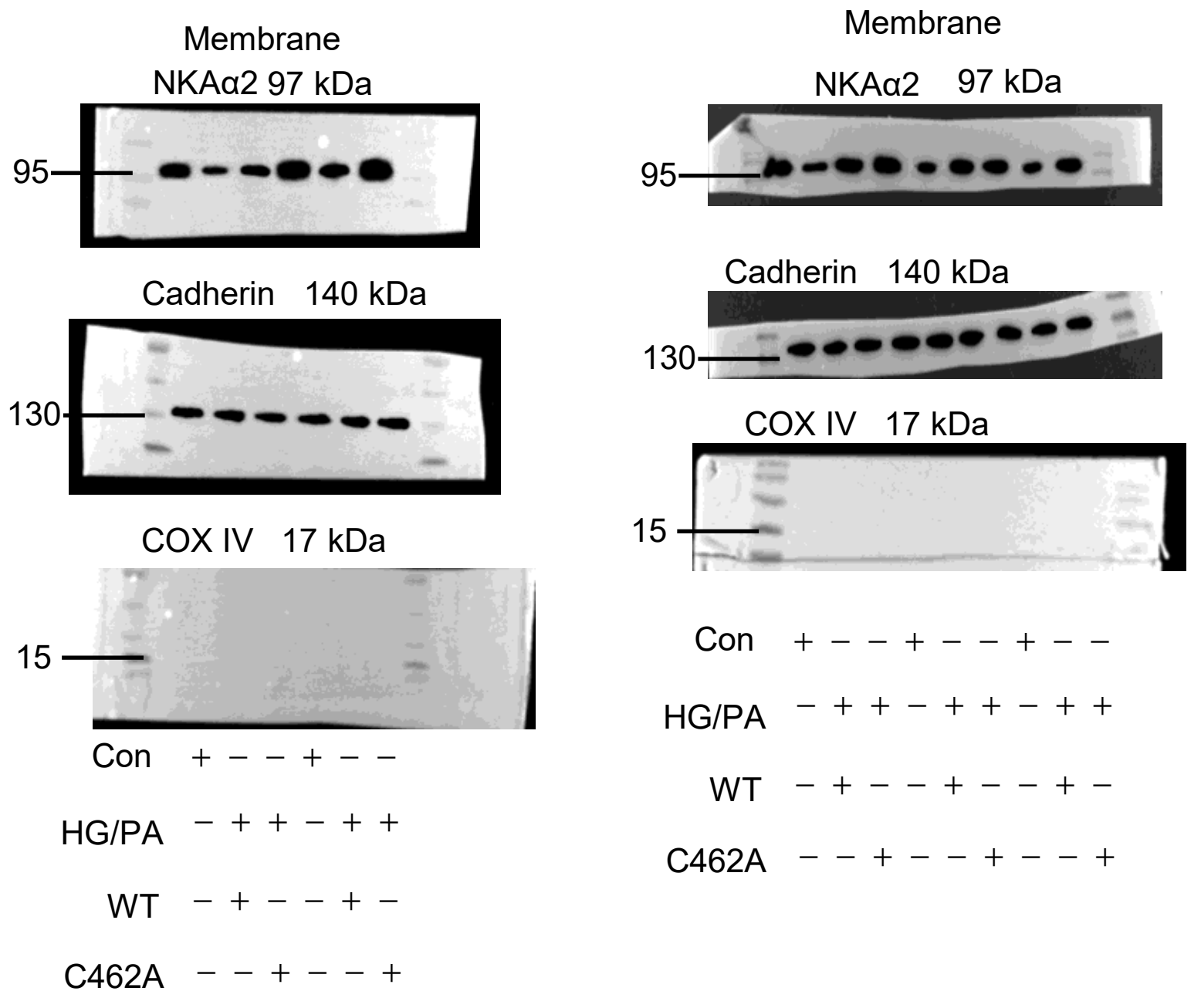

Full uncropped immunoblots in **Figure S5D**. The red rectangle indicates the portion of the blot displayed in the manuscript. n=5.

Full unedited gel for Figure S5E

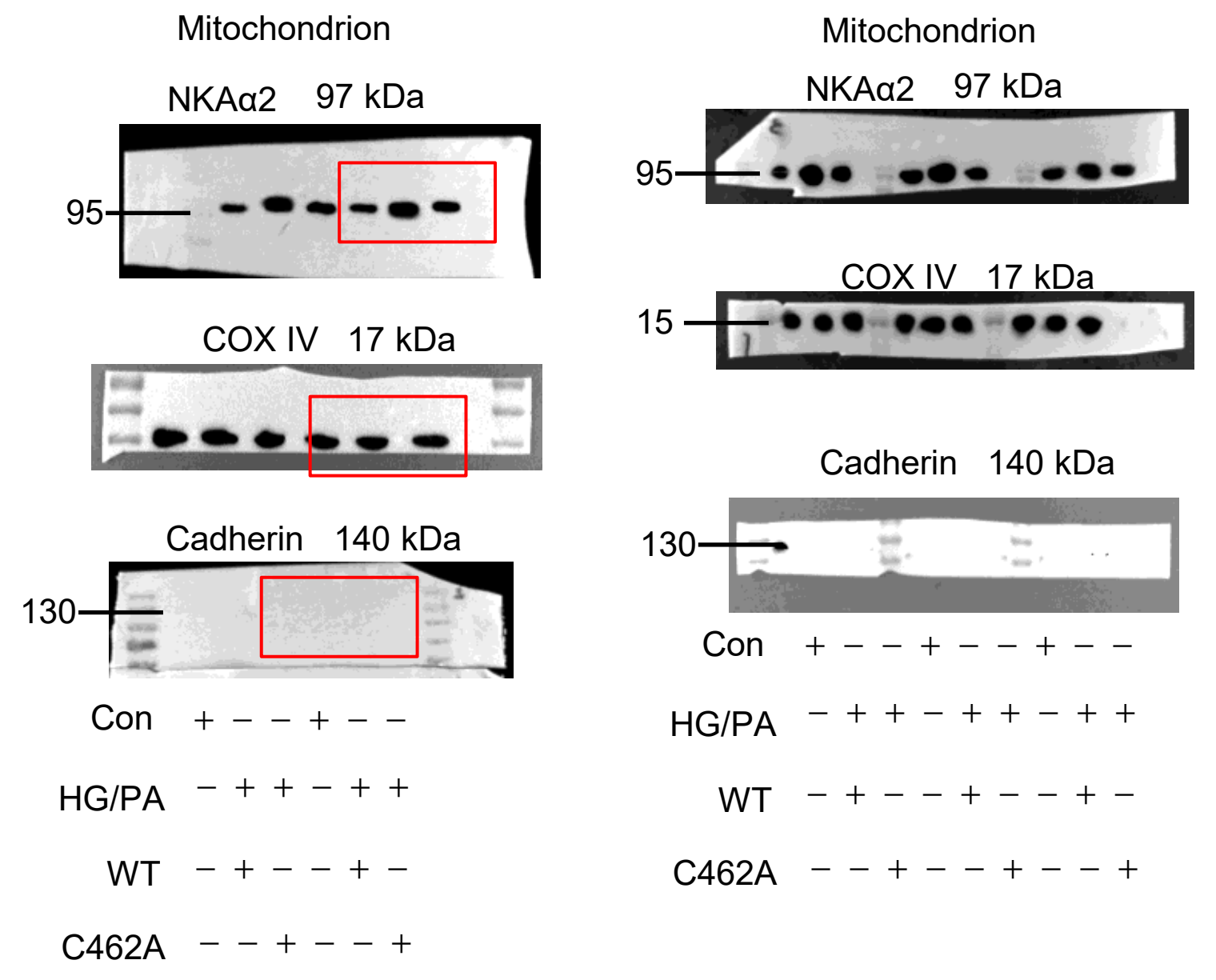

Full uncropped immunoblots in **Figure S5E**. The red rectangle indicates the portion of the blot displayed in the manuscript. n=5.

Full unedited gel for Figure S5F

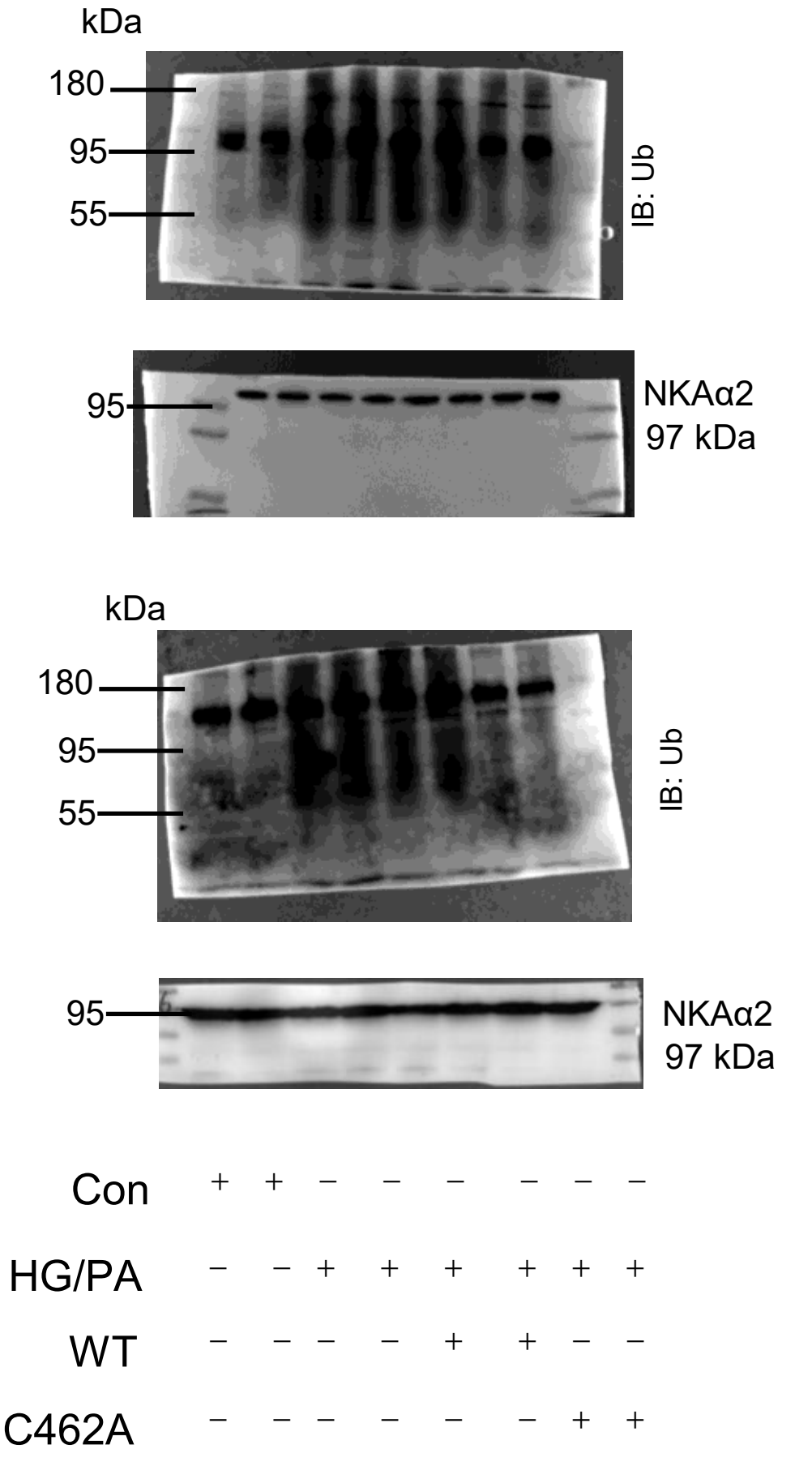

Full uncropped immunoblots in **Figure S5F**. The red rectangle indicates the portion of the blot displayed in the manuscript. n=4.

Full unedited gel for Figure S7E

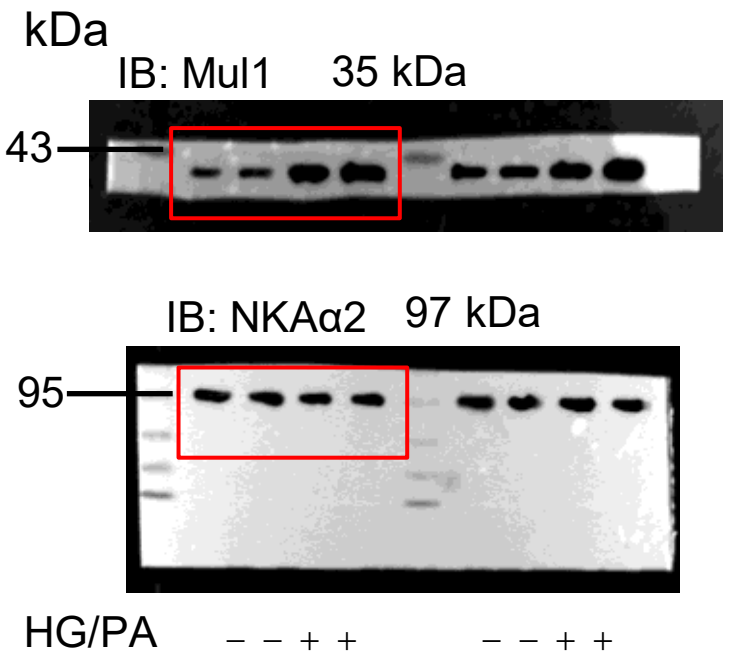

Full uncropped immunoblots in **Figure S6E**. The red rectangle indicates the portion of the blot displayed in the manuscript. n=4.

Full unedited gel for Figure S7F

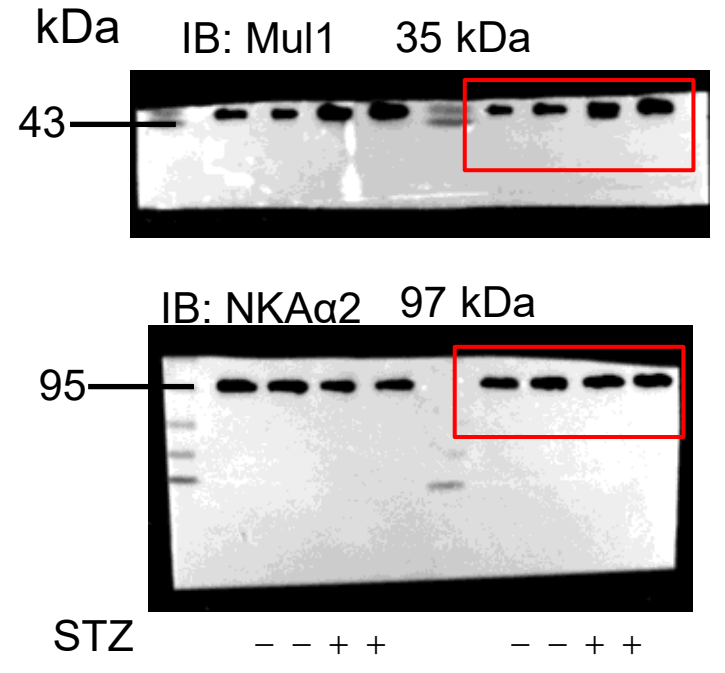

Full uncropped immunoblots in **Figure S6F**. The red rectangle indicates the portion of the blot displayed in the manuscript. n=4.

Full unedited gel for Figure S9A

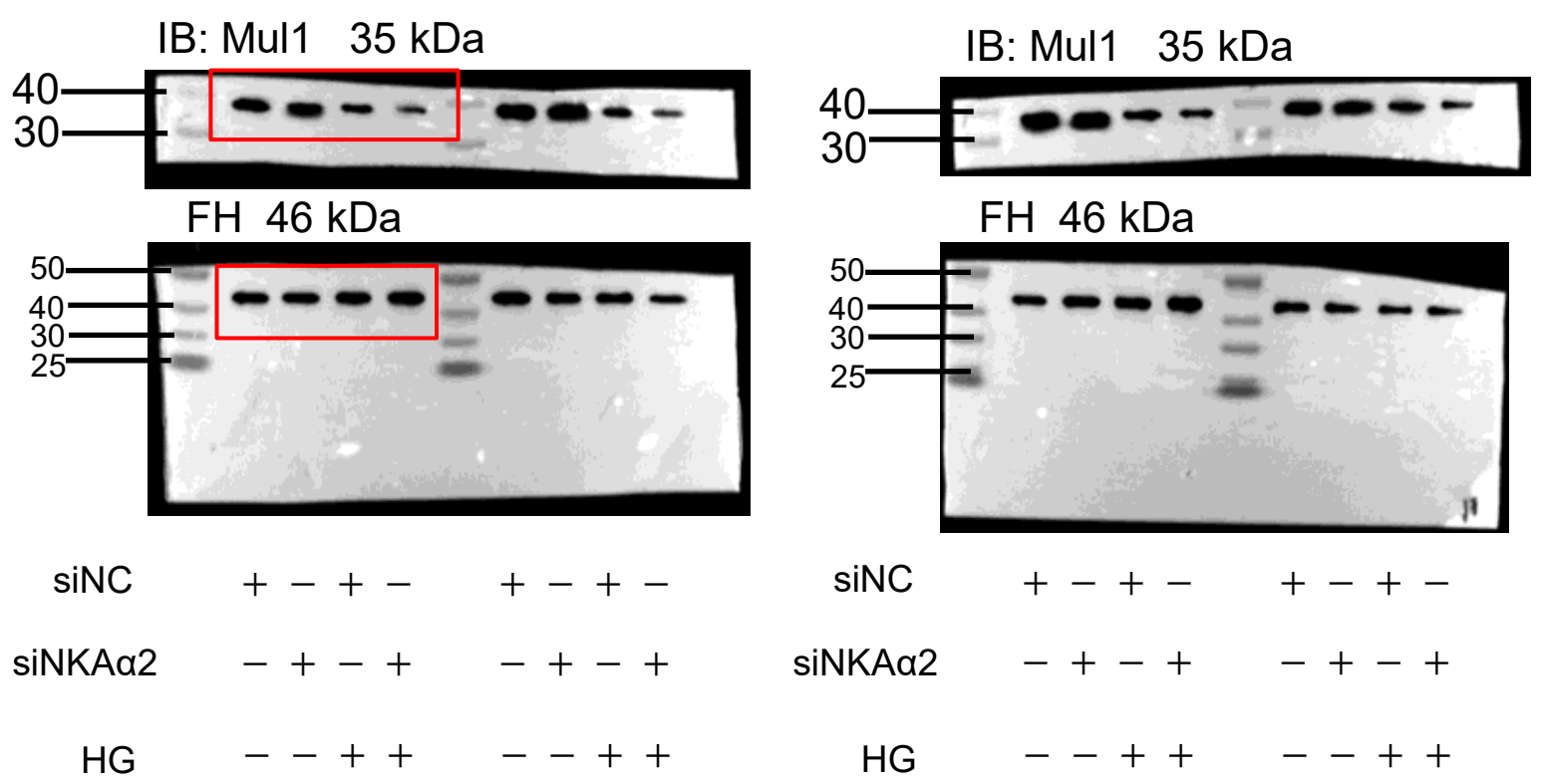

Full uncropped immunoblots in **Figure S8A**. The red rectangle indicates the portion of the blot displayed in the manuscript. n=4.

Full unedited gel for Figure S9B

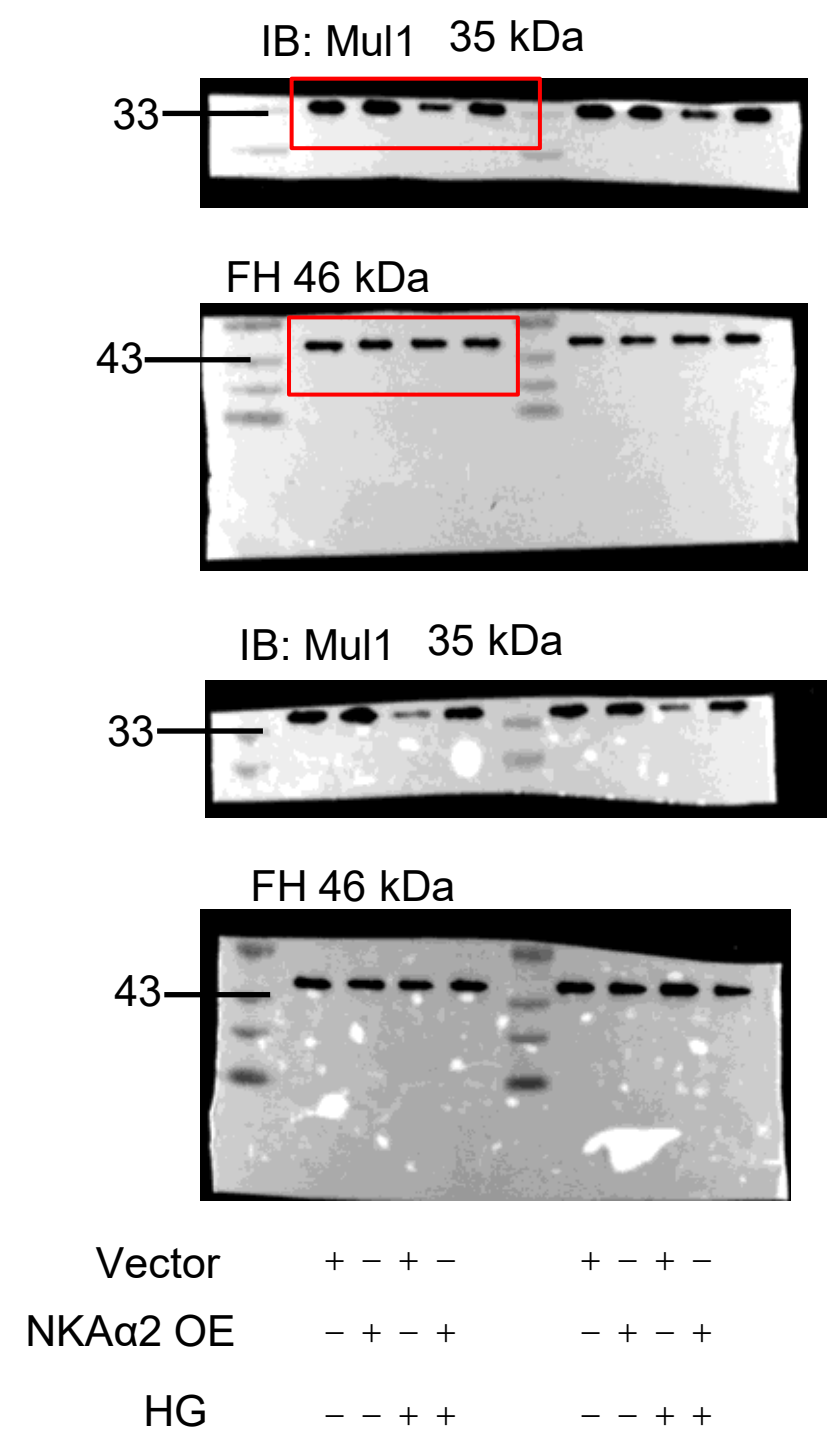

Full uncropped immunoblots in **Figure S8B**. The red rectangle indicates the portion of the blot displayed in the manuscript. n=4.
