# Supplementary material for: Palmitoylation-driven upregulation of NKAα2 improves diabetic cardiomyopathy: a role for the fumarate hydratase/fumarate metabolic pathway
Source: Cell Death Discov. 2026 Jul 29;12:425. doi: 10.1038/s41420-026-03275-9 (PMC13696732; doi:10.1038/s41420-026-03275-9)
Supplement: Supplementary file 2 [file 41420_2026_3275_MOESM1_ESM.docx]

**Supplementary data**

**Fig. S1.** **The differences in NKAα2 expression levels in different human cells**. (A) The expression of NKAα2 in different human organs. (B-C) In the human hearts, NKAα2 is primarily detected in cardiomyocytes.

**Fig. S2.** **NKAα2 is downregulated in hearts from STZ-treated mice and HG/PA-exposed cardiomyocytes.** (A) Representative immunofluorescence staining for NKAα2 in normal hearts and hearts from STZ-treated mice. (B) Representative immunohistochemical staining for IgG in normal hearts.

**Fig. S3. Overexpression of NKAα2 reduces inflammation and apoptosis in diabetic mice.** (A) Representative blots and quantitative analysis of NKAα2 and relative mRNA level of NKAα2 in the mouse hearts. (B) NKA activity. (C) Representative immunofluorescence staining for F4/80 and TUNEL staining in cardiac sections. (D) Quantitative analysis of F4/80 staining. (E) Quantitative analysis of TUNEL staining. (F) Serum levels of IL-1β, IL-6, and TNF-α. Values were expressed as mean ± SD. The difference between two groups (Fig. S3A-S3B) was statistically analyzed by Student’s t-test. The differences among the four groups (Fig. S3D-S3F) were statistically analyzed by two-way ANOVA with Tukey’s multiple comparisons test. **P*＜0.05 *vs*. Control. †*P*＜0.05 *vs*. STZ.

**Fig. S4. Expression of NKAα2 in cultured cardiomyocytes.** (A) Representative immunofluorescence staining for α-actinin in NRCMs. (B) Representative immunofluorescence staining for IgG in NRCMs. (C) NKA activity. (D) Relative mRNA level of NKAα2. (E) Cell viability. (F) Representative blots and quantitative analysis of NKAα2 after overexpression. (G) Relative mRNA level of NKAα2 after overexpression. (H) NKA activity. (I) Representative blots and quantitative analysis of NKAα2 after knockdown. (J) Relative mRNA level of NKAα2 after knockdown. (K) NKA activity. Values were expressed as mean ± SD. The differences among the five groups (Fig. S4C-S4E) were statistically analyzed by one-way ANOVA with Bonferroni’s post-hoc test. The difference between two groups (Fig. S4F-S4K) was statistically analyzed by Student’s t-test. **P*＜0.05 *vs*. Control, Vector, or siNC.

**Fig. S5.** **Depalmitoylation promoted the ubiquitination of** **NKAα2.** (A) A single palmitoylation site of NKAα2 within its domain (Cys462) based on a defined palmitoylation motif. (B,C) The C462A mutation inhibited HG/PA-elicited downregulations of NKAα2 protein and palmitoylation. (D,E) Transfection with the C462A mutant stabilized the localization of NKAα2 at the plasma membrane. (F) The ubiquitination level of NKAα2 was blocked by the C462A mutation. (G) The C462A mutation suppressed HG/PA-induced cardiomyocyte injury assessed by LDH release. (H,I) Representative images of phalloidin staining and quantitative analysis of cardiomyocyte size. (J) Relative mRNA levels of *Nppa*, *Nppb*, *Myh7*. Values were expressed as mean ± SD. The differences among the three groups were statistically analyzed by one-way ANOVA with Bonferroni’s post-hoc test. **P*＜0.05 *vs*. Con. †*P*＜0.05 *vs*. HG/PA+WT.

**Fig. S6. The mitochondrial and membrane abundance of NKAα2 in normal and HP/PA-exposed NRCMs**. Values were expressed as mean ± SD. The difference between two groups was statistically analyzed by Student’s t-test. **P*＜0.05 *vs*. Con.

**Fig. S7. The interaction between NKAα2 and Mul1**. (A-D) Assessment of the NKAα2-Mul1 interaction by root mean square fluctuation (RMSF), radius of gyration (Rg), solvent-accessible surface area (SASA), and the number of hydrogen bonds (Hnum). (E,F) The interaction of NKAα2 with Mul1 tended to be higher in the hearts from STZ-treated mice and in HG/PA-treated cardiomyocytes. Values were expressed as mean ± SD. The difference between two groups was statistically analyzed by Student’s t-test. **P*＜0.05 *vs*. Con.

**Fig. S8. The metabolic pathway of the fumarate hydratase (FH)/fumarate pathway.**

**Fig. S9. The interaction of FH with Mul1**. (A-B) Downregulation of NKAα2 further suppressed the interaction between FH and Mul1, whereas overexpression of NKAα2 restored their binding in cardiomyocytes exposed to HG/PA. Values were expressed as mean ± SD. The differences among the four groups were statistically analyzed by two-way ANOVA with Tukey’s multiple comparisons test. **P*＜0.05 *vs*. Vector or siNC. †*P*＜0.05 *vs*. HG/PA or HG/PA+siNC.

**Fig. S10. Fumarate reduces injury in HG/PA-exposed cardiomyocytes.** (A) Effects of fumarate on cell viability. (B) Effects of fumarate on LDH release. (C,D) Effects of fumarate on cellular size. (E) Effects of fumarate on the mRNA levels of *Nppa*, *Nppb*, *Myh7*. (F) NKA activity in NRCMs. (G) NKA activity in the mouse hearts. (H) Effects of fumarate on cell viability in NKAα2-deficient cardiomyocytes under HG/PA conditions. (I) Effects of fumarate on LDH release in NKAα2-deficient cardiomyocytes under HG/PA conditions. (J,K) Effects of fumarate on cellular size in NKAα2-deficient cardiomyocytes under HG/PA conditions. (J,L) Effects of fumarate on apoptosis in NKAα2-deficient cardiomyocytes under HG/PA conditions. (M) Effects of fumarate on the mRNA levels of *Nppa*, *Nppb*, *Myh7* in NKAα2-deficient cardiomyocytes under HG/PA conditions. (N) Caspase3 activity. Values were expressed as mean ± SD. The differences among the four groups (Fig. S10A-S10G) were statistically analyzed by two-way ANOVA with Tukey’s multiple comparisons test. The differences among the four groups (Fig. S10H-S10N) were statistically analyzed by one-way ANOVA with Bonferroni’s post-hoc test. **P*＜0.05 *vs*. Control or Control (Con). †*P*＜0.05 *vs*. HG/PA or HG/PA+siNC. ‡ *P*＜0.05 *vs*. HG/PA+siNKAα2.

**Table S1.** **Primers for Real-time quantitative PCR analysis in cells**.

| Name | Sequence |
| --- | --- |
| *NKAa1* | 5′-TGCTCTCTCCTCTTTCTAGTCT-3′ (Forward) |
|  | 5′-TTGTCCCCATGTTCTGATACAG-3′ (Reverse) |
| *NKAa2* | 5′-CTAGCAGCTGTAGTTATCGTCA-3′ (Forward) |
|  | 5′-GATGAGTTATCCACCTTGCAAC-3′ (Reverse) |
| *NKAa3* | 5′-TCGTCTATGATGAGATTCGCAA-3′ (Forward) |
|  | 5′-GCTGAGGTCAATAGTAGGTCTC-3′ (Reverse) |
| *NKAβ1* | 5′-CTGTCATACGTATGGGACCTAC-3′ (Forward) |
|  | 5′-ACACTAAATAAAACAGACGGCG-3′ (Reverse) |
| *NKAβ2* | 5′-TGCCTCTTTACATCTCGGATTT-3′ (Forward) |
|  | 5′-CACCGCCTAAAAGCAAAACAAA-3′ (Reverse) |
| *NKAβ3* | 5′-AGACTATAGTGCGTGTCAGTTC-3′ (Forward) |
|  | 5′-AGACTATAGTGCGTGTCAGTTC-3′ (Reverse) |
| *NKAβ4* | 5′-GGAAATTGCAGATCGTGAATGA-3′ (Forward) |
|  | 5′-GTTGAAGTTAAGGCTATGAGCG-3′ (Reverse) |
| *Nppa* | 5′-GGCCTTTTGGCTCCCAGGCC-3′ (Forward) |
|  | 5′-CTAAGTGCCGCCCCCGCTTC-3′ (Reverse) |
| *Nppb* | 5′-TTGGGCAGAAGATAGACCGGAT-3′ (Forward) |
|  | 5′-GGTCTTCCTAAAACAACCTCA-3′ (Reverse) |
| *Myh7* | 5′-GCAGACAGAGAATGGGGAGCTGTCC-3′ (Forward) |
|  | 5′-TCGCAATCATGCCGGGCTGAC-3′ (Reverse) |
| *Col1a1* | 5'-AAAGATGGACTCAACGGTCTC-3' (Forward) |
|  | 5'-CAGGAAGCTGAAGTCATAACCA-3' (Reverse) |
| *Tgfb* | 5′-CTTCAATACGTCAGACATTCGG-3′ (Forward) |
|  | 5′-CACAGTTGACTTGAATCTCTGC-3′ (Reverse) |
| *Il1b* | 5′-AACTGTGAAATAGCAGCTTTCG-3′ (Forward) |
|  | 5′-CTGTGAGATTTGAAGCTGGATG-3′ (Reverse) |
| *Il6* | 5′-TGCACTGTCAGAAAACAATCTG-3′ (Forward) |
|  | 5′-CCAGAGCAGATTTTCAATAGGC-3′ (Reverse) |
| *Zdhhc1* | 5'-GGAAGCGGCGAGTATATAGATT-3′ (Forward) |
|  | 5'-GGATCTCTTCCATGGATTCCG-3′ (Reverse) |
| *Zdhhc2* | 5'-GTCAGCTTGTCCTCTCTTTTTG-3′ (Forward) |
|  | 5'-GCCATCACCTTGACTTGAAAAT -3′ (Reverse) |
| *Zdhhc3* | 5'-TAACCTTTGTTTGTAAGCGGTG-3′ (Forward) |
|  | 5'-CAGTTGCTGACTTACTGTGAAC-3′ (Reverse) |
| *Zdhhc4* | 5'-CATCATGATCTGCATCTTCACG-3′ (Forward) |
|  | 5'-GCTGAAGACTTCGTAGGTGTAT-3′ (Reverse) |
| *Zdhhc5* | 5'-CTTATGACAGCCTACTCACTCC-3′ (Forward) |
|  | 5'-GACAGATTGTCATAACGGACTG-3′ (Reverse) |
| *Zdhhc6* | 5'-GAAGAACTTGAAGCAGGTGTTT-3′ (Forward) |
|  | 5'-GTCTGCTTTTTGTTTCAACTGC-3′ (Reverse) |
| *Zdhhc7* | 5'-GAAGGAGTACATGGAGAGCTTG-3′ (Forward) |
|  | 5'-CATCTTTCGAATGCACCTCTTG-3′ (Reverse) |
| *Zdhhc8* | 5'-CAAGTAAGCTTAACGGTCAGTC-3′ (Forward) |
|  | 5'-GGAATCACAGCAGATGAGTAGT-3′ (Reverse) |
| *Zdhhc9* | 5'-ACTCCTTCAGAACTAATGAGGC-3′ (Forward) |
|  | 5'-AAGAGTGGGACTTGGATGTTAG-3′ (Reverse) |
| *Zdhhc11* | 5'-GAAATATGCTGCCAATGCTGTA-3′ (Forward) |
|  | 5'-GTCGATGGTAATTGCGATCAAG-3′ (Reverse) |
| *Zdhhc12* | 5'-GCTGTTACTATGCCTAAAAGCC-3′ (Forward) |
|  | 5'-TCTATGTCTTCCTTTAGGGGGA-3′ (Reverse) |
| *Zdhhc13* | 5'-TTCCAAAGGTGCTGTGATAGAT-3′ (Forward) |
|  | 5'-GTCCCTTAGAGATGAGGTATGC-3′ (Reverse) |
| *Zdhhc14* | 5'-GAAACAAGTTCTTTTGCAACGG-3′ (Forward) |
|  | 5'-GAAGAAGAGTCCACTAGTGACC-3′ (Reverse) |
| *Zdhhc15* | 5'-GGCATCTGTTACCAAGGAATTG-3′ (Forward) |
|  | 5'-GGCAACTCTTTTTCACTCAGAG-3′ (Reverse) |
| *Zdhhc16* | 5'-CTCCCTGCTCTACAATTCCTTT-3′ (Forward) |
|  | 5'-GGTAGGACACACAGATAAGCAA-3′ (Reverse) |
| *Zdhhc17* | 5'-TAGTGATTTGGCTAGTTGGGTT-3′ (Forward) |
|  | 5'-AACACCACCATACATTAACCCT-3′ (Reverse) |
| *Zdhhc18* | 5'-TTCTTCTCAATCTGGTCCATCC-3′ (Forward) |
|  | 5'-GCACAGCAGTTGGTGATAATAC-3′ (Reverse) |
| *Zdhhc19* | 5'-AAATTGCCTGTCAGTTTGTACC-3′ (Forward) |
|  | 5'-GAGACGTTAAGAGAGGGGTTAG-3′ (Reverse) |
| *Zdhhc20* | 5'-GTCAGTTGATTAAACCTGACCG-3′ (Forward) |
|  | 5'-CAGACACAAAGAACAGGAACAG-3′ (Reverse) |
| *Zdhhc21* | 5'-CACTGTGCACATTGAAGGTTTA-3′ (Forward) |
|  | 5'-TAGAACTGGCCAATGCAAATTC-3′ (Reverse) |
| *Zdhhc22* | 5'-CAATTACATCCTGGTCGTCCA-3′ (Forward) |
|  | 5'-CAATTACATCCTGGTCGTCCA-3′ (Reverse) |
| *Zdhhc23* | 5'-ACGCTAAAAGGCAATATGAAGC-3′ (Forward) |
|  | 5'-ACGCTAAAAGGCAATATGAAGC-3′ (Reverse) |
| *Zdhhc24* | 5'-GTGTGTATCCTACGTCGTGAC-3′ (Forward) |
|  | 5'-CACAGAAAGGGCCGGTAATTAT-3′ (Reverse) |
| *Apt1* | 5'-TTACTCCACAAGTGTTGAGTCA-3′ (Forward) |
|  | 5'-TCTTAGGTATCCCAGTCACGTA-3′ (Reverse) |
| *Zdhhc25* | 5'-ATGTACATTGGACTTAGCGGAA-3′ (Forward) |
|  | 5'-CTGGTGAGTGTGTGTTCTGATA -3′ (Reverse) |
| *Abhd17a* | 5'-CACCTACTCTCTGGTACCTGAA-3′ (Forward) |
|  | 5'-GAGAAGAGAACTGTGTACCTGG-3′ (Reverse) |
| *Abhd17b* | 5'-TCGATGGCTTGAACTTAGATCA-3′ (Forward) |
|  | 5'-GAAGAGGCAACATAGCTCACTA-3′ (Reverse) |
| *Abhd17c* | 5'-GAGCTCTATGCACAGTACCTAG-3′ (Forward) |
|  | 5'-GTTAAATCCAGTCAAAGCGTGT-3′ (Reverse) |
| *GAPDH* | 5'-GGAAAGCTGTGGCGTGAT-3' (Forward) |
|  | 5'-AAGGTGGAAGAATGGGAGTT-3' (Reverse) |

**Table S2.** **Primers for Real-time quantitative PCR analysis in mice**.

| Name | Sequence |
| --- | --- |
| *NKAa 1* | 5′-GAAGAAATCCATCGCTTACACC-3′ (Forward) |
|  | 5′-GTTCACAAGTTTGTCCGTTTTG-3′ (Reverse) |
| *NKAa2* | 5′-GCATCATATCAGAGGGTAACGA-3′ (Forward) |
|  | 5′-CTTCTCTGGGATTGACTTGACT-3′ (Reverse) |
| *NKAa3* | 5′-GGTATCATCTCTGAGGGTAACG-3′ (Forward) |
|  | 5′-GTTCTGCAGAATCTCGTCAATC-3′ (Reverse) |
| *NKAβ1* | 5′-GAAGGACGACATGATTTTCGAG-3′ (Forward) |
|  | 5′-CACTCGGTTGAGCTTGATAATG-3′ (Reverse) |
| *NKAβ2* | 5′-TCGTCTTCTATGGTTTCCTCAC-3′ (Forward) |
|  | 5′-GTCGTTGTAAGGTTCCAAGAAC-3′ (Reverse) |
| *NKAβ3* | 5′-CCAAAGGAAGAAAACGCAACTA-3′ (Forward) |
|  | 5′-ATGGCACTCAACTGTTACTTCT-3′ (Reverse) |
| *NKAβ4* | 5′-CCGTGATCACCCTGTTCATATA-3′ (Forward) |
|  | 5′-GTTGAAGTTAAGGCTATGAGCG-3′ (Reverse) |
| *Nppa* | 5′-ACCTGCTAGACCACCTGGAG-3 ′(Forward) |
|  | 5′-CCTTGGCTGTTATCTTCGGTACCGG-3′ (Reverse) |
| *Nppb* | 5′- GAGGTCACTCCTATCCTCTGG-3′ (Forward) |
|  | 5′- GCCATTTCCTCCGACTTTTCTC-3′ (Reverse) |
| *Myh7* | 5′- CCGAGTCCCAGGTCAACAA-3′ (Forward) |
|  | 5′- CTTCACGGGCACCCTTGGA-3′ (Reverse) |
| *Il1b* | 5'-CAACTGTTCCTGAACTCAACTG-3' (Forward) |
|  | 5'-GAAGGAAAAGAAGGTGCTCATG-3' (Reverse) |
| *Il6* | 5′-CTCCCAACAGACCTGTCTATAC-3′ (Forward) |
|  | 5′-CCATTGCACAACTCTTTTCTCA-3′ (Reverse) |
| *Tgfb* | 5'-CCAGATCCTGTCCAAACTAAGG-3' (Forward) |
|  | 5'-CTCTTTAGCATAGTAGTCCGCT-3' (Reverse) |
| *Col1a1* | 5'-AGGCTTCAGTGGTTTGGATG-3' (Forward) |
|  | 5'-CACCAACAGCACCATCGTTA-3' (Reverse) |
| *Tnfa* | 5'-ATGTCTCAGCCTCTTCTCATTC-3' (Forward) |
|  | 5'-GCTTGTCACTCGAATTTTGAGA-3' (Reverse) |
| *Alox12* | 5'-CAAGGAGGAGGAGTTTGACTT-3' (Forward) |
|  | 5'-GAACTGTGATGAGGTTGCAGAA-3' (Reverse) |
| *Cybb* | 5'-GACAGGAACCTCACTTTCCATA-3' (Forward) |
|  | 5'-TGAAGAGATGTGCAATTGTGTG-3' (Reverse) |
| *GAPDH* | 5'-TCATCAACGGGAAGCCCATC-3' (Forward) |
|  | 5'-CTCGTGGTTCACACCCATCA-3' (Reverse) |

**Table S3. Biochemical characteristics and echocardiographic data in mice from different groups.**

| Characteristics | Con | NKAα2 OE | STZ | STZ+NKAα2 OE |
| --- | --- | --- | --- | --- |
| Heart rate (/min) | 472.4±35.9 | 474.2±38.6 | 479.2±34.9 | 468.7±29.7 |
| HbA1c (%) | 4.4±0.4 | 4.3±0.5 | 12.4±2.1* | 11.8±1.7* |
| Serum insulin (ng/ml) | 0.89±0.078 | 0.92±0.084 | 0.44±0.056* | 0.48±0.053* |
| Total cholesterol (mmol/l) | 2.61±0.22 | 2.59±0.26 | 3.82±0.32* | 3.76±0.39* |
| Triacylglycerols (mmol/l) | 1.18±0.14 | 1.21±0.18 | 2.76±0.23* | 2.64±0.27* |
| LVIDD (mm) | 3.59±0.32 | 3.58±0.39 | 4.55±0.48* | 3.81±0.37† |
| LVPWD (mm) | 1.68±0.15 | 1.66±0.14 | 0.82±0.079* | 1.35±0.12† |
| LVIDS (mm) | 2.16±0.21 | 2.19±0.23 | 3.34±0.27* | 2.36±0.21† |
| LVAPWD (mm) | 1.59±0.16 | 1.62±0.18 | 0.80±0.078* | 1.55±0.13† |

Notes: HbA1c, Hemoglobin A1C; LVEF, left ventricular ejection fraction; LVIDD, left ventricular internal diameter in diastolic end; left ventricular internal diameter in systolic end; LVFS, left ventricular fraction shortening; LVIDS, LVPWD, left ventricular posterior wall thickness in diastolic end; LVAPWD, left ventricular anterior wall thickness in diastolic end. n =6. *P < 0.05 versus Con, †P < 0.05 versus STZ.

**Table S4. Biochemical characteristics and echocardiographic data in mice from different groups.**

| Characteristics | Con | Fumarate | STZ | STZ+Fumarate |
| --- | --- | --- | --- | --- |
| Heart rate (/min) | 478.1±36.7 | 466.3±37.2 | 468.1±38.1 | 470.1±28.4 |
| HbA1c (%) | 4.3±0.5 | 4.4±0.4 | 12.3±1.9* | 11.7±1.8* |
| Serum insulin (ng/ml) | 0.88±0.082 | 0.91±0.088 | 0.45±0.049* | 0.47±0.051* |
| Total cholesterol (mmol/l) | 2.63±0.23 | 2.57±0.24 | 3.87±0.31* | 3.79±0.34* |
| Triacylglycerols (mmol/l) | 1.15±0.16 | 1.18±0.19 | 2.79±0.26* | 2.69±0.21* |
| LVIDD (mm) | 3.54±0.34 | 3.55±0.31 | 4.52±0.41* | 3.78±0.32† |
| LVPWD (mm) | 1.65±0.14 | 1.67±0.16 | 0.81±0.072* | 1.36±0.11† |
| LVIDS (mm) | 2.17±0.18 | 2.18±0.17 | 3.36±0.21* | 2.32±0.19† |
| LVAPWD (mm) | 1.63±0.17 | 1.61±0.19 | 0.78±0.083* | 1.51±0.11† |

Notes: HbA1c, Hemoglobin A1C; LVEF, left ventricular ejection fraction; LVIDD, left ventricular internal diameter in diastolic end; left ventricular internal diameter in systolic end; LVFS, left ventricular fraction shortening; LVIDS, LVPWD, left ventricular posterior wall thickness in diastolic end; LVAPWD, left ventricular anterior wall thickness in diastolic end. n =6. *P < 0.05 versus Con, †P < 0.05 versus STZ.

**Table S5. Biochemical characteristics and echocardiographic data in mice from different groups.**

| Characteristics | Con | STZ | STZ+  NKAα2 OE | STZ+NKAα2 OE+FH OE |
| --- | --- | --- | --- | --- |
| Heart rate (/min) | 468.4±38.1 | 478.2±31.8 | 461.3±31.2 | 465.2±34.5 |
| HbA1c (%) | 4.5±0.5 | 12.8±0.6* | 13.1±1.2* | 13.4±1.9* |
| Serum insulin (ng/ml) | 0.93±0.082 | 0.46±0.056 | 0.49±0.052* | 0.47±0.058* |
| Total cholesterol (mmol/l) | 2.47±0.28 | 3.95±0.29* | 3.87±0.39* | 3.99±0.42* |
| Triacylglycerols (mmol/l) | 1.15±0.18 | 2.97±0.22* | 2.85±0.22* | 2.75±0.26* |
| LVIDD (mm) | 3.52±0.34 | 4.58±0.31* | 3.78±0.31† | 4.37±0.43‡ |
| LVPWD (mm) | 1.63±0.16 | 0.78±0.08* | 1.42±0.09† | 0.86±0.07‡ |
| LVIDS (mm) | 2.11±0.19 | 3.38±0.28* | 2.39±0.21† | 3.21±0.25‡ |
| LVAPWD (mm) | 1.63±0.11 | 0.79±0.06* | 1.52±0.11† | 0.83±0.07‡ |

Notes: HbA1c, Hemoglobin A1C; LVEF, left ventricular ejection fraction; LVIDD, left ventricular internal diameter in diastolic end; left ventricular internal diameter in systolic end; LVFS, left ventricular fraction shortening; LVIDS, LVPWD, left ventricular posterior wall thickness in diastolic end; LVAPWD, left ventricular anterior wall thickness in diastolic end. n =6. *P < 0.05 versus Con, †P < 0.05 versus STZ, ‡P < 0.05 versus STZ+NKAα2 OE.
